# Recent advances in gene delivery nanoplatforms based on spherical nucleic acids

**DOI:** 10.1186/s12951-024-02648-5

**Published:** 2024-07-01

**Authors:** Nazila Valatabar, Fatemeh Oroojalian, Mina Kazemzadeh, Amir Ali Mokhtarzadeh, Reza Safaralizadeh, Amirhossein Sahebkar

**Affiliations:** 1 Faculty of Natural Science, University of Tabriz, Tabriz, Iran; 2 Department of Medical Nanotechnology, School of Medicine, North Khorasan University of Medical Sciences, Bojnurd, Iran; 3 Natural Products and Medicinal Plants Research Center, North Khorasan University of Medical Sciences, Bojnurd, Iran; 4 Immunology Research Center, Tabriz University of Medical Science, Tabriz, Iran; 5 Department of Animal Biology Faculty of Natural Science, University of Tabriz, Tabriz, Iran; 6 Biotechnology Research Center, Pharmaceutical Technology Institute, Mashhad University of Medical Sciences, Mashhad, Iran; 7 Applied Biomedical Research Center, Mashhad University of Medical Sciences, Mashhad, Iran

## Abstract

Gene therapy is a therapeutic option for mitigating diseases that do not respond well to pharmacological therapy. This type of therapy allows for correcting altered and defective genes by transferring nucleic acids to target cells. Notably, achieving a desirable outcome is possible by successfully delivering genetic materials into the cell. In-vivo gene transfer strategies use two major classes of vectors, namely viral and nonviral. Both of these systems have distinct pros and cons, and the choice of a delivery system depends on therapeutic objectives and other considerations. Safe and efficient gene transfer is the main feature of any delivery system. Spherical nucleic acids (SNAs) are nanotechnology-based gene delivery systems (i.e., non-viral vectors). They are three-dimensional structures consisting of a hollow or solid spherical core nanoparticle that is functionalized with a dense and highly organized layer of oligonucleotides. The unique structural features of SNAs confer them a high potency in internalization into various types of tissue and cells, a high stability against nucleases, and efficay in penetrating through various biological barriers (such as the skin, blood–brain barrier, and blood–tumor barrier). SNAs also show negligible toxicity and trigger minimal immune response reactions. During the last two decades, all these favorable physicochemical and biological attributes have made them attractive vehicles for drug and nucleic acid delivery. This article discusses the unique structural properties, types of SNAs, and also optimization mechanisms of SNAs. We also focus on recent advances in the synthesis of gene delivery nanoplatforms based on the SNAs.

## Graphical Abstract

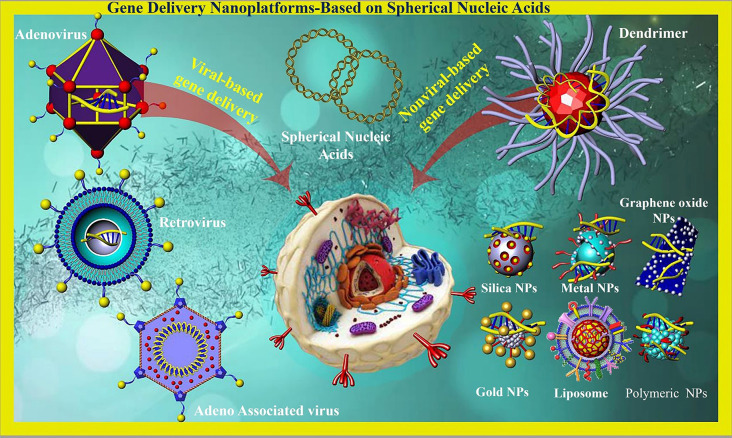

## Introduction

**Fig. 1:**
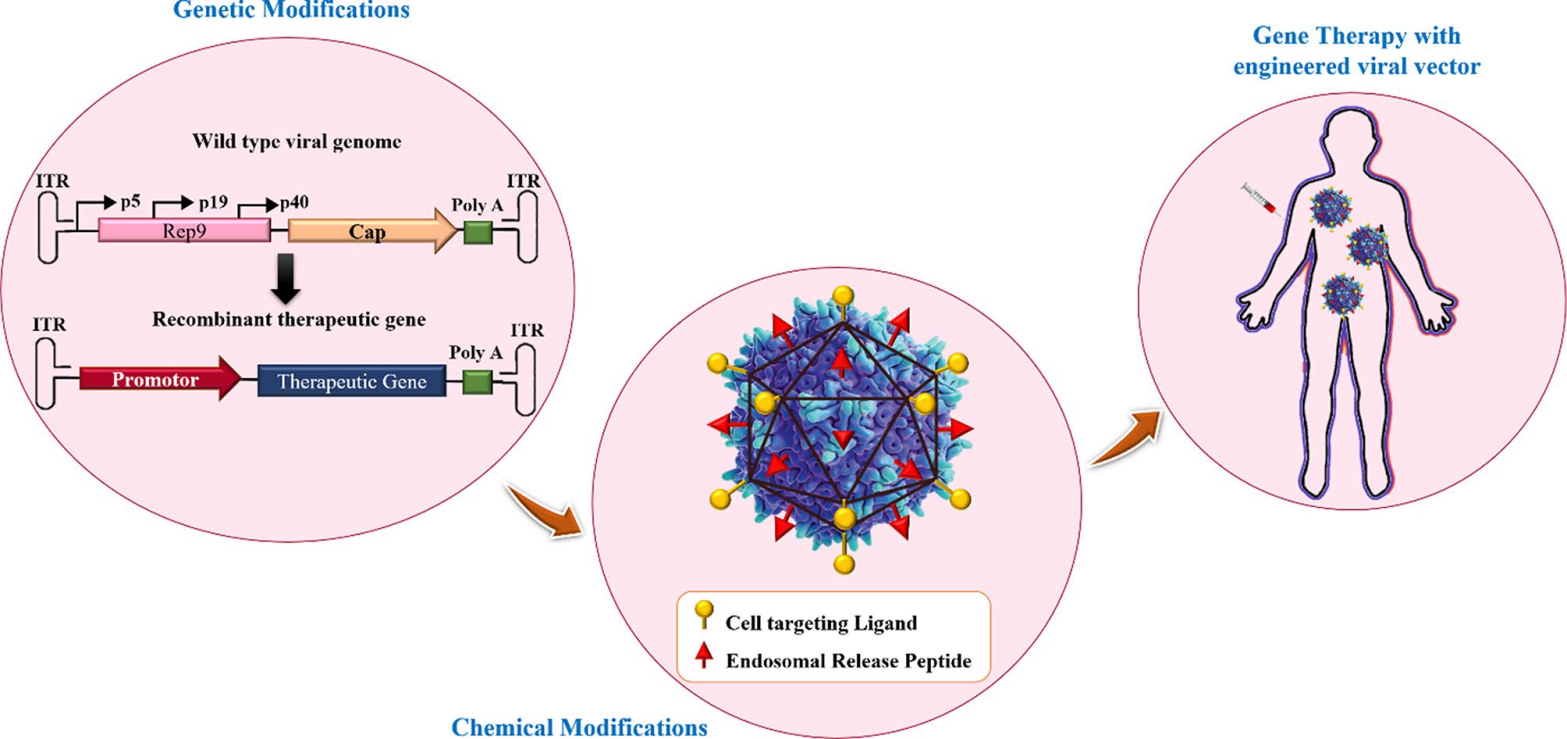
Viral vectors designing strategies for gene therapy. This figure was redrawn with permission from ref [8]

Gene therapy is one of the therapeutic approaches that can be used to cure numerous diseases, including viral infectious diseases, innate monogenetic deficiencies, and acquired multifactorial conditions like cancers [2]. Gene-based therapy includes the development of safe and effective carriers to protect therapeutic nucleic acids and facilitate their delivery to the desired site for introducing precise alterations in a specific gene function and/or directly correcting existing genetic abnormalities [3, 4]. Utilizing free nucleic acids does not show any successful results due to their low cellular uptake, rapid degradation by nuclease enzymes, interactions with serum proteins, and off-target biodistribution [3]. Consequently, different types of gene delivery systems based on both viral and nonviral vectors have been developed for introducing therapeutic agents in vivo [5–7]. Innate biological capabilities of viral vectors in infecting and replicating within host cells are two prominent and important characteristics that have made them prototypical therapeutic gene delivery vectors [8]. For the first time, two patients with adenosine deaminase (ADA) deficiency were treated through a mouse-derived retroviral vector carrying a functional ADA gene, and the use of viral vectors has received much attention since then [9]. Following that, the wide spectrum of viral carriers such as adenoviruses, adeno-associated virus (AAV), Retroviruses, Lentivirus, Herpes Simplex Viruses (HSV), Alphaviruses, Flaviviruses, Rhabdoviruses, Measles Viruses, Newcastle disease virus (NDV), Coxsackieviruses, and Poxviruses were developed for inducing temporary and/or permanent changes in the expression level of gene(s) [10]. Some of the major groups of viral vehicles employed for gene therapy are mentioned below in Table 1. In this manner, natural viruses undergo genetic modifications during which viral pathogenic genes, responsible for replicative ability, are removed and replaced with desired genes for gene therapy [8]. Moreover, the viral surface receives chemical modifications with polymers [e.g.Poly(ethylene glycol)] to protect it from immune recognition [8]. Also, the retargeting ability of vectors can be achieved by conjugating them with targeting ligands (Fig. 1) [8]. Non-integrative and less immunogenic properties of adeno-associated virus vectors (AAVs) make them to be used frequently compared to retroviruses or lentiviruses [11]. Some characteristics of viral vectors such as cargo size capacity, relatively high costs, more immunogenicity, difficulty to be synthesized on a large scale, and their invasive route of administration have promoted designing non-viral vectors as an alternative method [12] to resolve the limitations associated with their viral ounterparts [13]. Non-viral systems embrace all chemical nanocarriers that can be divided into, lipid-based (liposomes, lipoplex, Solid lipid nanoparticles [SLNs], nanostructured lipid carriers [NLC]), polymers-based (PAMAM, PPI, PEI, peptides-based (albumin), and inorganic (Carbon allotropes, Metal nanoparticles, Spherical nucleic acids, Porous particles) nanostructures. Physical methods (i.e., microinjection; microparticle bombardment, electroporation, sonoporation, magnetofection) can be used for direct transfer of exogenous nucleic acids into the cell without vectors [14–19]. The physical methods utilize different types of physical forces such as electric, magnetic, ultrasonic, and laser‑based energy for creating transient penetration in the desired cell membrane for the entrance of the desired nucleic acids into the cell [2, 6, 15, 20] (Table 1). Physical methods exhibit lower transfection efficiency than chemical gene transfer carriers [6]. So, chemical nanocarriers are more commonly used for gene delivery [6]. They usually acquire nanometric complex constructions due to compaction of negatively charged nucleic acids by polycationic nano-particles with a positive charge [2]. Therefore, these nano-complexes usually have stability against degradation and are able to enter cells via vesicular endocytic trafficking [2, 20]. Non-viral vectors are highly favored over viral vector systems due to several advantages, including lower immunogenicity, better safety, high packaging capacity, stability, structural and functional design flexibility, and easy synthesis [20]. Some properties of chemical gene delivery systems are presented in Table 1. Each gene delivery system has its benefits and limitations. So, selecting each one of them depends on its features and applications. However, an ideal gene carrier should be able to transport diverse genetic materials, protect the cargo from enzymatic degradation, along with showing non-toxicity, low or non-immunogenicity, specific tissue- and cell-targeting ability, endosomal escape, optimal gene release efficiency, and ability to transport the genetic cargo into the nucleus [3]. Further, as mentioned previously, several oligonucleotide carriers have been developed to enhance the therapeutic potential of gene delivery systems. Of the most recently developed chemical gene delivery nanoplatforms, spherical nucleic acids (SNAs) have developed as promising genetic material vehicles for gene delivery [21]. These nano-complexes contain a radial distribution of therapeutic oligonucleotide strands (e.g. miRNA, siRNA, antisense oligonucleotide, and immune-modulatory strands) which are tightly packed surrounding either hollow or solid core nanoparticles, enabling their use in various biomedical applications [21–23]. Their unique 3D structure makes them a safer and more resistant nanocarrier with low immunogenicity, allowing for reagent-free transfection and crossing biological barriers (epidermal, blood − brain barrier [BBB], and blood − tumor barrier). All these make SNAs an attractive nanocarrier for gene delivery [24]. The challenges and limitations related to gene delivery systems, and approaches to bypass them for clinical usage have been extensively reviewed. In this review, we focus on nanoplatforms of spherical nucleic acids developed to maximize DNA/RNA delivery efficiency for therapeutic applications. First, we explain SNA synthetic methods, design, cell entering, unique properties, optimizing methods, and then highlight important examples of their applications in oligonucleotide (plasmid DNA, siRNA, and miRNA) delivery for the treatment of diverse human diseases by focusing on recent clinical investigations in the field. Finally, the main purpose of this review is to give a perspective to inspire future design and development of SNAs-based delivery technologies with high efficiency for in-vivo applications.

**Table 1:** Advantages and disadvantages of various gene delivery systems

	Capacity	Advantages	Disadvantages	Ref.
		**Viral systems**		
Retrovirus	8 kb	Permanent gene expression	Less effective in vivo; high immunogenic; infects just dividing cells; High carcinogenic risk due to insertional mutagenesis	[2, 10, 11]
Lentivirus	8 kb	Permanent gene expression; transduce both dividing cells and non-dividing	Random integration into genome causes insertional mutagenesis; Probable for tumorigenesis	[2, 10, 11, 25]
Adenovirus	> 7.5 kb	Transduce both dividing and non-dividing cells; Carrie large DNA cargo (up to 38 kb); safe; high titer production	Transient gene expression; pre-existing immunity	[2, 10, 11, 26]
Adeno-associated virus	< 4 kb	Permanent gene expression; non-pathogenic; wide-ranging host and cell type	Deliver low amount of gene cargo due to its small size; Low titer production	[2, 10, 11, 27]

	Capacity	Advantages	Disadvantages	Ref.
		**Non-viral systems (Physical Methods)**		
Microinjection	Small fragments to large size fragments (up to the amount of DNA)	Very high efficacy	In vivo problematical; technically demanding; only a few cells (100–200) can be injected in one experiment;	[28, 29]
Gene gun (gene gun /Biolistic gene transfer)	Small fragments to large size fragments (up to the amount of DNA)	Good efficiency (depends on the loading of genetic material onto the particles, the size of the particle, and the timing of delivery	limited tissue depth (usually used for delivery to the skin); inflammation and damage in tissue in some applications; non-specificity (possibly non-targeted cells transfection); quantities limitation of DNA or RNA on microparticles (so, several transfections needed for tissue engineering applications.)	[17]
Electroporation	Small fragments to large size fragments (up to the amount of DNA)	Easiness; inexpensive; vector free	Invasive; Poor infiltration across (deep) tissues	[15, 30]
Magnetoporation	Small fragments to large size fragments (up to the amount of DNA)	Economic; Non-invasive; make possibly gene delivery to diverse cells (i.e.; hard-to-transfect cells, primary cells, and non or slowly dividing cells)	Poor efficiency with naked DNA	[15, 29, 30]
Sonoporation	Small fragments to large size fragments (up to the amount of DNA)	Noninvasive; high efficiency compares to ultrasound, Imaging during treatment; can be used in vivo; site-specificity;	Lower Reproducibility; Tissue damage; relatively low transfection efficiency (in vitro and in vivo)	[15, 29, 30]
Optoporation (Laser irradiation/ Photoporation)	Small fragments to large size fragments (up to the amount of DNA)	High-efficiency accuracy of the laser beam; might be better for local gene delivery;	Probability tissue damage; low accuracy; Low irradiation area; low transfection rate; limited for clinical use;	[15, 30]

	Capacity	Advantages	Disadvantages	Ref.
		**Non-viral systems (Chemical Methods**)		
Protein-based methods	Several kb (by viral capsid protein) short sequences (by dsRNA-binding proteins and modified oligonucleotides)	Low toxicity Increased stability	Protein purification	[30]
Peptide-based methods	Variable from the length of ASO or siRNA to plasmid DNA	Biocompatible and biodegradable; Low to moderate toxicity; selective targeting and barrier protection; easily synthetizes (in bacterial or mammalian cells and with SPPS technique.)	Synthesis can be expensive in some cases	[30, 31]
Lipid-based methods	Variable from the length of ASO or siRNA to plasmid DNA	Low toxicity (excluding highly cationic particles); Low immunogenicity; Easy to manufacture; biocompatibility; targeting and long-term blood circulation with Surface modification (e.g., ligands and PEGylation; respectively)	Low half-life stability on storage; Historically low transfection efficiency compared to viral vectors	[30, 32]
Polymers, dendrimers, and micelles	Variable from the length of ASO or siRNA to plasmid DNA	non-immunogenic; transient expression; high packaging capacity; Targeting possible via site-specific attachment of ligands; Biodegradability of many polymers (i.e.; chitosan, PLGA, or PLL)	Low gen delivery efficiency in-vivo; Cytotoxicity of highly cationic polymers; Biodegradability issues for certain polymers Immune response to polymers	[30, 32]
Nanoparticles (Carbon allotropes, Metal nanoparticles, Spherical nucleic acids, Porous particles)	Variable from the length of ASO or siRNA to plasmid DNA	High packaging capacity: Low cytotoxicity and non-immunogenic	Difficult in vivo degradation; Low gene delivery efficiency; toxicity (Some carbon allotropes)	[30]

## SNAs structure

**Fig. 2:**
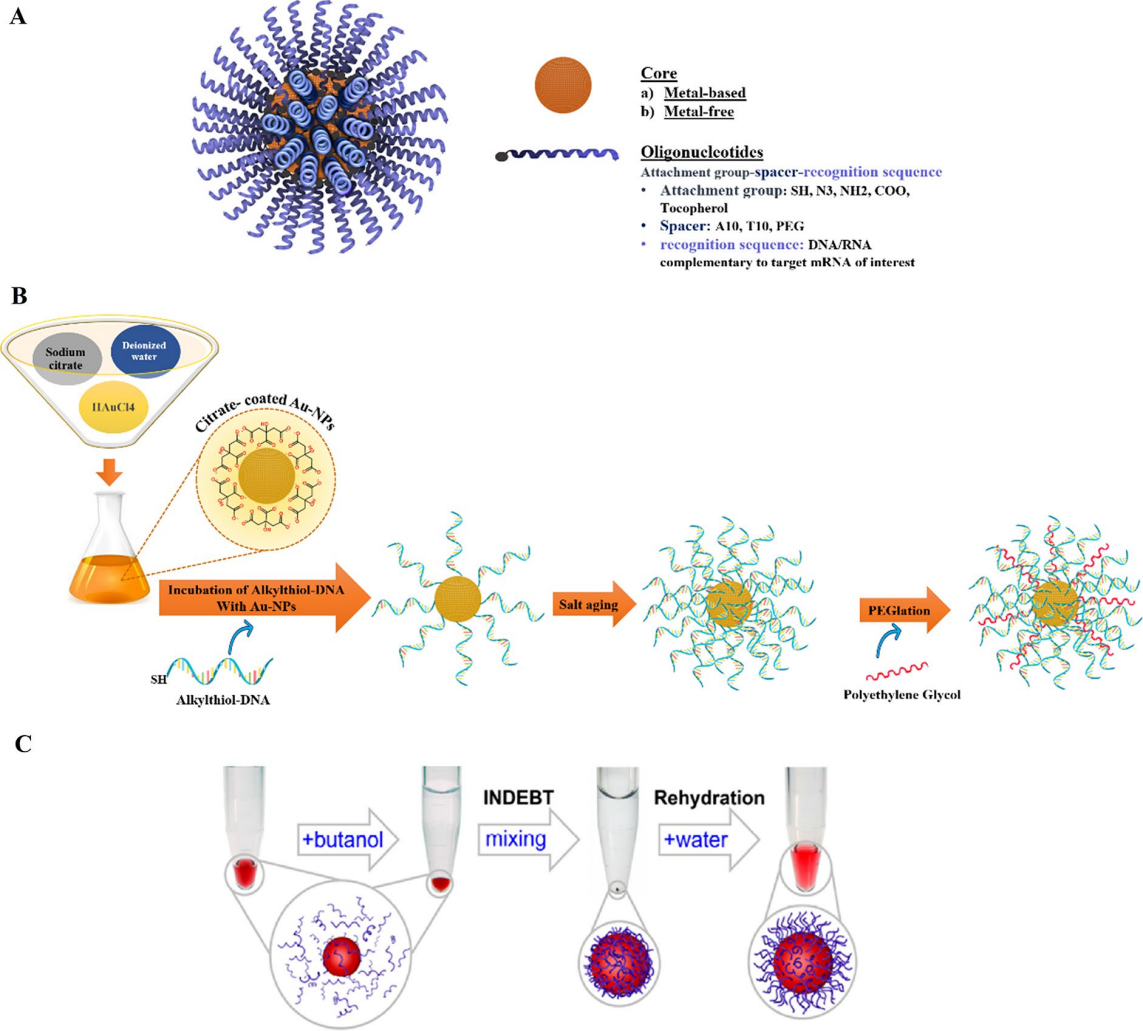
Schematic display of (**A**) A Spherical Nucleic Acid (SNA) nanoconjugate [37]. (**B**) SNAs synthetizing procedure based on Turkevich–Frens (chemical method) [41]. (**C**) SNAs synthetizing procedure based on instant dehydration in butanol (INDEBT) method [42]. This figure was redrawn with permission from the mentioned references

In 1996, spherical nucleic acids (SNAs) were introduced as a class of chemically modified nanomaterials consisting of a nano-scale particle as a core coated with highly arranged single-stranded oligonucleotides shell through thiolated linkers [33]. The core of SNAs plays a crucial role in densely assembling oligonucleotide anchors onto arrays. Traditionally, sphere-shaped gold nanoparticles (13-nm diameter) have been used as the core of the SNA structure (21). Yet, some other materials such as Au, Ag, γ-Fe_2_O_3_, quantum dots (QDs), platinum (Pt), palladium(Pd), and silica (SiO2) have also been used as the core of nanoparticles in SNAs syntheses [34]. However, the use of organic and biocompatible nanoparticle templates without long-term toxicity (i.e. liposomes, proteins, and block copolymer nanostructures) is ideal for in-vivo applications of SNAs [34]. Nowadays, SNAs, including Au core (10–15 nm) conjugants with therapeutic nucleic acids (i.e.; antisense oligonucleotides [ASOs], siRNA, miRNA), are the most commonly used SNAs for intracellular delivery and biomedical applications [35]. Also, ribozymes as highly structured and catalytic RNAs have been utilized for forming ribozyme − SNAs structures, which target and catalyze direct cleaving of a specific mRNA sequence [36]. Preparing the core in a suitable size is very important due to its subsequent impaction on the whole size of SNAs, cell entrance, and functions of the nanostructure [37]. Core-attached (single or double strand) oligonucleotides are normally 7–12 nm in dimension (25–40 bp) and consist of three regions (Fig. 2A); (1) a linker of alkyl thiol or cyclic disulfide chemical tethering group that aids oligonucleotides to bind the surface of the core; (2) the oligonucleotide recognition fragment (15–25 bp) that is complementary to the desired target sequence; (3) a spacer between the recognition region and core surface that confers more flexibility to the conjugated oligonucleotide, increases SNA stability, and improves the interactions of the recognition region with the target sequence. Oligo-ethylene glycol [OEG] or a 10-bp thymine/alanine sequence is frequently used as the spacer [33, 37].

## SNAs synthesis

Different types of techniques, including thermal, chemical, sonochemical, and electrochemical pathways, have been presented for AuNP synthesis [38]. The most frequent technique for synthesizing the gold core is Turkevich–Frens (a chemical method), in which sodium citrate is utilized for reducing chloroauric acid (HAuCl4) and creating constant Au colloids (in the 5 to 150 nm size range) with a citrate cap [33, 37, 39]. Later, by covalent attachment strategies, the shell of oligonucleotides is arranged on the AuNP at a high concentration (∼0.15–1.0 M) of NaCl aqueous solution [33]. In this procedure, at first, alkyl-thiolated oligonucleotides are blended with solutions of citrate-capped gold NPs, resulting in attaching oligonucleotides, from their alkyl thiol section to the AuNPs via gold-sulfur bonds (the S–Au binding). Second, salt (NaCl) concentration increases during alkyl thiol-DNA/RNA adsorption on the core surface (salt aging process), in which Na^+^ interacts with the negatively charged oligonucleotide phosphodiester backbone, thus increasing the density of nucleic acid strands on the AuNPs surface (∼60–80 DNA strands per core, or ∼30–45 RNA strands per core) [33, 40, 41]. Finally, thiol-polyethylene glycol (PEG) is attached for filling any unfilled spaces on the NPs surface (Fig. 2B) [41].

Yan Hao et al. described a rapid method for attaching oligonucleotides. The method is based on dehydrating AuNPs and thiolated DNA under the impact of a butanol phase. This method is called instant dehydration in butanol (INDEBT). INDEBT-based SNA synthesis consists of two steps, each of them taking a few minutes [42]. At first, an aqueous solution of a DNA/NP mixture is taken into an adjusted volume of butanol phase for complete water removal, SNA assemblages take place during this procedure. Later, rehydration with a new watery phase is accomplished for gathering SNA structures (Fig. 2C) [42].

## SNAs properties and cellular uptake

Oligonucleotide shell confers the hallmark properties of SNAs, such as rapid transfection (∼ 50 different types of mammalian cells), lack oftoxicity, easy incorporation of auxiliary agents and passing across different biological barriers (epidermal, blood−brain barrier [BBB], and blood−tumor barrier) [35, 43]. In addition, despite naked linear sequences, SNAs have not only more resistance to nuclease degradation but also higher binding stability on target sequences and lower immunogenicity [37]. So, these unique properties make SNAs desirable tools for several therapeutic purposes including, (deoxy)ribonucleotides detection, and drug/gene/protein delivery [35, 42, 43]. In contrast to viral vectors and many other synthetic delivery methods that are needed for axillary agents like complexation with nanoparticles, or cationic polymers, or the use of viral capsids for transposition into the cytoplasm, the cellular uptake of SNAs is related to construction. Primarily, the shell of oligonucleotide enables the SNA structures to internalize in an active process and through the scavenger A receptor, naturally present on the cells surface [44]. These receptors can identify the oligonucleotides array and bind them with high affinity [44]. On the contrary, other nanocarriers’ internalization process mostly relies on their complexation with positively charged transfection reagents and often entering into cells via electrostatic interaction with the plasma membrane [44]. Other features of SNAs, such as the nanoparticle size, the core of the nanoparticle, the oligonucleotides sequence, morphology of therapeutic oligonucleotide strands, the density of nucleic acids on the core material surface, and the sugar backbone can affect their uptake [33, 45]. Also, SNAs, similar to other nano-compartment carriers, interact with serum proteins and form coronae proteins, which can affect their targeting abilities and uptake properties [46].

Therefore, several modifications were introduced to improve the in-vivo behavior (cellular uptake and selective targeting of cells) of SNAs such as (1) synthesis of G-rich DNA strand which facilitates scavenger A receptor-dependent endocytosis; (2) the use of Antibody − DNA hybrids for directing SNAs to desired cells; and (3) PEGylation of the core of nanoparticle to decrease nonspecific interactions between SNAs and serum proteins [33, 46, 47]. In the following, the above items will be discussed in detail.

## The intracellular fate of SNAs

The destiny of SNAs after entering the cell can provide valuable insights for designing and producing SNAs that are minimally toxic, resistant to degradation, and capable of prolonged intracellular retention. Currently, there is limited research on the intracellular fate of SNAs [48]. In a study conducted by Xiaochen A. Wu et al., the intracellular trafficking pathway of SNAs has been evaluated inside C166 mouse endothelial cells [48]. This study indicated that SNAs enter the cell via endocytosis then they exist in late endosomes for 24 h and 16 h after the internalization of oligonucleotide strands and then are separated from the nanoparticle core surface by the activity of DNase II enzymes which reside in late endosomes. It should be noted that the entering of SNAs into lysosomes was not observed in the studied cell line. The degraded oligonucleotides are transported out of the cell but the core NP stays in the late endosome. Some SNAs escape from the endosome and are involved in gene regulation activities. These pieces of information imply that designing and synthesizing SNAs with enhanced endo-/lysosomal escaping capability could improve SNAs’ cytosolic delivery and their potential in therapeutic applications [48]. So, it has been suggested that combining materials like cell-penetrating peptides and cationic polymers with SNA structure may increase SNA cytosolic availability [21]. However, when using these materials, it’s essential to consider the balance between cytotoxicity and cytosolic traffic efficiency [21]. Furthermore, it was indicated that the design and synthesis of aggregation-induced emission photosensitizing NPs could enable the direct Endo/Lysosome escape of SNAs through light irradiation without the need for any cationic auxiliary agents [49] It is noteworthy that synthesizing SNAs with biodegradable cores or hollow-core could prevent the unwanted side effects of the core NPs on cellular function [48].

## Types of SNAs

Spherical nucleic acids can be manufactured from a diverse range of suitable nanoparticle templates, in which the Au core can be replaced with other organic composites such as organic materials (micelles and liposomes), inorganic materials (silica, silver [Ag], Iron oxide, Quantum dots [QD]), and organic-inorganic compounds (i.e., proteins and infinite coordination polymers).

### Organic composites

#### Micelles

**Fig. 3:**
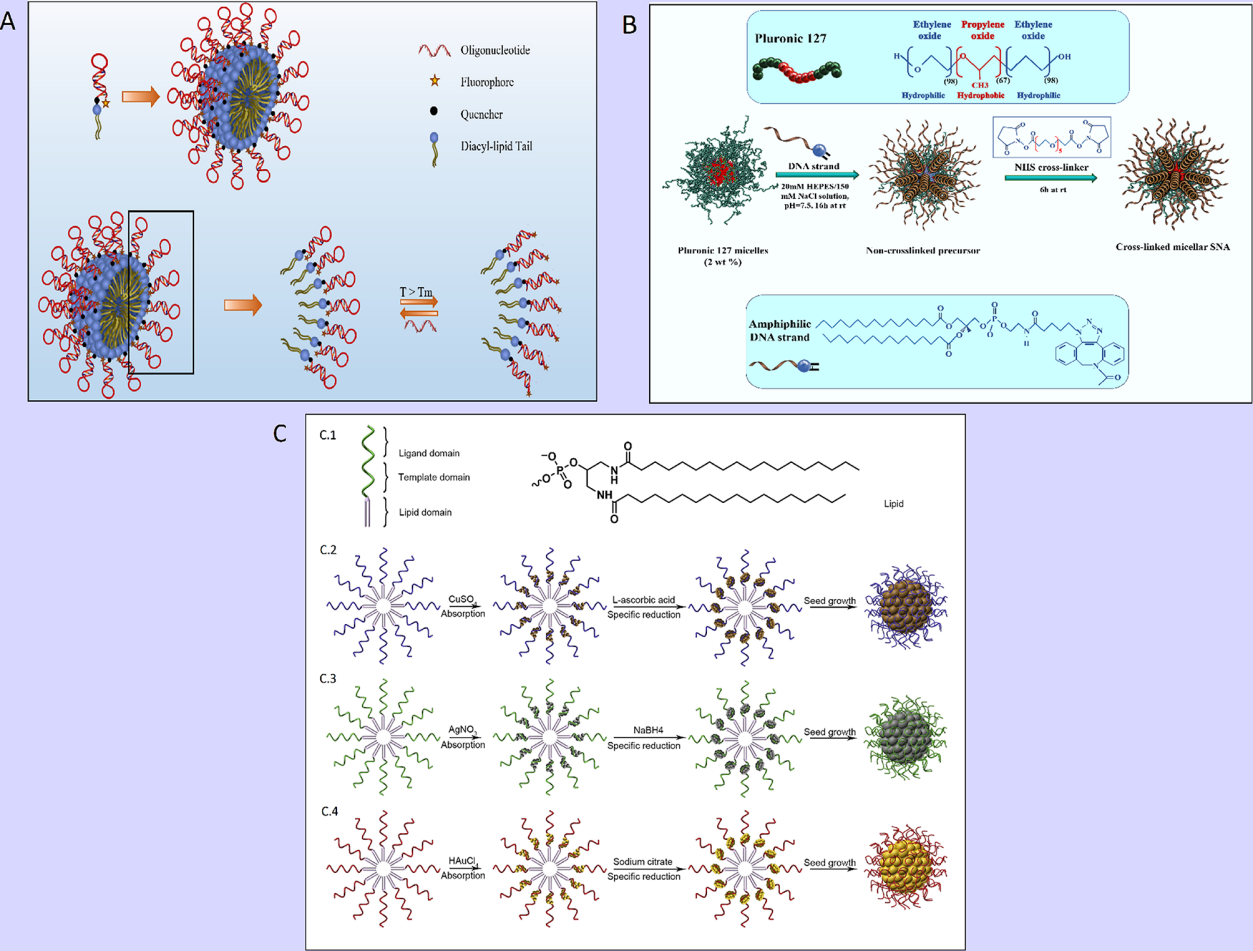
(**A**) Schematic display of a molecular beacon micelle flare (MBMF). Hairpin-shaped DNA–diacyl lipid segments self-assemble into a sphere micellar flare nanostructure, in which the hairpin-shaped DNA molecular beacon can lead to an ON/OFF switching by binding to targets, changing temperature, or degradation. This figure was redrawn with permission from ref [53]. (**B**) Schematic illustration of thermo-responsive cross-linked micellar SNAs assembling from a Pluronic F127 block copolymer core and amphiphilic DNA in a temperature-dependent condition. This figure was redrawn with permission from ref [34]. (**C**) Preparation of Metal-Conjugated ssDNA Micelles (**C.1**) with the monomer of single-stranded lipid- DNA. Right: 5ʹ end » 3ʹend: domains (lipid, template, ligand). Left: the molecular structure of lipid residue. (**C.2**) Preparation procedure of copper-crosslinked DNA micelles. (**C.3**) Preparation procedure of silver-crosslinked DNA micelles. (**C.4**) Preparation procedure of gold-crosslinked DNA micelles. Permission was received from ref [54]

The coupling of oligonucleotides to hydrophobic molecules (peptides, lipids, and polymers) can engender well-organized and supramolecular constructions, such as vesicles, monolayers, bilayers, micelles, and nanotubes [50, 51]. The beneficial properties of micelle structures, including, simplistic preparation, small size (5 ∼ 100 nm), spherical shapes, and biocompatibility, make them to be utilized for drug delivery, oligonucleotide/gene delivery, and/or biosensing and bioimaging applications [52]. Lately, three-dimensional micelle structures, comprised of a hydrophobic polymer core and a hydrophilic DNA corona, have been developed [52]. A variety of DNA-micelle structures have been established [50]. DNA micelle flares (DMFs) include micellar nanostructures, organized via hydrophobic effects between diacyllipid and single-strand DNA [53]. DMFs exhibit high cellular uptake and high enzymatic biostability. Unlike DNA block copolymer structures, DMFs can be easily synthesized and have lower critical micelle concentrations (CMC) values that make their formation quick [53]. Additionally, DMFs have higher melting temperatures and binding affinities to their target sequences compared to DNA probes not conjugated with diacyl lipids [53]. Molecular beacon micelle flares (MBMFs), comprised of either ssDNA or hairpin-shaped (molecular beacon) segments, are a suitable approach for combining detection and drug/ genes delivery [53] (Fig. 3A).

Resham J. Banga et al. reported a type of thermo-responsive cross-linked micellar SNAs, fabricating from Pluronic F127 ((poly(oxyethylene)-poly(oxypropylene)-poly(oxyethylene); PEO-PPO-PEO) micelle as the blocked core (thermo-responsive segment); the shell of amphiphilic DNA strands (consisting of lipid tails with CpG motifs [for TLR-9 stimulation] extended with dTTP, which was functionalized with C6-amines) [34]. The stability of the SNA increased by cross-linking C6-amines with PEGylated bis(sulfosuccinimidyl)suberate (Fig. 3B). This structure enables the disassembling of particles with unstable junctions from the Pluronic F127 block copolymer core during a temperature-dependent condition [34]. The results indicated that these SNAs could be manufactured and purified facilely via their thermo-responsive properties and had high stability and intracellular activity [34], delivering a potential tool for gene regulation and immune therapy [34].

The next is metal-crosslinked DNA micelle (MDM) introduced by Yifan Lyu et al., in which DNA micelles were produced by combining a template of metal ions as a hollow or solid core (i.e. copper-, silver-, and gold) into monomers of lipid-DNA (Fig. 3C) [54]. Typical SNA (AuNPs) synthesis method can occur in a boiling solution and need DNA ligand alterations (Au-S), whereas, MDM strategies use different classes of metals and have mild production conditions that take place in a one-step reaction and at normal temperature [54]. Furthermore, various types of MDMFs can be prepared for intracellular imaging by pairing MDM with detection agents [54]. Additionally, MDM exhibits better cellular intake, programable size, high incorporation capacity with oligonucleotide strands, monodispersity, and good biostability against salt-induced aggregation [54].

#### Liposome

**Fig. 4:**
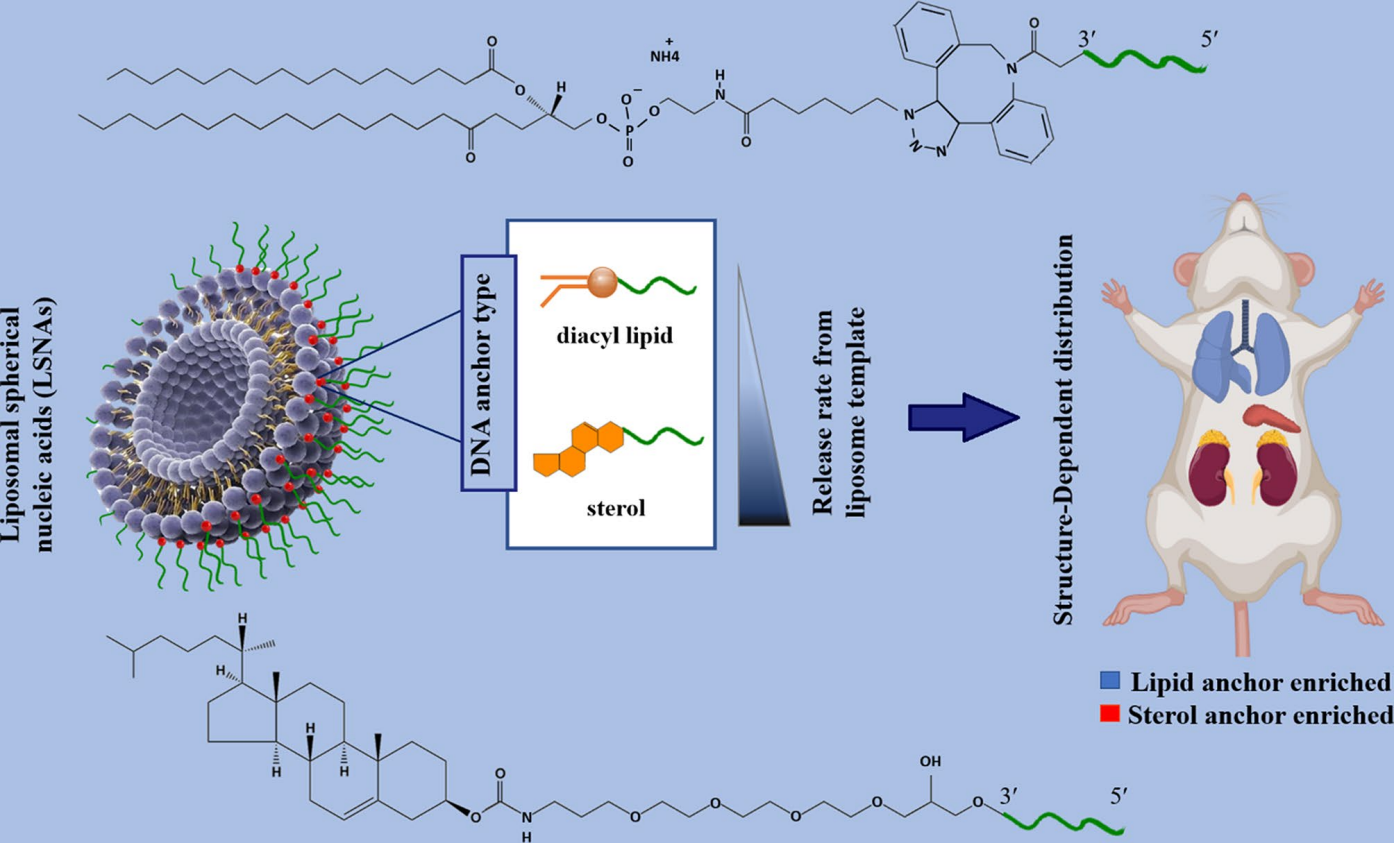
Schematic display of the biodistribution of two types (containing cholesterol tail or diacylglycerol lipid tail) of liposomal spherical nucleic acid (LSNA) conjugates This figure was redrawn with permission from ref [22]

Liposomes are sphere-shaped vesicular structures composed of an aqueous core and one or more phospholipid bilayers that can be used as delivery systems for gene therapeutics [55]. In contrast to conventional liposomal arrangements that enclose drugs or genes with lipid bilayers, in the liposomal SNAs (L-SNA), the oligonucleotide cargo is just loaded on the surface of nanostructures [56]. L-SNA was synthesized in this procedure; small unilamellar vesicles (SUVs) were initially prepared in a suitable size (30 nm diameter) via sonicating mixed suspension including lipid monomers and HEPES buffer saline [33, 57]. Then, oligonucleotides, which possess a hydrophobic tocopherol segment, can be effectively incorporated into the lipid bilayer of SUVs through hydrophobic interactions [33, 57]. Both liposomal cores and an oligonucleotide shell as two parts of L-SNA structures influence their biological activity. Ferrer et al. reported that the rational design of LSNAs’ structure affected the affinity of DNA to its liposomal core and subsequently influenced their in vivo distribution, making them to be used in tissue targeted-LSNA therapeutics [22]. The results showed that LSNA contained a cholesterol tail (low-affinity) tending to accumulate in the lungs, whereas LSNA included a diacylglycerol lipid tail (high-affinity), leading them to more accumulate in the kidneys (Fig. 4) [22]. However, both types of LSNA decreased cytokine inflammatory responses by intravenously administered oligonucleotides [22].

In another study, Sinegra et al. noted that the nature of the LNP core (increased cholesterol content) and sequences of surface-grafted oligonucleotides (G-rich sequences compared to T-rich sequences) enhanced LNP-SNA activity [58]. Also, they referred that the structural optimization of LNP-SNA decreased siRNA concentration, required for silencing mRNA (2 times) in contrast to liposome-based SNAs [58]. Moreover, biodistribution and mRNA expression profiles could be affected by the LNP-SNA designs [58]. In the case of LNP-SNAs architecture, mRNA primarily expression was seen just in the spleen (Organ-specific mRNA expression), whereas, in conventional Lipid-NPs (without DNA on the surface of NPs), mRNA expression primarily was seen in the liver with a quite low expression in the spleen [58].

#### Protein

**Fig. 5:**
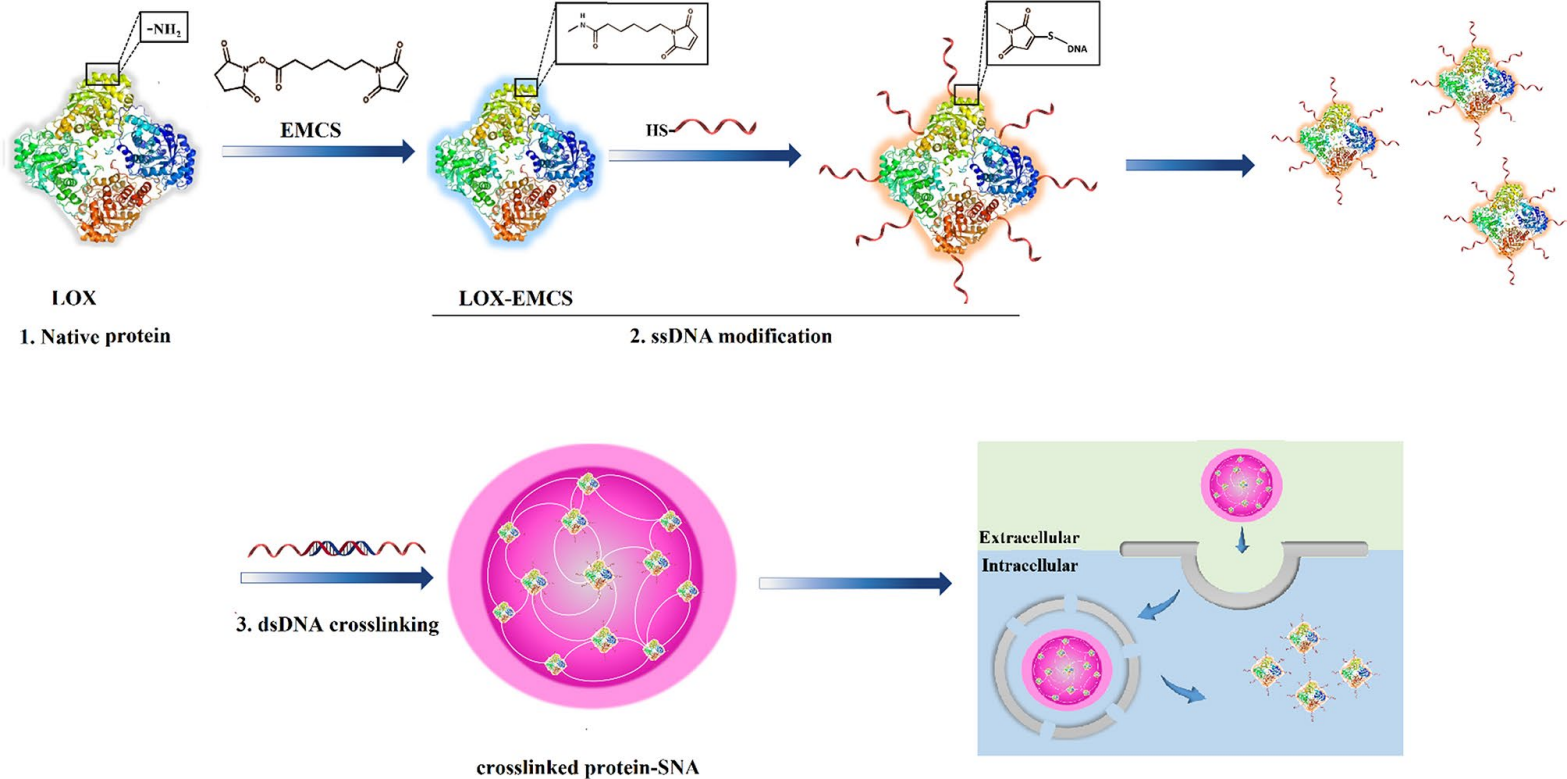
Scheme display of synthesizing crosslinked protein SNA (X-SNA). This figure was redrawn with permission from ref [61]

Effective intracellular protein-based therapies depend on cellular entry and resistance against degradation. For better therapeutic potential, particularly in the case of cell-impermeable proteins, protein-spherical nucleic acids (ProSNAs) are proposed as a class of systems, resulting in efficient uptake by cells [59]. ProSNAs are a form of metal-free delivery systems that are made up of a functional protein core and well-organized oligonucleotides shell [59]. ProSNAs have been established as a promising delivery nano-platform that makes possible the delivery of charged macromolecules with hydrophilic properties into cells and utilized as an intracellular sensor for live-cell analysis [59, 60]. The cellular uptake capability, maintaining enzymatic activity, blood circulation times, and accumulation of ProSNAs in major organs (e.g. lung, kidney, and spleen) can be adjusted by altering the conjugated ligand’s structure [59]. Yan et al. have developed a type of ProSNA system consisting of a lactate oxidase (LOX) enzyme core functionalized with an oligonucleotide shell [61]. In this strategy, individual DNA-modified enzymes are crosslinked with each other via a 36-bp dsDNA crosslinker containing oligo-T10 spacer and 3´ sticky end (30 bp), which is linked to complementary DNA strands on LOX and forms a structure, termed as crosslinked SNA (X-SNA) (Fig. 5) [61].

LOX X-SNAs showed higher delivery efficiency (up to 6 times) compared to enzyme-free and un-crosslinked ProSNA [61]. Also, its enhanced performance as an intracellular lactate probe has been reported compared to regular ProSNA (up to 3–4 times) [61]. The existence of crosslinker DNA converts LOX X-SNAs to a promising tool that can be used for targeting, cellular imaging, gene therapy, and immunomodulation [61]. Furthermore, despite the recent method for lactate detection, which is limited to the measurement of extracellular lactate and cell lysis, efficient delivery of exogenous LOX allows intracellular lactate measurement by quantifying the redox product (H2O2) [61].

#### DNA nanoclew (DC)

**Fig. 6:**
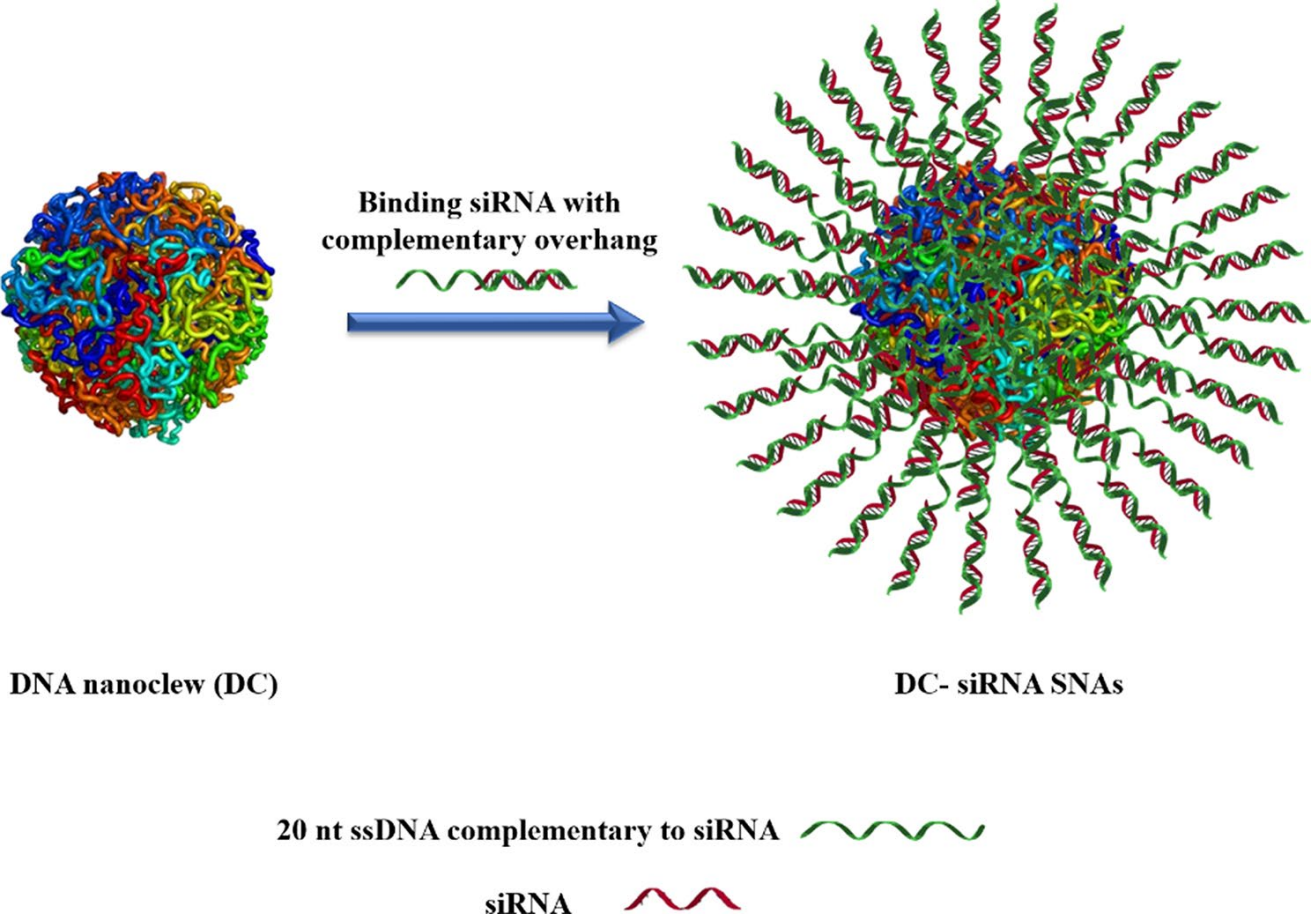
DNA nanoclew-siRNA formation by hybridizing siRNA with 20 nt ssDNA complementary overhang of DNA nanoclew. This figure was redrawn with permission from ref [1]

Deoxyribonucleic acid (DNA), a natural genetic macromolecule, can be utilized as a nanoscale carrier due to its tunable self-assembly, manageable properties (size, architecture, surface chemistry), and innate biocompatibility [1]. DNA nanoclew (DC) is a class of NPs based on DNA constructions that could be designed for carrying functional oligonucleotides or DNA-binding proteins (DBP) [1]. Ruan et al. introduced a new type of metal- or cation-free SNA, known as DC-siRNA [1, 33]. The DNA core of DC-siRNA NP was prepared through rolling-circle amplification (RCA) to engender a clew-like model [1, 33]. Subsequently, multiple copies of siRNA were assembled on the DC core by base-pair hybridization of the DC surface anchored with complementary overhang siRNA sequence of the linear template of the DC (Fig. 6) [1]. DC-siRNA SNAs exhibited efficiently targeted gene silencing at both microRNA and protein levels with inducing minimal cytotoxicity.

### Inorganic materials

#### Silver (Ag)

**Fig. 7:**
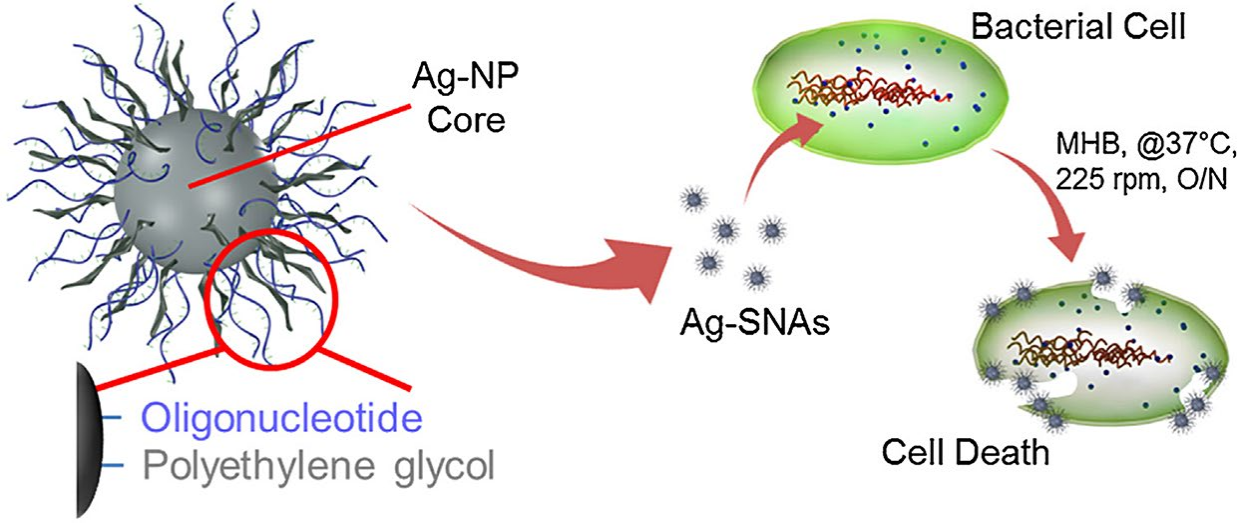
Illustration of the construct and interaction of silver spherical nucleic acids. Ag-SNAs from a silver nanoparticle core, functionalized with 3ʹ -thiol-oligonucleotide and methoxyl poly(ethylene glycol) thiol, and show potent antimicrobial features Permission was received from ref [66]

Silver nanoparticles (Ag-NPs) are one of the most extensively used nano-tools that have a dimension between 1 nm and 100 nm [62]. There is a wide range of preparation techniques for Ag-NPs synthesizing; the commonly used method is chemical reduction from silver nitrate (AgNo_3_) with different organic (Tri-ethyl-amine, Alpha-Terpineol) and inorganic compounds such as sodium citrate, sodium borohydride, ascorbate [63]. Also, the ‘Green’ synthesis of AgNPs (with plants, fungi, algae, etc.) has been used for its production [63]. Antimicrobial properties and simplicity in synthesizing, suitable and adjustable morphology, and high surface-area-to-volume ratio make nano-silver to be applied in the various fields of nanotechnology and biomedical. Moreover, AgNPs show higher plasmon excitation efficacy rather than AuNPs, which render them suitable to be used as biosensing and bioimaging tools and also in photo-controlled oligonucleotide delivery systems [64, 65]. Silver core spherical nucleic acids (Ag-SNAs) were described by Rische et al. as Ag-NPs with antibacterial properties against a large spectrum of both Gram-positive and Gram-negative organisms [66]. Ag-SNAs display significantly lower inhibitory concentration (MIC) compared to conventional Ag-NPs (30-fold) and lower cytotoxicity to mammalian cells (14-fold) (Fig. 7) [66].

#### Palladium (Pd)

Palladium nanoparticles (PdNPs), as nano-scale and metal-based nanocarriers, have been extensively employed in the catalysis of chemical reactions (hydrogenation and dehydrogenation) due to their unique antioxidant activities [67, 68]. More investigations have been done on using PdNPs in drug delivery systems or photothermal therapy for cancer treatment [69]. Among these, Fang et al. introduced a smart core-shell drug delivery technique based on mesoporous silicon-coated Pd@Ag nanoplates that provided simultaneous photothermal therapy and chemotherapy against tumor cells [49, 70]. PdNPs can not only deliver anticancer drugs but also can be functionalized with other small molecules such as proteins, DNA, and RNA to be exploited as antibacterial and antitumor therapies, imaging modalities, and targeted gene/drug delivery systems [49]. In this way, Kang et al. have shown that therapeutic oligonucleotide can be efficiently grafted on and released from porous palladium nanoplate [71]. Also, they expressed that porous Pd nanoplates could be an efficient delivery agent in biomedical treatment due to their excellent performance in photothermal conversion, cytotoxicity, and in vitro combination cancer therapy compared with Au and Ag nanoplates [71].

#### Platinum

In 1978, the FDA approved platinum-based drugs as anti-cancer agents [43, 72]. Nowadays, Platinum (Pt) drugs, including virtually 50% of all anti-tumor drugs, can be utilized alone or in accompany with other drugs for the treatment of various solid tumors, including colorectal, lung cancers, head and neck and ovarian carcinomas, and so on [43]. However, their side effects and toxicity (nephrotoxicity, ototoxicity, myelosuppression, neurotoxicity, cardiotoxicity, hepatotoxicity, anaphylaxis, alopecia, cytopenia, diarrhea, etc.) limit their applications [43, 73]. Nanobiotechnology provides diverse construction of Pt-based drugs to decrease their toxicity during blood circulation, increase delivery into the tumor site, and enhance drug uptake by tumor cells [43, 73], thus minimizing toxicity arising from Pt-based drugs and optimizing their efficiency [43, 73]. Among these, SNA as DNA-based nanoobjects have been used for anticancer drug delivery due to their low toxicity and intracellular stability [74]. Platinum (IV) is an alternative form of Pt (II) species with low side effects, higher reactivity and lower biological stability [74]. Loading Platinum (IV) onto SNA − Au NPs makes a potent anti-cancer drug delivery system [57]. Pt − SNA − AuNP complexes were successfully internalized into target cells [74]. These nanostructures are reduced into cytotoxic Pt (II) [57], entering the nucleus of the cell and cross-linking with genomic DNA [74]. Pt − SNA − AuNP platforms exhibited higher killing efficiency relative to cisplatin or the prodrug alone [74]. The inherent antimicrobial, antioxidant, and anticancer properties of platinum nanoparticles (PtNPs) make them extensively used in biomedical applications [75]. According to the results of platinum atomic absorption spectroscopy (AAS), 98% of the DNA amines on the SNA construction were conjugated to Pt [76]. Therefore, PtNPs could be embedded as the core of SNAs and become a platform for combinational gene/drug therapy, bioimaging, and bio-diagnosis [77].

#### Silica

**Fig. 8:**
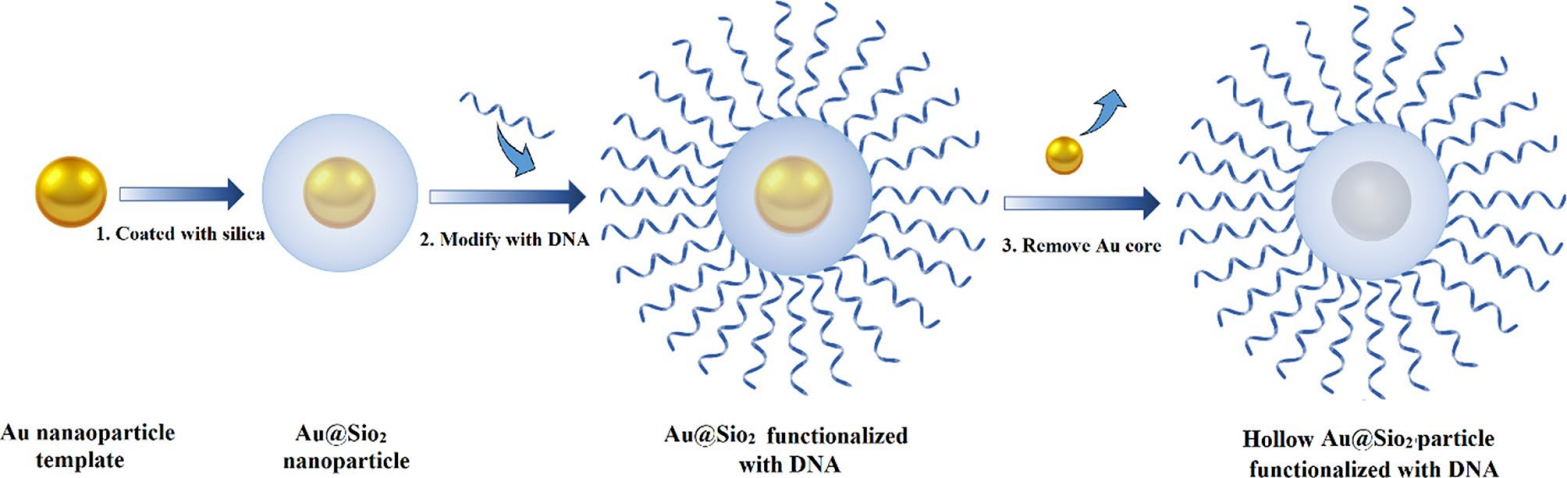
A sketch of DNA functionalized biocompatible hollow SiO2 synthesizing procedure using gold nanoparticles as sacrificial templates. This figure was redrawn with permission from ref [81]

Silica (silicon dioxide) nanoparticles (SiNPs) demonstrate a unique class of inorganic NPs, which are categorized in to non-porous SiNPs, mesoporous silica nanoparticles (MSN), hollow silica particles, and core(solid)-shell (porous) silica microspheres [78]. MSNs have hydrophilic surfaces and are covered with many empty pores (2- to 50-nm each pore size) [79, 80]. A wide array of beneficial features, such as high stability, scalable and easy synthesis, economical production, and biocompatibility, make them attractive for use in nano-medical applications [79]. Also, they can be undergoing surface modifications that elevate their potential for use as drug delivery systems, gene delivery carriers, and diagnostic sensors [78, 80]. One type of silica-based NPs for intracellular gene regulation is core-free SNAs. Young et al. developed a hollow SNA conjugate, in which gold NPs (as sacrificial templates) passivated with a short poly(ethylene glycol) (PEG) chain and coated with a thin biocompatible porous silica shell. Au@SiO2 NPs were functionalized with a dense layer of nucleic acids, and then the gold core was removed via oxidative dissolution (Fig. 8). These constructions successfully silenced the eGFP gene in endothelial cells of mice without cytotoxicity [81].

#### Quantum dots

Quantum dots (QDs) are spherical semiconductor particles on a nanometer scale (diameter of 2–10 nm) and exhibit unique spectroscopic characteristics and electronic properties. So, QDs are one of the interesting vectors for detecting drug/gene-guided therapy. QDot particles can be categorized based on their organization and structure in: (1) core type (comprising of a metallic chalcogenide such as PbS, CdTe, CdSe, etc.), (2) core-shell type (consisting of an inorganic core and an inorganic shell), and (3) alloyed semiconductor QDs (cadmium, selenium, telluride) [82, 83].

QDs can be created through physical, chemical, and biological methods [82]. However, hydrothermal technique and organometallic method are the most commonly applied approach for QD synthesis [82]. Among NPs, modified fluorescent QDs have been wildly investigated as potential gene delivery vectors in biomedical and nanomedicine applications due to their stable chemical properties and high loading capacity of oligonucleotides owing to their large surface area [84]. QDs also enable the evaluation of therapeutic efficiency via simultaneous tracking of transferred oligonucleotides circulation in cells in both in vitro and in vivo conditions [85]. Several studies have been conducted on the development of novel therapeutic approaches in the gene delivery field using QDs.

Lin et al. [86]. developed nanocarriers based on cadmium sulphoselenide/Zinc sulfide quantum dots (CdSSe/ZnS QDs) as an in vitro gene delivery system. CdSSe/ZnS QDs functionalized with siRNA targeting human telomerase reverse transcriptase (TERT). The results showed highly efficient siRNA transfection into two glioblastoma cell lines (U87 and U251) and significant suppression of TERT gene expression, which resulted in the suppression of the proliferation of tumor cells [86].

#### Iron oxide

**Fig. 9:**
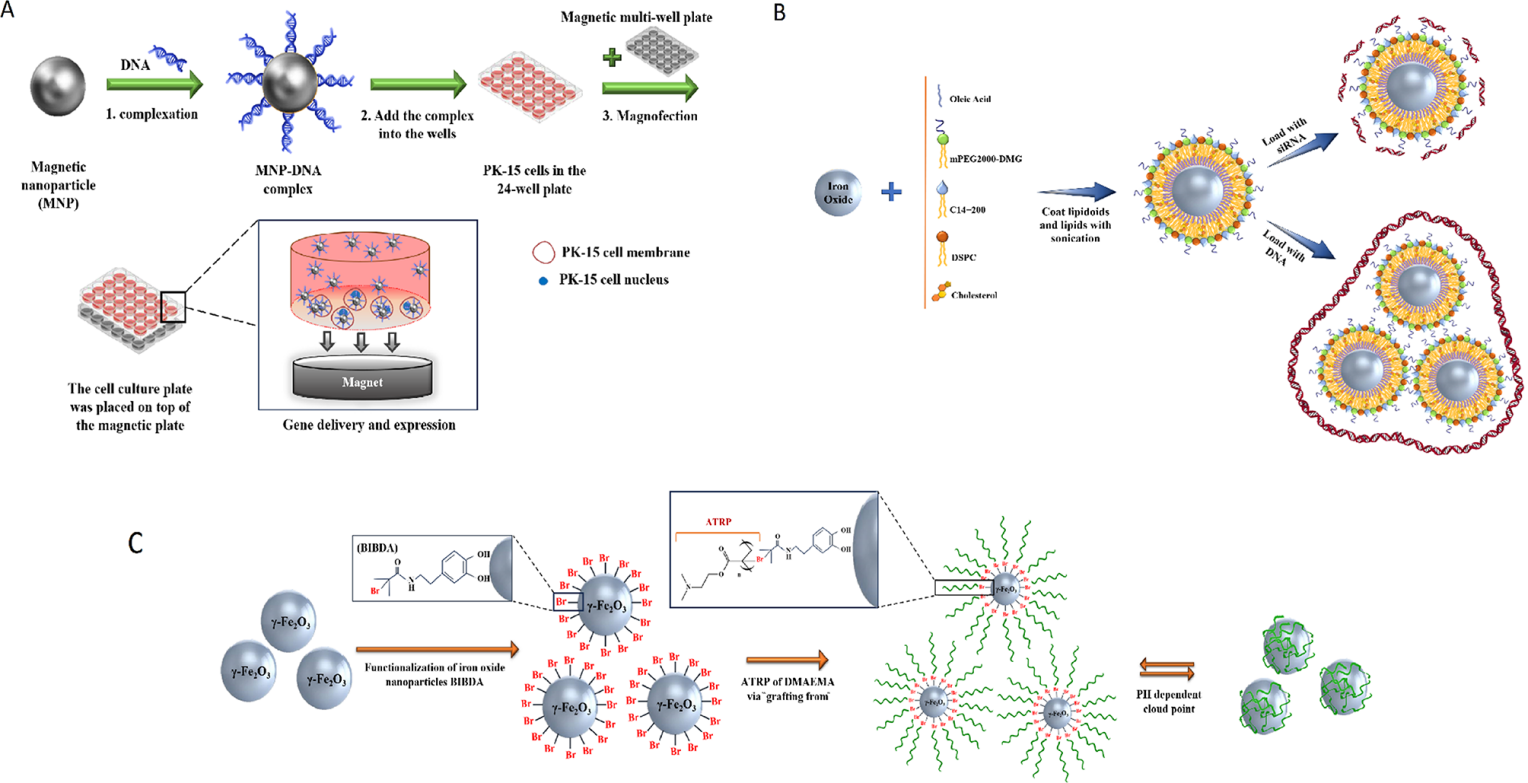
(**A**) Schematic drawing of the transfection procedure using magnetic nanoparticles under an external magnetic field [89]. (**B**) Design of the lipidoid-coated iron oxide nanoparticles coating procedure [90]. (**C**) Scheme illustration of the synthesis process of Dual-Responsive Maghemite Nanoparticles [93]. This figure redrawn with permission from mentioned references

Iron oxide-based nanoparticles, in particular maghemite (ɣFe_2_O_3_) and magnetite (Fe3O4), are representative of the magnetic nanoparticles (MNPs) [87]. Their biocompatibility and biodegradability attributes, and magnetic properties make them more attractive for use in numerous biomedical and pharmaceutical fields, such as diagnostics, drug/gene delivery, tissue repair, magnetic imaging, etc. [87–89]. Different types of synthesizing pathways were offered for Fe3O4 NPs production, including physical methods, wet chemical, and microbial methods [88]. The size of MNPs can be optimized for increasing DNA/siRNA delivery efficiency [90]. In fact, siRNA delivery illustrates similar efficiency in different sizes of NPs, while DNA delivery shows optimal efficacy with 50–100 nm NPs [90]. Several magnetic oligonucleotide delivery systems have been developed, in which coated NPs can carry DNA and siRNA into cells [90]. Wang et al. presented MNPs composed of an iron oxide core and polyethyleneimine (PEI) shell that was functionalized with an enhanced green fluorescent protein (EGFP) gene, which is expressed under external magnetic mediation [89]. The results showed that this form of MNP–DNA complex increased the entrance of NPs into mammalian cells [89](Fig. 9A).

Modified superparamagnetic iron oxide (SPIO), like Fe3O4 core with galactose (Gal) and polyethyleneimine (PEI) [Gal-PEI-SPIO], is another delivery system introduced by Yang et al. [1] for targeted delivery of therapeutic si-c-Met (siRNAs that target c-Met) to the hepatocellular carcinoma (HCC) [1]. c-Met is a receptor for hepatocyte growth factors and is overexpressed in most cancers, including gastric cancer, renal papillary cancer, and small-cell lung cancer [1]. Also, the c-Met gene is associated with the metastatic phenotype of cancer cells and poor prognosis [1]. The Gal-PEI-SPIO system demonstrated efficient absorption into Hepa1-6 cells and anticancer effects [1]. After injection, during systemic delivery in tumor-bearing mice, this delivery vehicle protected siRNA (si-c-Met) against serum nucleases and increased the accumulation of siRNA in orthotopic tumor tissues [1]. Also, evaluation of liver tissue showed a significant decrease in tumor volume and mRNA levels in the Gal-PEI-SPIO@si-c-Met group compared to the control group [1]. In another research that was accomplished by Kara et al. [91], PLL/Ser-SPIONs nanoplatforms were introduced as siRNA carriers [91]. In this system, SPIONs were covalently covered with a biocompatible protein, sericin (Ser), and altered with a cationic polymer, poly-L-lysine (PLL), which conferred a net positive charge to the particles to incorporate the negatively charged siRNA [91]. Control-siRNAs bind the PLL/Ser-SPION nanoplatform with a high binding efficiency (ranged between 81.90% and 93.50%) [91]. The results of cytotoxicity assays showed the biocompatibility of all formulations used in this nanoplatform against non-cancerous and cancerous cells [91]. The evaluation of the nano platform’s effect (PLL/Ser-SPIONs without siRNA) on clonogenicity showed that depending on the concentration of nanoparticles, the colony formation ability of cancer cells was maintained (note: except in high doses, cell clonogenicity was not blocked) [91].

Also, magnetic iron oxide NPs coated with a lipid-based agent (lipidoids) have been developed by Jiang et al. for guided targeting via magnetic force, gene therapy, imaging simultaneously, and magnetic thermotherapy (Fig. 9B) [90]. In this nanostructure, iron oxide nanoparticles were covered with lipids and lipid-like molecules by sonication under nitrogen protection. Then siRNA and DNA were grafted onto the surface of lipid-coated iron oxide nanoparticles by electrostatic interaction with the cationic lipid layer (for DNA transfection, 1 DNA molecule were bound to ∼3 nanoparticles, and for the siRNA transfection, ∼100 siRNA molecules were bound onto each nanoparticle). Lipidoid-coated iron oxide NPs have simplistic synthesis without purification stages, and more capacity for DNA and siRNA loading onto magnetic NPs compared to direct joining methods [90]. Furthermore, in undersize controlling conditions (optimal size [50–200 nm] for tumor targeting), this approach can be useful for in vivo applications due to the increased permeability and maintenance effect [90].

The next method provided by Majewski et al. is known as dual responsive MNP (γ-Fe_2_O_3_@PDMAEMA). It is prepared from the γ-Fe_2_O_3_ core and a water-soluble cationic polymer (pDMAEMA), which assembles on the core surface through dopamine initiator (2-bromoisobutyryl dopamide (BIBDA)) and 2-(dimethylamino)ethyl methacrylate (DMAEMA) [92, 93](Fig. 9C).

PDMAEMA can bind DNA with electrostatic interaction and create dense complexes [92]. The nature of the core of this system confers magnetic properties. The present approach showed adequate stability in watery media in a wide range of pH and can agglomerate reversely in pH- and temperature-dependent manner [93]. Further, this hybrid system offers high transfection capacity [almost 2 times higher compared to polyethyleneimine (PEI)] and low in vitro cytotoxicity [93]. Additionally, they do not depend on the usage of a magnetic field in contrast to PEI- MNPs systems, which most of them needed the application of a magnetic force for gene delivery (Magnetofection) [93].

#### Buckminster C_60_-fullerene core

**Fig. 10:**
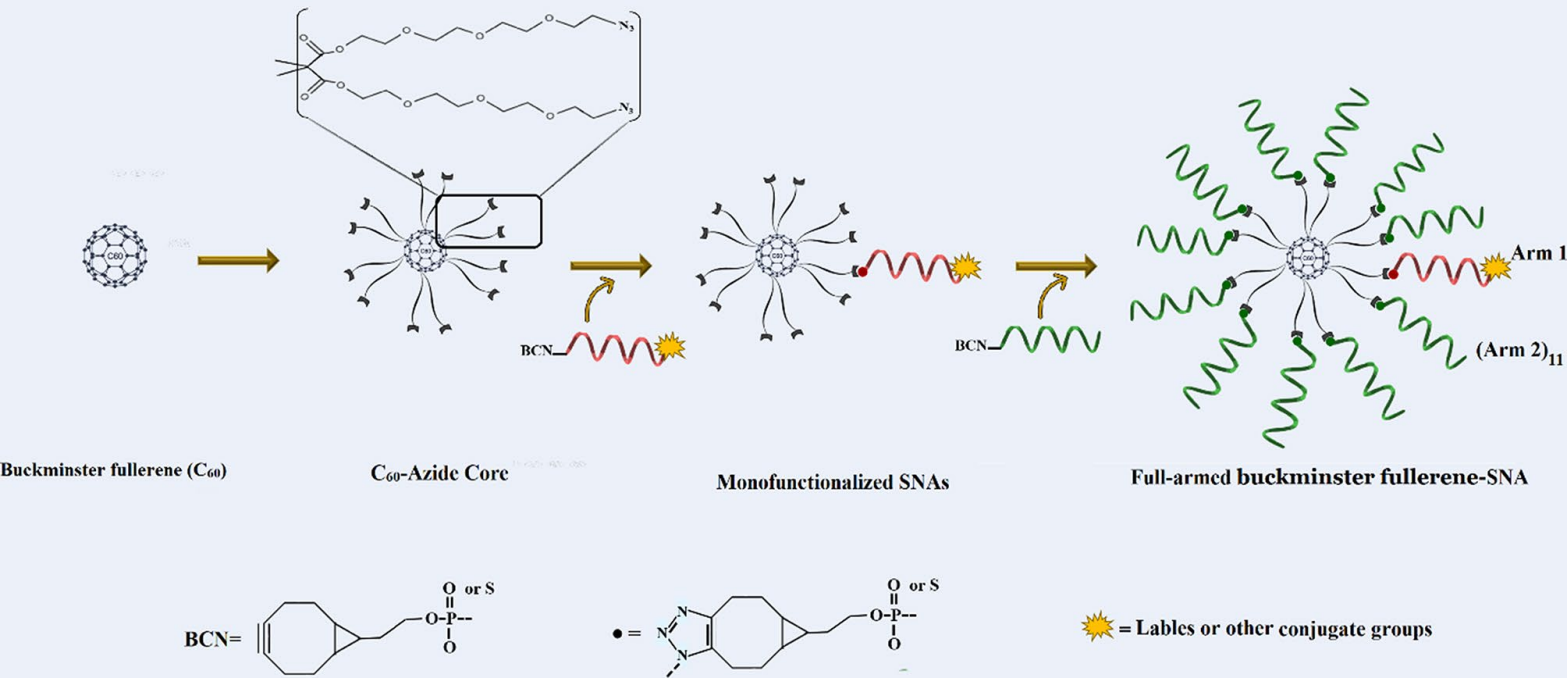
Illustration of the synthesis processes of C60-based SNAs. This figure was redrawn with permission from ref [95]

The fullerene C60 is a carbon-based spherical nanostructure, in which 60 carbon atoms are kept together by sp2 hybridization [94]. Gulumkar et al. [95] introduced C60-based SNAs made up of a C60-azide scaffold with 12-armed Buckminster fullerene and cyclooctyne-modified oligonucleotide strands. One of 12 cyclooctyne-modified arms was labeled with DOTA [1,4,7,10-tetraazacyclododecane1,4,7,10-tetraacetic acid] and Alexa 488 for the monitoring of cellular uptake and biodistribution of these decorated SNAs (Fig. 10).

In this structure, the sufficient concentration of oligonucleotides on the structure made possible internalization through scavenger receptors. It has been shown that they could internalize into breast cancer (MCF7) cells with ∼ 500-fold higher potency relative to free oligonucleotides. Also, C60-based SNAs were able to regulate their target [human epidermal growth factor receptor 2 (HER2) mRNA] [95].

#### Ribozyme − spherical nucleic acids

**Fig. 11:**
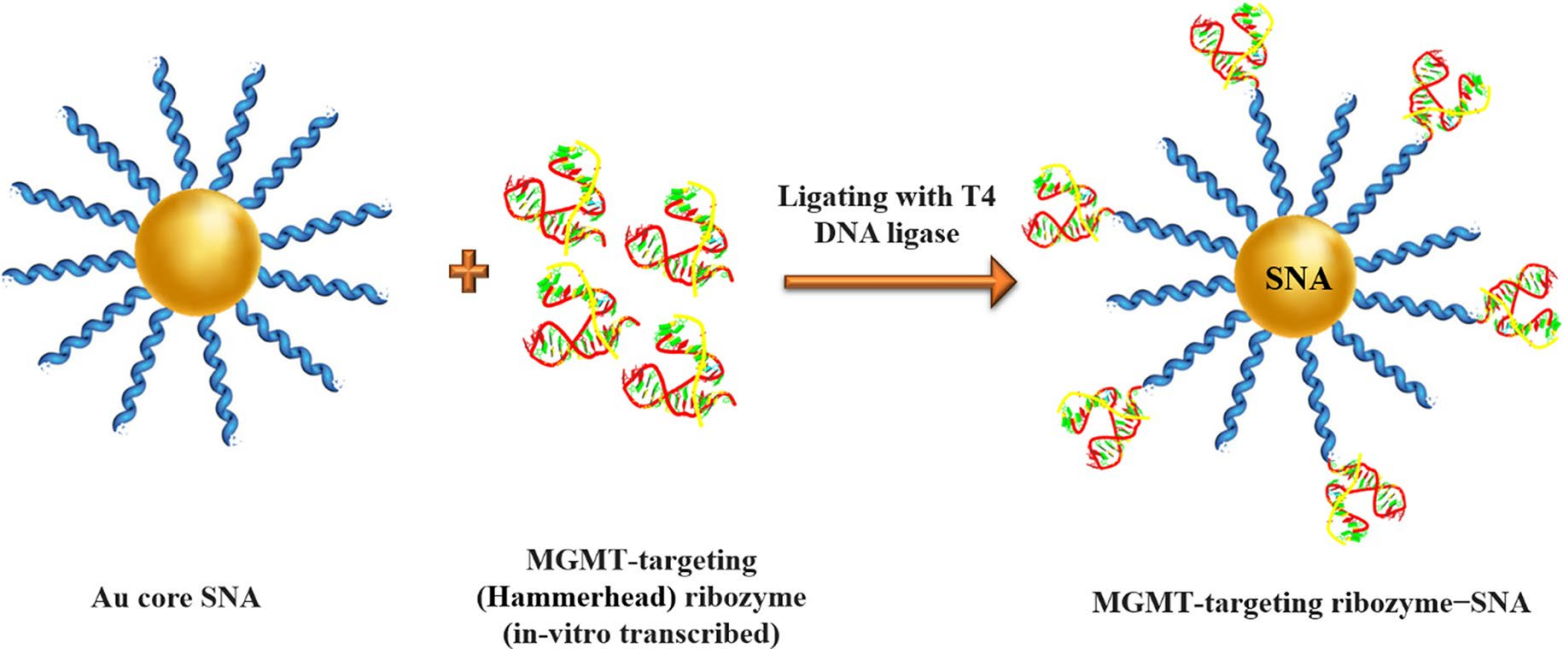
Illustration of MGMT-targeting ribozyme-SNA formation via enzymatic ligation of MGMT-targeting (hammerhead) ribozyme to B-form DNA at the surface of gold nanoparticles. This figure was redrawn with permission from ref [36]

Ribozymes are RNA molecules have a well-defined tertiary structure, which confers them high catalytic activity [96]. The term of ribozyme represents the ribonucleic acid and enzymatic activity nature simultaneously [96]. They act like enzymatic proteins and are involved in the catalysis of metabolic and chemical reactions within the cell [96]. Natural ribozymes are classified into the hammerhead, hairpin, Varkud Satellite (VS), Hepatitis delta virus (HDV), glucosamine-6-phosphate riboswitch (glmS), the group I and II introns, ribosome, RNase P, and spliceosome [96]. The expression suppression of specific mRNA can be accomplished by ribozymes [97]. Ribozymes are capable of specifically binding in a Watson-Crick pairing and making cleavage within a target mRNA substrate. Despite antisense oligonucleotides, ribozymes deactivate the target complementary RNA Independently of host cell machines and they can also break more than one copy of the target RNA by releasing from the cleaved product and attaching to another target molecule [97, 98]. So, in a study, Rouge et al [36]. developed the ribozyme − SNA architecture for targeting O6 -methylguanine-DNA methyltransferase (MGMT), which is a DNA repair protein enzyme associated with chemotherapeutic resistance (temozolomide [TMZ] resistance) of solid tumors and principally glioblastoma multiforme (GBM). Ribozyme − SNA constructed by truncated MGMT-targeting ribozymes, containing a hammerhead-type structure ligated to B-form DNA at the surface of a divalent ion (gold) enzymatically (Fig. 11). Invitro experiments showed that MGMT-targeting ribozyme − SNAs were efficiently taken into T98G glioma cells, maintained their stability (enzymatic activity), successfully knocked down their target (75% knockdown without transfection agents), and finally sensitized cells to TMZ-mediated apoptosis [36].

#### Coordination polymers

**Fig. 12:**
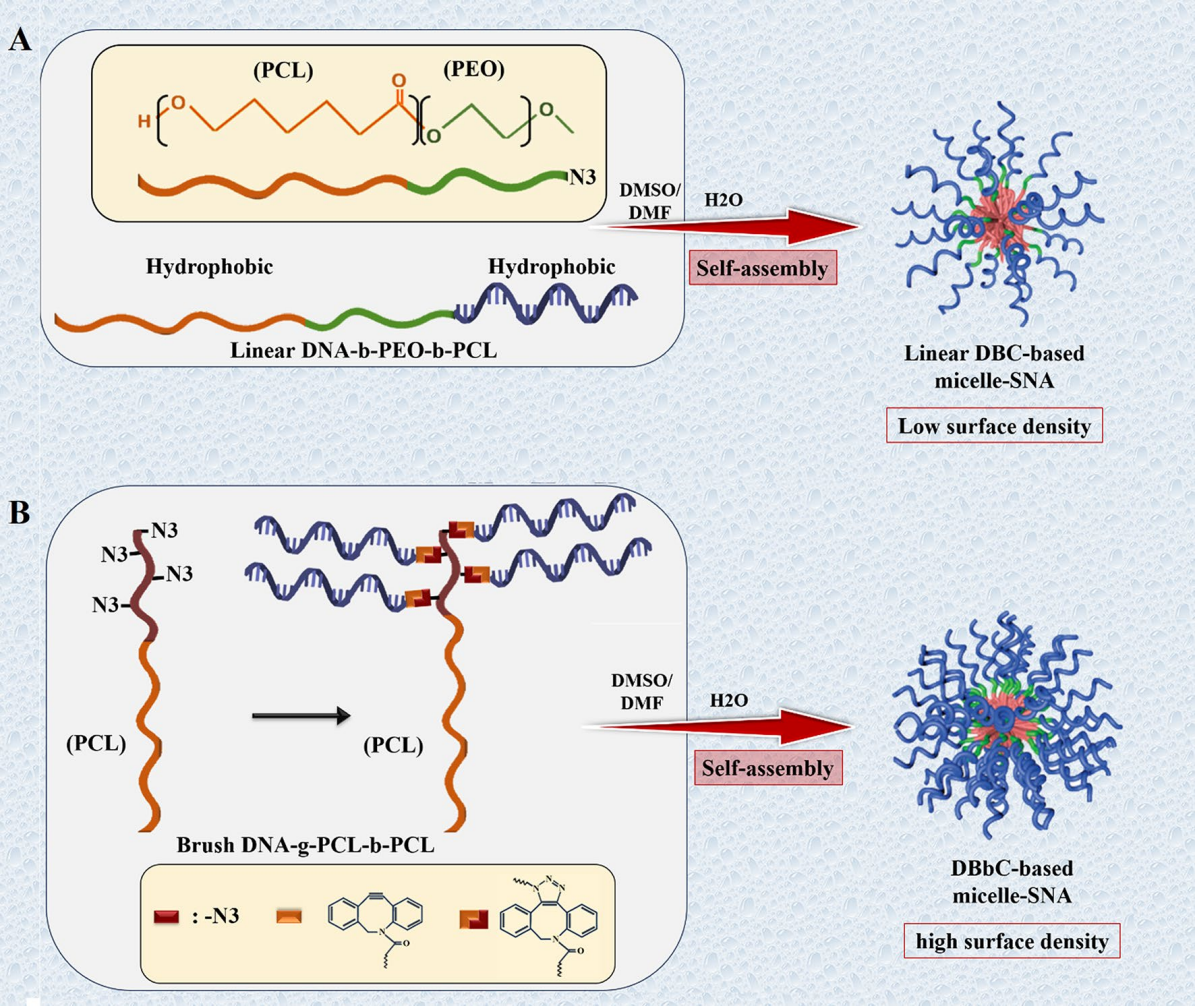
Schematic model for synthesizing DNA conjugated block copolymer-based micelle-SNAs. (**A**) The preparation of the linear DNA- b -PEO- b -PCL block copolymer and the related construction of micelle-SNAs (LDBC-SNAs). (**B**) The preparation of the brush DNA- g -PCL- b -PCL block copolymer and the corresponding formation of micelle-SNAs (DBBC-SNAs) consisting of a higher surface density of oligonucleotides. This figure was redrawn with permission from ref [99]

Biodegradable DNA-Brush Block Copolymer (DBBC) is based on micelle-SNA structures, introduced by Zhang et al. [99]. DBBC macromolecule was prepared by attaching numerous DNA strands onto the terminal fragment of a di-block copolymer comprising of polycaprolactone (PCL) and azide-modified PCL through copper-free click chemistry (Fig. 12) [99].

These structures showed increased oligonucleotide loading capacity, a higher melting temperature, and more efficient cellular entry compared to DNA block copolymer (DBC)-based micelle structures [99]. The research results also indicated that DBBC could knock down target genes efficiently in vitro [99]. Moreover, DBBC can be used as a programmable oligonucleotide releasing system into cell due to the nature of the polymeric core, regularly degraded with ester cleavage arising from esterase enzyme activity or via acid-catalyzed reaction [99].

Different types of polyesters, such as polylactic acid (PLA) and poly (lactic- co -glycolic acid) (PLGA), can be employed as polymeric cores of SNAs [99]. Poly (lactic-co-glycolic acid) (PLGA, PLG) is a synthetic biodegradable copolymer that is produced from polylactic acid (PLA) and polyglycolic acid (PGA) [100]. PLGA as a biocompatible nanomaterial can be easily subjected to hydrolysis and broken down into its monomers in vivo, which are metabolized in the tricarboxylic acid cycle for degradation in the lungs [100]. Hence, it has been determined as a biomaterial used in delivery systems of drugs, DNA, RNA, proteins, and peptides [100]. Also, PLGA becomes an appealing material for constructing the core of SNAs [101]. In a strategy reported by Zhu et al., PLGA-SNAs, consisting of PLGA cores (50 nm diameter) ended in azides and nucleic acid strands terminated with the dibenzocyclooctyne (DBCO) group, were successfully synthesized employing copper-free click chemistry [101].

## SNAs optimizing mechanisms

After introducing SNAs into serum, serum proteins bind to the surface of SNAs and create a protein corona [47]. SNAs are internalized into the cell by the engagement of.

surface scavenger receptors, inducing endocytosis and the formation of late endosomes [21]. Then endo-/lyso-somal escape should occur efficiently so that SNAs reach their target site in the cell [21]. A variety of protein coronas (identity, number, and types of bonded proteins) can form on SNAs depending on their design [33]. Thereby, the density, identity, and sequence of oligonucleotides can be adjusted to modify the 3D architecture of SNAs, affecting the properties of SNAs, especially the cellular uptake and in vivo biodistribution [102]. Notwithstanding the considerable properties of SNAs, mainly arising from the dense array of oligonucleotide strands, the substitution of different types of NP core can influence catalytic and optical activity, as well as the biodegradability of SNAs [102]. So, utilizing SNA in the clinical field requires spacious modifications of the sequence of oligonucleotides and the selection of suitable core NPs to ensure efficient delivery, removal from the bloodstream, passing through biological barriers, and effective internalization into the target cell [103].

### SNA biostability

Stability is one of the necessary features for any delivery system to be used as a diagnostic or therapeutic tool [104]. Normally, nanoparticle stability is defined by a spectrum of physicochemical properties of NPs including morphology, size, core composition, aggregation, and surface chemistry [105]. All these lead to an exclusive aggregation profile of diverse plasma proteins, which have a direct relationship with the length of maintenance in blood circulation [43].

#### Enhancement of blood circulation time

The therapeutic benefits of NPs rely on their capability to remain in the bloodstream long enough to reach their therapeutic position. By introducing the therapeutic nanoparticles (NPs) into the systemic circulation, the reticuloendothelial system (RES), also named as mononuclear phagocytic system (MPS), quickly intervenes and removes most of the nanoparticles. RES directs the clearance of NPs through aggregation, destabilization, opsonization, and hepatic and renal clearance. So, this leads to their shortened half-life and narrowed accumulation at the therapeutic site and limits their applications. Previous studies indicated a negligible immune response of SNA (25-fold lesser) compared to conventional oligonucleotide transfection methods like cationic nanocarriers (lipoplex). However, several chemical (e.g., PEG) and physical (e.g., size) modifications of SNA were reported to improve blood circulation and circumvent the phagocytic cells of RES. As Poly (ethylene glycol) (PEG) functionalization of SNA is used to stealth against serum protein adsorption and formation of the protein corona, which makes the nanoparticle detectable to the immune system. So, PEGylation can increase circulation time.

#### Enhancement of resistance to serum nucleases

**Fig. 13:**
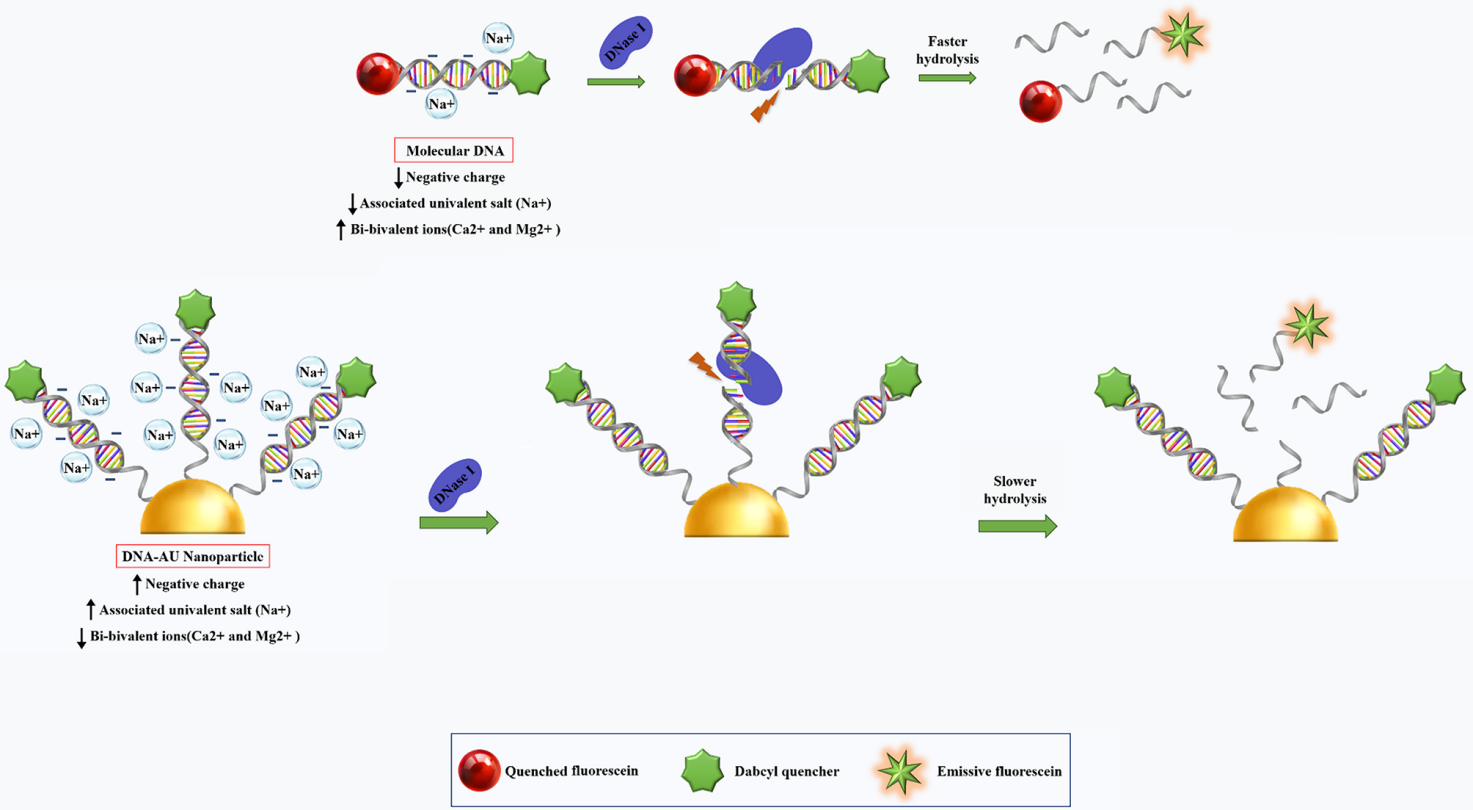
Recommended procedure for increasing the DNA stability of polyvalent nanoparticles [104]

During systemic delivery, nucleic acids are prone to degradation by serum nucleases that lead to significant losses in their activity and functionality. In general, SNAs show better nuclease stability than linear oligonucleotides due to their 3D constructs and highly oriented arrangement of oligonucleotide sequences on their own surface. Nuclease-catalyzed DNA hydrolyses can occur in two steps: (1) binding of the enzyme to the substrate, and (2) hydrolyzing oligonucleotides [106]. DNA-Au NPs stability can be achieved by reducing the rate of either of these steps [104]. NPs properties, including surface density and charge of oligonucleotides, are the key factors associated with the increased nuclease resistance of DNA-Au NPs [104]. The high surface density of DNA increases resistance to nuclease-driven degradation that may be due to the effect of steric inhibition or interaction with local salts [104]; however, steric hindrance is not the major cause of their stability [104]. DNA-Au NPs have higher melting temperatures and high binding constants (affinity constant/association constant) [104, 107, 108]. So, densely arranged DNA/RNA oligonucleotides contain more negative charge that could be associated with local salt (Na+) [104]. Formerly, it has been established that univalent cations (e.g., Na+) prevent the activity of DNase I and related nucleases by dislocating necessary ions (e.g., Ca2 + and Mg2+) for enzyme activity (Fig. 13) [104]. These results confirmed that salt association with DNA-Au NP is the main factor contributing to the inhibition of enzyme activity and thus the stability of DNA-Au nanostructure [104]. Indeed, DNA-Au construction does not inhibit enzyme binding to its substrate but rather prevents enzyme-catalyzed degradation [104]. As a result, DNA-Au NP have a greater half-life (4.3fold) than molecular DNA systems [104].

In addition to DNA oligonucleotides, RNA oligonucleotides can also be loaded on the core of SNAs. DNA oligonucleotides can be loaded onto SNA cores at approximately 60–80 strands per core, while RNA has been loaded onto cores at almost 30–45 RNA strands per core [41]. RNA-SNAs, compared to DNA-SNAs, have lower stability [41]. Therefore, the suggested strategies to achieve highly stable RNA-SNAs synthesis consist of (1) coating SNAs with thiol-polyethylene glycol (PEG); however, the results showed that increasing PEG density on the surface of RNA-SNAs decreased cellular uptake [41]. (2) Using altered oligonucleotides (i.e., 2′-O-methyl and phosphorothioate oligonucleotides) instead of the common oligonucleotides is another strategy [41, 109]. In oligonucleotides with phosphorothioate bonds, the non-bridging oxygen atom in the phosphate backbone of a (deoxy) ribonucleic acid is replaced with a sulfur (S) atom that makes them more stable against both exo- and endo-nucleases [41, 109].

Additionally, Barnaby et al. introduced RNA spherical nucleic acids (RNA-SNAs), consisting of dense thiol-modified RNA duplex (siRNA) shells on AuNP surfaces. Four design parameters involving in SNA nuclease stability, including sequence, spacer, density, and backfilling, were evaluated. Experiments have shown that the presence of sequences containing motifs recognized by RNases (e.g., UA/AU) close to the surface of the NP core causes rapid serum nucleases-mediated degradation, despite their dense arrangement. Also, the possible importance of the nature and length of the spacer, the region between the propyl thiol group, and the RNA identification sequence, in the rate of nuclease-catalyzed hydrolysis was suggested. It has been shown that poly T DNA spacer (a stiff DNA spacer) slows down the rate of nuclease-catalyzed degradation compared to hexaethylene glycol spacers, where oligonucleotides are driven away from the NP surface due to the charge of RNA molecules and spacer flexibility [44]. It was also illustrated that unlike DNA-SNAs structures, where the density of duplexes at the nanoparticle level does not affect the rate of nuclease-mediated degradation, increasing the number of duplexes in RNA-SNAs enhances their stability [44, 104]. Even after completely loading the NP with RNA, there is still unloaded space that can be filled by more neutral backfill molecules such as PEG or other types of backfill molecules [44]. Furthermore, research indicated that backfilling decreased enzymatic access and increased the half-life of RNA-SNAs, reflecting its importance in ribonuclease (RNase) resistance [44].

### SNA biodistribution

Biodistribution and pharmacokinetics of NPs are other critical factors for systemic applications [110]. To a considerable extent, biodistribution influences the NP’s diagnostic and therapeutic efficiency, biocompatibility, and toxicity. Lately, evaluations indicated the non-significant (< 1%) accumulation of NPs within the tumor site after intravenous administration [21, 111]. However, the factors influencing the biodistribution of SNAs are not completely clear [112]. Toward this, finding the physicochemical properties of SNAs (e.g., surface chemistry, charge, shape, size), which will affect their extent and specificity of interactions with serum proteins, will lead to developing a precise delivery systems and boosting their clinical utility [21].

In general, the size of NPs affects NPs´ interactions with the transportation and defense systems of the body and cells, which in turn alter their biodistributions and accumulation in the body. In vivo investigations revealed that in systemic administration, an NP with $<10$ nm size range could distribute quickly among various organs and tissues. However, most NPs with the 50–250 nm size rang are largely recognized by specific immune systems and absorbed by the mononuclear phagocytes. So, they are specifically seen in the blood, liver, and spleen [113].

**Fig. 14:**
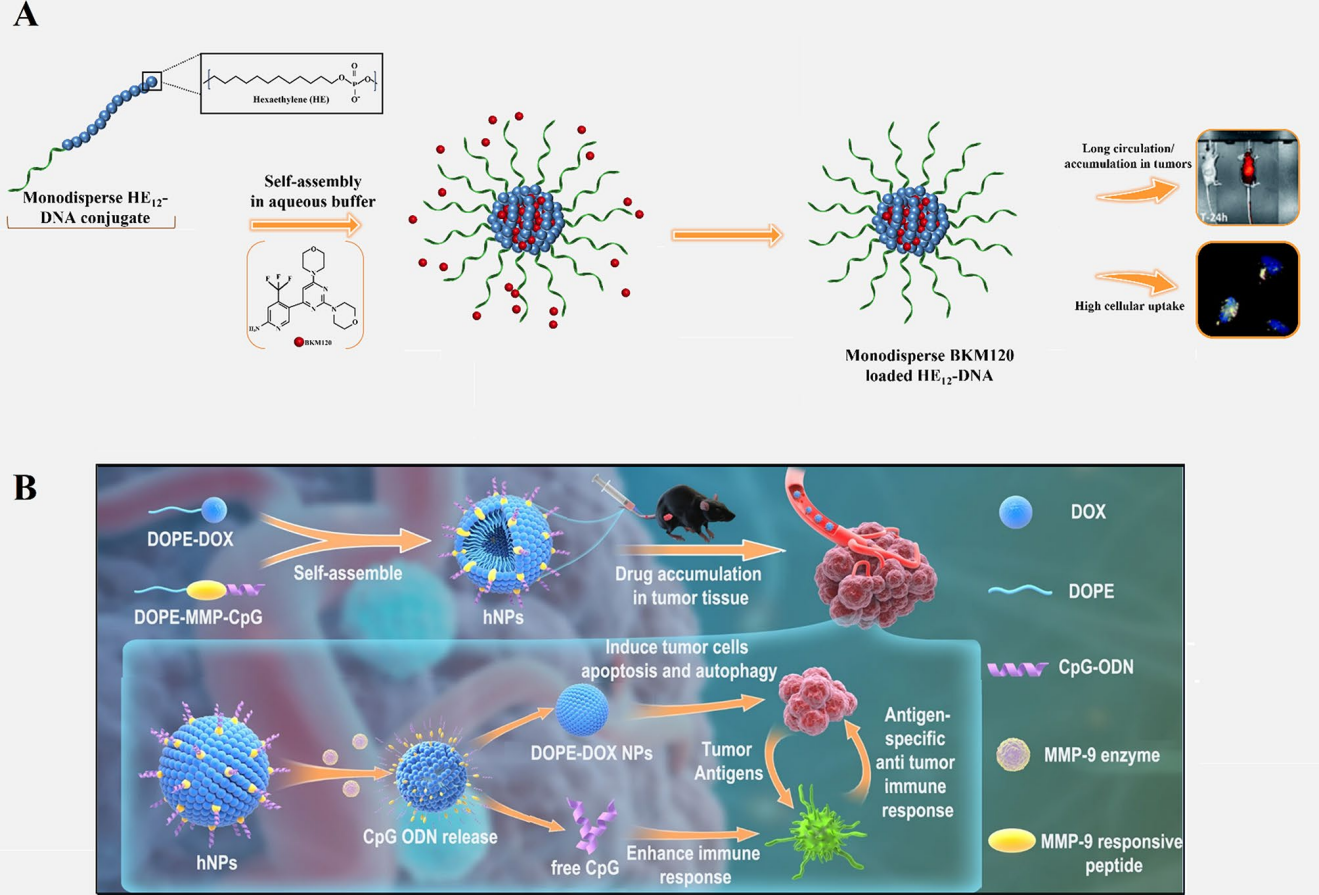
(**A**) Schematic display of the method for synthesizing DNA-polymer conjugates with BKM120 encapsulated cargo, microscopic images of the cellular uptake in HeLa cells after 24-hour incubation (show high cellular uptake), and image of Cy5.5 fluorescence intensity at the tumor site 24 h after subcutaneous injection (showing long circulation and accumulation in tumors) [116]. (**B**) Schematic representation of DOX and CpG-loaded liposomal SNA (L-SNA) and its mechanism of function [118]. Permission was received from the mentioned references

Nanoparticles can be programmed for targeted delivery, controlled release, and accumulation of drugs and therapeutic oligonucleotides in specific organs and tissues [114, 115]. Some studies have examined the effect of structural changes in the presentation of predetermined corona protein compounds on the biodistribution cargoes and their targeted delivery. In addition, new designs of SNAs with different formulations have been proposed for the co-delivery of drug and therapeutic oligonucleotides to the tumor site for chemotherapy. Bousmail et al. [116] developed an SNA system comprised of micellar NP core and shell of DNA sequence (19 nt) bonded to 12 dodecanes (hexaethylene, HE) elements (HE_12_–DNA). This nanoconjugate was designed for anticancer drug (BKM120) delivery intended for chronic lymphocytic leukemia (CLL) therapy. Encapsulation of BKM120 in the SNA construction largely prevented its adverse effects. In vivo, this drug delivery system indicated long blood circulation times (up to 24 h), whole body distribution (but to a lesser extent in the brain, lungs, kidneys, and liver), high accumulation at tumor sites, and partial penetration through the blood-brain barrier (Fig. 14A). Additionally, HE12–SNAs showed stability and efficient uptake by cells. All of these, caused them to be considered promising delivery nanoplatforms for chemotherapeutics [116].

In another study, Deng et al. [117] presented a liposome-based SNA construction consisting of lipid-drug (1,2-dioleoyl-sn-glycero-3-phosphoethanolamine [DOPE]-doxorubicin) and lipid-DNA (DOPE-matrix metalloproteinases-9 [MMP-9] responsive peptide-CpG) conjugates. CpG oligodeoxynucleotides (CpG ODN) offer immunostimulatory agents that elevate the penetration of immune cells into tumor microenvironments (TEM) by attaching to Toll-like receptor 9 (TLR-9) in the endosome. DOX could induce immunogenic cell death (ICD) of tumor cells to release tumor-specific antigens (TSA). DOX and CpG release via MMP-9 enzyme in TME could increase tumor-specific antigen (TSA) releasing and recruitment of CD8 + and CD4 + T cells to both tumor environments and spleen, repressing tumor development, and improving animal survival (Fig. 14B). Therefore, the LSNA- DOX-CPG system could enhance the direct killing effect of lipid-drug (DOX) on tumor cells and boost potent tumor-specific immune responses by CPG reagent to achieve a synergistic therapeutic effect, while diminishing systemic toxicity [117].

Providing chemical structure to the liposomal SNAs makes it possible to conduct nanocarrier to main tissues outside of the reticuloendothelial system and targeted therapeutics [22]. Jennifer R. Ferrer et al. [22] indicated that functionalization L-SNA with ssDNA containing a low-affinity cholesterol (hydrophobic) tail (CHOL-LSNA) greatly tends to accumulate in the lungs, whereas L-SNA with ssDNA containing a high-affinity diacylglycerol lipid tail (DPPE-LSNA) largely accumulation in the kidneys. While both LSNA architectures showed reduced inflammatory responses in systemic administration [22].

Immobilization of active protein coronae on surfaces of SNA is another study on improving the targeting and biodistribution of SNAs in vivo, which was reported by Wuliang Zhang et al. [46] It has been shown that incorporating anti-Her2, IGg immunogenic protein, and human serum albumin (HSA) with SNA confers cell targeting, and biodistribution capability and also improves SNA stability. In this system, anti-Her2 was used as a model protein for HER2-positive breast cancer cell targeting. IGg and HSA lead to alteration in the major cellular uptake pathway of SNAs and an increase in blood circulation half-life by protection against the reticuloendothelial system, respectively [46]. Functionalization the surfaces of SNA with aptamer sequences as a nucleic acid shell is another improving mechanism for targeting and biodistribution of SNAs. In a study, Caroline D. et al. reported that functionalization protein(β-galactosidase)-SNA with DNA aptamers binding to transferrin (TfR) receptors, expressing on the endothelial cells improves the internalization and accumulation of Transferrin- functionalized- ProSNAs after intravenous injection in brain tissue compared the native protein and non-functionalized-ProSNAs. Therefore, it was suggested that selective delivery of cargo can be reached by designing appropriate aptamer sequence as an oligonucleotide shell [119].

### Enhancement of SNA internalization capability

#### Sequence optimizing

Investigations highlight the importance of oligonucleotide sequences in the formation of SNA’s protein corona components [47]. The sequence of oligonucleotides with high G content maximizes internalization independently of core NPs, indicating that G-rich SNAs have more (4–10-fold) affinity for cell surface receptors (SR-A) compared to SNAs composing of poly A, poly T, and poly C. G-rich SNAs can be used to increase the delivery of both oligonucleotide and small molecule drugs into cells [103]. However, studies revealed that G-rich sequences lead to dense coronas production, which in turn induces macrophage activation and potent immune responses compared to T-rich sequences, which generate quite thin coronas [47]. This reveals that the sequence composition of SNA has a fundamental role in directing biological interactions. Modifying the sequence enables the rational design of SNA constructs with desired in vivo fate and cellular uptake properties.

#### (Poly)ethylen glycolation

(Poly)ethylen glycol (PEG) is a petroleum-derived polymer [120]. Its hydrophilicity and biocompatibility nature make it to be used to increase the blood circulation of therapeutic agents [120]. In the case of RNA − SNAs, PEG is the other component of SNAs beside the NP core and oligonucleotide shell, interspersed within the shell [44]. PEGylation creates a steric barrier against protein adsorption and formation of the protein corona, thereby blocking interactions with the cellular internalization receptors (e.g. scavenger A receptors), decreasing cellular uptake, and increasing blood circulation time relative to PEG-free SNAs [21]. Longer circulation time in vivo provides adequate time for reaching the target site [44]. Therefore, identifying the optimal extent of PEG content depends on the specific application and understanding the presence of an inverse relationship between circulation time and cellular uptake [44]. A SNA system used for topical application (e.g. skin) needs lower PEG content than those used for intravenous delivery [44]. Numerous investigations affirmed the impact of nanoparticle PEGylation on the structural features and biological activities of SNAs. These findings and future studies will enable the improvement of SNAs, which effectively reach disease sites and enhance therapeutic efficacy.

#### Sugar backbone

Variations in the sugar-phosphate backbone of the oligonucleotide shell affect different characteristics of SNAs, including lipophilicity, surface chemistry, and hydrogen bonding interactions of SNAs; all these alterations may have impact on the internalization of SNAs [121]. In a recent study, the effects of five types of sugar backbones (DNA, L-DNA, RNA, 2´ -methoxy-RNA [2´ -OMe-RNA], and 2´ -fluoroRNA [2´ -F-RNA]) were evaluated on the rate of cellular uptake of SNA [121]. The results showed that the variations of the sugar-phosphate backbone altered the internalization mechanisms of SNAs; however, internalization by the scavenger receptor pathway was predominant [121]. Among the mentioned backbones, 2´-F-RNA showed increased lipophilicity and the highest uptake rate compared to others [SNA2_´ −F−RNA_ (222%), SNA_RNA_ (147%), SNA_DNA_ (100%), SNA_L−DNA_ (60%), SNA _2´− OMe−RNA_ (41%)] [121].

### Enhancement of surface loading capacity

The dense assembly of nucleic acids, as well as the type of NP core, plays a fundamental role in determining SNAs´ properties and nanomaterials’ fate (delivery, elimination, tissue accumulation, etc.) [33, 122]. It was previously shown that thiolated oligonucleotides could attach to the surface of gold NPs due to the high affinity of thiol parts to AuNPs [123]. Furthermore, it has been confirmed that the surface loading of oligonucleotide strands per NP is elevated by increasing salt (NaCl) concentration [123]. However, the dense arrangement of nucleic acids on the surface of NPs core is affected by the size and shape of the NPs core [33]. Small-sized NPs (< 20 nm) show a remarkable increase in surface loading capacity compared to larger ones mainly due to the higher radius of curvature of smaller NPs, which provides a natural deflection angle between neighboring oligonucleotide strands and the extra space around each strand [123]. Also, a sphere-shaped gold nanoparticle with 10 nm in diameter supports the loading of ∼ 2.0 × 10^13^ oligonucleotide strands per cm^2^, while in the same condition, 5.8 × 10^12^ oligonucleotides per cm^2^ can be loaded on the surface of flat counterparts [33, 123]. In addition, evidence has shown that when oligonucleotide sequences are uniform, the type of purine or pyrimidine bases is also effective in loading capacity. Pyrimidine-rich oligonucleotides (e.g., poly T [≈ 180 ssDNAs/AuNP] and poly C [≈ 140 ssDNAs/AuNP]) show higher surface loading than purine-rich oligonucleotides (e.g., poly G [≈ 75 ssDNAs/ AuNP] and poly A [≈ 45 ssDNAs/ AuNP). On the other hand, non-uniform oligonucleotide sequences do not affect the kinetics of cellular uptake. G-rich SNAs show higher internalization than T-rich SNAs despite their lower loading on the surface of NPs [124]. Therefore, the surface loading capacity is one of the critical properties of SNAs because of its effects on internalization [33].

## SNA cytotoxicity

**Fig. 15:**
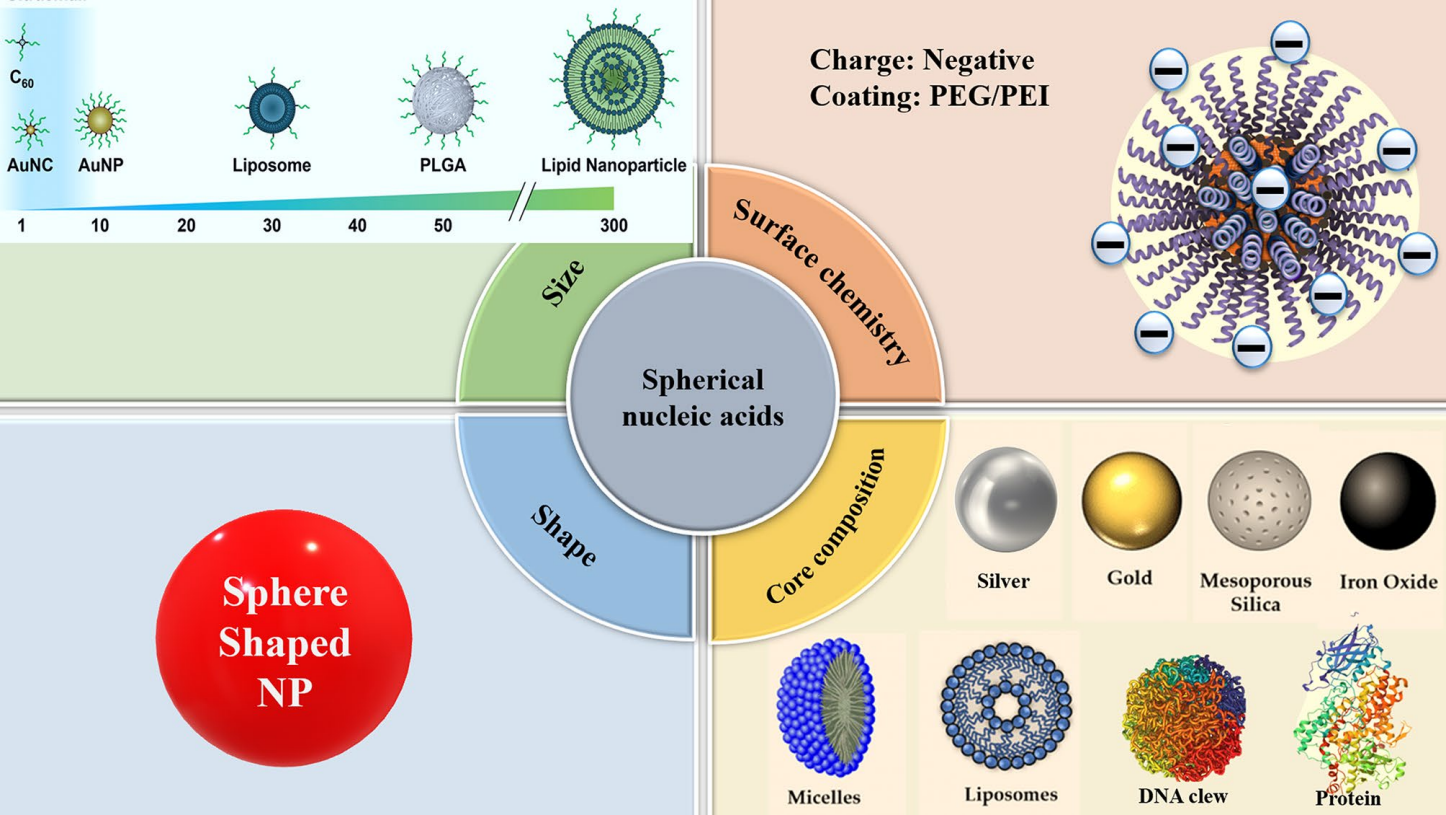
SNAs features and cytotoxicity

Different types of nanoparticles/nano-formulations, such as metallic (Au, Ag, Iron Oxide), non-metallic (protein, lipid), polymers, and biopolymers (PLGA) have been used as carriers for therapeutic agents [125]. Sometimes, NPs themselves act as drugs, while others act just as a carrier for other materials [125]. As nanoparticles enter into a biological system, a variety of interactions occur between nanoparticles and biomolecules, as well as biological pathways [125]. Some in vivo and in vitro studies suggested that despite the broad potential application of nanoparticles, they could have toxic side effects [126]. Some of the toxic effects of nanoparticles are caused by their transportation by the bloodstream or lymph stream to various organs and tissues, such as the heart, liver, brain, kidneys, spleen, nervous system, and bone marrow, which can cause tissue damage [127]. Another part is caused by their interaction with intracellular components, which leads to excessive production of free radicals and oxidative stress, lysosome damage, mitochondrial damage, membrane instability, and DNA damage [127]. Variations in size, shape, catalytic activity, surface properties, as well as core and shell compounds of NPs determine how they are absorbed, distributed, metabolized, and excreted in the human body as well as within the cell [128]. Toxicological studies reveal less toxicity of spherical-shaped NPs compared to other shapes e.g., needle-like, rod-like, plate-like, etc. [113]. Studies on cultured BEAS-2B cells showed that plate-like and needle-like NPs caused the death of a higher percentage of cells than spherical and rod-like NPs, which is partially due to inducing physical damage in cells and tissue upon direct contact [113]. Also, morphological features of the NPs affect their intracellular translocation and excretion. As shown, the sharp-shaped nanoparticles opposite of NPs with less sharpness (round shaped) could escape the endosome by breaking the endosomal membrane. Thereby, they have a low excretion rate and long-term cytosolic availability [129], leading to undesirable side effects. Additionally, it has been shown that shape influences NPs’ interactions with channels [113]. It has been discovered that unlike spherical fullerenes, single-walled carbon nanotubes could efficiently block calcium channels [113]. Dendrimer-shaped nickel NPs displayed larger toxicity to embryos of zebrafish compared to spherical NPs [130]. Plate-silver NPs showed the highest harmful effects on the bacterium Escherichia coli compared to their spherical or rod counterparts [130]. Size is another parameter that largely affects NPs toxicity [113]. Huo et al. have indicated that smaller Au-NPs (< 6 nm) are more toxic than larger ones ($\ge$10 nm) [113]. In another study, Pan et al. revealed that 15-nm NPs had less (60 times) toxicity than NPs with almost 1.4 nm in size against epithelial cells, macrophages, fibroblasts, and melanoma cells [113]. Smaller sizes allow NPs to enter not only into cells but also into cell organelles, particularly the cell nucleus, and interact with the sugar-phosphate backbone of DNA, thereby leading to transcription inhibition. So far, SNAs have been synthesized from different types of core nanoparticles in a wide range of sizes from fullerene scaffolds (1 nm) to lipid nanoparticles (300 nm) [131]. Although each of them showed unique characteristics such as oligonucleotide density on the NP surface, gene silencing activity, variable duration of blood circulation, specific targeting of organs, excretion routes, etc., they all had negligible toxicity of these constructs. The reason for this can be attributed to the size of SNAs in contrast to the type of core nanoparticles. The composition of core NPs is another critical factor in NPs cytotoxicity. Indeed, different NPs of the same size could indicate different toxic effects [126]. For example, Zinc oxide (ZnO) affects the DNA structure while Silicon dioxide (SiO_2_) induces oxidative stress [126, 132]. Usually, the toxicity of metal nanoparticles is caused by their release into the cell [126]. Some metallic NPs [e.g., Arsenic (As), plumbum (Pb), mercury (Hg), and silver (Ag)] have a toxic nature and naturally, they can be harmful to cells [126]. Others such as iron (Fe), Gold (Au), and Zinc (Zn) are counted as harmless NPs at low concentrations but in high amounts, they can lead to toxic reactions [126, 133]. It has been shown that Lipid-Based and polymer-based [e.g., polylactic glycolic acid (PLGA)] NPs have the least toxic effects for in vivo applications [134]. Therefore, they have obtained the approval of the Food and Drug Administration (FDA) for clinical use [134, 135]. Although toxicity tests have shown SNAs to be safer carriers than other nanocarriers, the level of toxicity of different types of SNA with various nanoparticles varies considerably  [136]. For example, metal nanoparticles (gold and silver) and polymers are more toxic than other nanoparticles such as proteins, DNA clew, and liposomes [136].The surface charge of the nanocarrier is the other parameter that affects toxicity [137]. Cellular absorption of nanocarriers with a positive surface charge is higher than nanocarriers with a negative surface charge [137]. Still, studies showed that negative nanocarriers were safer and less toxic than those with positive surface charge [137]. SNAs have a negative surface charge due to the oligonucleotide layer around them, but unlike other nanocarriers with electrostatic cellular internalization, their cellular entry is receptor-mediated. Therefore, these nanocarriers not only show a high level of cellular uptake but also have negligible toxicity. As a key point, it should be kept in mind that applying some changes including (1) designing structures that are specific to a tissue or cell, (2) designing programmable SNA (assembling and disassembling in a specific manner), (3) coating with PEG, and (4) synthesis of nanoparticles with non-toxic, biocompatible, and biodegradable compounds can increase the safety of SNAs even more [126, 134]. Hence, the majority of SNA’s characteristics, such as spherical shape, approximately 15-nm diameter size (especially when gold nanoparticles are used as the dominant nanoparticle core in the synthesis of SNAs), the presence of oligonucleotide shell, and PEG coating, have led these nanoparticles to present minimal toxicity. Consequently, most studies have identified SNA as a safe nanoparticle (Fig. 15) [21, 138]. Regarding the above-mentioned benefits and universal properties, SNAs have become more interesting nanoplatforms for both in-vivo and in-vitro delivery and therapeutic applications. The next sections explains the various therapeutic applications of SNAs-oligonucleotides (ASOs, miRNA, siRNA) nanoplatforms.

## Applications of SNA-oligonucleotide delivery systems

### RNA interference (RNAi)

RNA interference (RNAi) (1998) has been suggested as a specific therapeutic modality for various genetic diseases, including tumors and viral infections [139]. This post-transcriptional gene silencing pathway is triggered by small interfering RNA (siRNA), microRNA, and germline-specific Piwi-interacting RNA (piRNA) [140]. RNAi systems utilize oligonucleotides with a length of 21 to 25 bases (siRNA, miRNA), which bind to their complementary mRNAs via Watson-Crick base pairing [141], mediating the direct degradation of target mRNA and suppressing the expression of the disease-causing protein [141]. However, after many years, these molecules have not found their ways to the clinic due to the lack of a safe and efficient delivery system [141]. So far, many studies have been conducted on the clinical applications of the use of SNA nanocarriers for the delivery of therapeutic oligonucleotides, including siRNA, miRNA, and ASOs, which will be discussed below.

#### siRNA

**Fig. 16:**
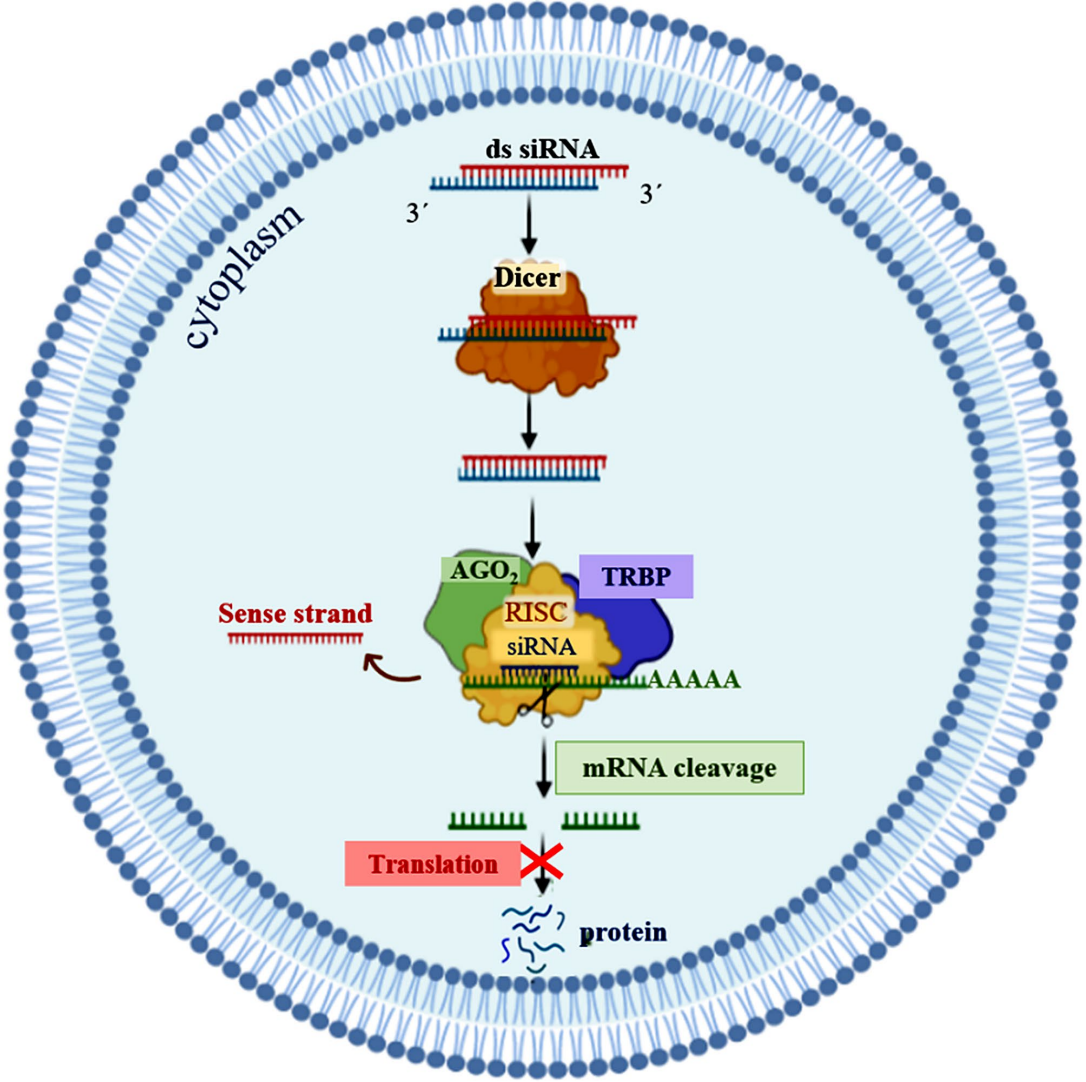
Schematic illustration of the action mechanisms of siRNAs

Short interfering RNAs (siRNA) are important exogenous synthetic molecules with potentially wide biomedical applications [139]. They are RNA molecules with a normally 19–21 nt duplex and 2-nt overhangs at both 3′-ends of each strand [140]. Findings suggested and confirmed that siRNAs are mediators of transcriptional gene silencing with conserved pathways in mammalian cells [142]. In the cytoplasm of eukaryotic cells, siRNAs develop an enzymatic machinery in company with several protein units, named the RNA-induced silencing complex (RISC), in which the strands are separated, the sense (or passenger) strand is degraded and driven out from RISC, the anti-sense (or guide) strand remains in the RISC to bind to its complementary mRNA, where Argonaut endoribonuclease promotes mRNA cleavage and prevents it from being translated into protein [139, 140] (Fig. 16). There are many challenges with siRNA delivery, including instability, cellular uptake, off-target effects, and the lack of suitable delivery vehicles for passing these negatively charged biomacromolecules across the cell membranes [140]. Theapplicability of SNAs as a nanocarrier has been studied for siRNAs. In the following, various types of SNA nanoplatforms that have been presented for the delivery of siRNAs in both in vivo and/or in vitro systems will be discussed.

#### Spherical nucleic acids nanoplatforms for siRNA delivery

Sinegra et al. [58] generated a type of lipid nanoparticle SNA structure (LNP-SNAs), in which LNP cores were prepared from a series of lipids, consisting of an ionizable lipid, phospholipid, lipid-PEG, and cholesterol. Then, nucleic acids cargos were loaded into LNPs via the ethanol dilution method. Next, 3′-SH DNA was attached to the LNPs that contained lipid- PEG-maleimides by mixing. In this study, the activity of LNP-SNAs in the siRNA delivery and silencing luciferase gene (Luc2) were assessed. The results suggested that optimized LNP-SNA decreased the siRNA concentration required for silencing the target mRNA (twice in magnitude) compared to liposome-based SNAs. Also, the ability of these compositions (LNP-SNAs) in nucleic acid delivery in vivo was investigated by encapsulation of luciferase (Luc) mRNA by detecting luciferase protein. The outcome referred that LNP-SNAs functionalized with G-quadruplex DNA affected its biodistribution profile in mice, as encapsulated mRNA was expressed mainly in the liver and lesser in the spleen. Overall, the data indicated that LNP-SNAs influenced the activity and biodistribution of siRNAs and may increase their safety and efficacy (Table 2) [58].

**Fig. 17:**
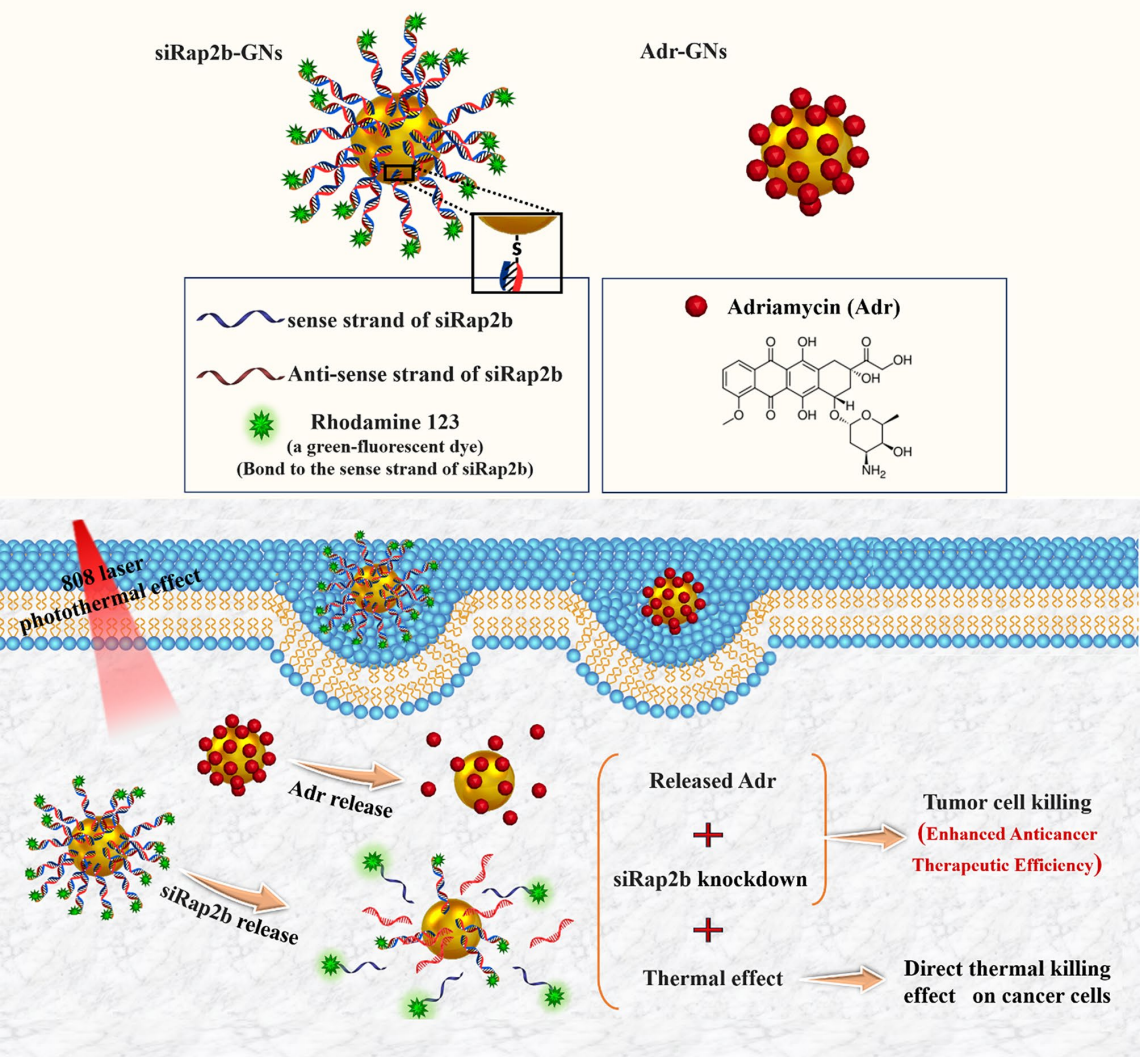
The design of a delivery system containing adriamycin (Adr) and synthesized siRap2b conjugating with gold NPs. Treating drug-loaded GNs with an 808-nm laser generated a photothermal effect, which deeply enhanced drug release. The released drug (Adr) showed direct toxicity against cancer cells. Furthermore, isolated siRap2b remarkably diminished the expression of Rap2b and boosted anticancer therapeutic efficiency. Additionally, the thermal effects arising from laser directly suppressed cancer cells/tissues. This figure was redrawn with permission from ref [143]

Multi-drug resistance is one of the major problems in cancer chemotherapy [143]. Studies indicated that Rap2b (belonging to the p53 family) promotes resistance to Adriamycin in tumor cells [143]. So, Ding et al [143]. designed a (gene/ drug) nano-carrier for Rap2b siRNA delivery to evaluate its therapeutic potential against human cancers. In this nanostructure, a gold nano-shell (GNs) was prepared with reducing Chloroauric acid (HAuCl_4_) onto silver NPs and then PEGylated via thiol-Au bonds. Subsequently, Adriamycin (Adr) (via amino-Au bond) and siRap2b molecules (via thiol-Au bonds) were attached to the PEGylated GNs and formed Adr-GNs, siRap2b-GNs. Data demonstrated that the co-delivery of both Adr and siRap2b with GN particles into cancer cells decreased Rap2b expression by siRap2b, boosting the anticancer therapeutic efficiency of Adr. Overall, both the in vivo (HCT116 tumors in a nude mouse model) and in vitro (HCT116 cells) results showed a similar anti-tumor therapeutic efficiency for Adr- and siRap2b-loaded GNs (Table 2) (Fig. 17) [143].

Nonhealing skin wounds are one of the side effects of type 2 diabetes (T2D), which can lead to bacterial infections and oblige amputation [144]. Ganglioside-monosialic acid 3 (GM3) has been recognized as a critical mediator for insulin resistance [144]. Randeria et al. [144] introduced an approach for treating diabetic wounds based on siGM3S-SNA. Thereby, homologous siRNA duplexes of (murine and human) GM3S mRNAs were synthesized and incorporated with a gold NP core to develop GM3S-targeting SNAs. In this approach, by topical delivery of siGM3S-SNA, the expression of GM3S mRNA and protein was efficiently and specifically suppressed in cultured keratinocytes (KCs), as well as both in wounded and intact mouse skin, followed by KC migration toward wound position, the phosphorylation of insulin-like growth factor-1 receptor (IGF1R) and epidermal growth factor receptor (EGFR), and fast healing of wounds in T2D mice. This investigation suggested that the nanoplatform could be used for treating any skin lesions and diseases with a genetic basis (Table 2) [144].

Glioblastoma multiforme (GBM) is an aggressive and lethal type of cancer that can develop in the brain or spinal cord [145]. Previous studies indicated that the 19q13 locus, related to the Bcl2Like12 (Bcl2L12) oncogene, is a commonly amplified region in GBM [145]. Bcl2L12 is involved in inhibiting apoptosis by deactivating caspase-3, caspase-7, and apoptosome and obstructing the activity of the p53 tumor suppressor, thereby directing tumorigenesis in the brain [145]. Jensen et al. [145] developed an SNA–gold NP functionalized with Bcl2L12-targeting siRNAs (siL12-SNAs) as a biotherapeutic for Glioblastoma. These SNA nanoplatforms indicated an efficient systemic delivery tool for targeting intracerebral tumors and infiltration through the blood-brain barrier/ blood-tumor barrier (BBB/BTB). Also, siL12-SNAs enabled successful, specific, and persistent Bcl2L12 gene knockdown in vitro and in vivo and reduced the mass of tumors in mouse models without undesirable effects or toxicity. Finally, SNAs were suggested as a potential delivery system to the CNS for treating other nervous diseases and CNS tumors (Table 2) [145].

In another research, Melamed et al. [146] developed PEI–SNAs for targeting the Gli1 oncogene, a transcriptional activator within the Hedgehog signaling pathway which is crucial for the maintenance of glioma stem cells (GSCs) and also plays a key role in the development and chemo-resistant of GBM. PEI – SNAs nanostructure was prepared in a defined method by attaching Gli1 siRNA. The activity of PEI–SNAs was investigated by introducing them into U-87 MG cells (malignant gliomas). Results suggested that Gli1 PEI-SNAs were successfully picked up by U-87 MG cells via scavenger receptors, silenced the expression of Hedgehog signaling pathway genes like Gli1 and Smo (∼ 30%), subsequently leading to a ∼ 30% reduction in expression of downstream transcriptional target genes of Gli1 (e.g., CyclinD1, c-Myc, Bcl2, and ABCG2), and eventually, prevented the progression of chemotherapy-resistant GBM by reducing the proliferation of GBM cells (30%), as well as their metabolic activity (∼ 60%) and self-renewal capability (30–40%) (Table 2) [146].

In a recent study, Chen et al. [147] developed a photolabile SNA (PSNA) for cancer gene therapy (GT). PSNA is manufactured from a hydrophilic shell of hypoxia-inducible factor-1α (HIF-1α) siRNA, which is conjugated with hydrophobic peptide nucleic acid (PNA) core containing B-cell lymphoma 2 (Bcl-2) ASO via O_2_-cleavable linker. Also, near-infrared (NIR) photosensitizer (PS) is co-assembled in the core through hydrophobic interactions, which makes possible the photo-regulation of desired processes and triggers PSNA disassembly in living organisms. NIR leads to O_2_ production and subsequent breaking of O_2_-cleavable linker,  thereby promoting the disassembly of PSNA into its components (siRNA, pASO, and PS). In this study, HIF-1α and Bcl-2 were selected as targeted for gene therapy because they were involved in tumorigenesis by activating the expression of various oncogenes and antiapoptotic activity, respectively (Table 2) [147].

In vitro and in-vivo activities of PSNA were evaluated in human cervical cancer (HeLa) cells and mice enduring subcutaneous HeLa tumors, respectively. The results confirmed that this combinational system, including NIR light irradiation and PSNA, could suppress HIF-1α and Bcl-2 in gene therapy. Furthermore, released PS inhibited tumor cell growth successfully by combined photodynamic therapy (PDT). Thereby, the recent nano platform was proposed as a carrier-free, designable, NIR-controllable, and biocompatible system for self-delivering various therapeutic agents, including oligonucleotide drugs, either alone or along with other small-molecule drugs  [147].

**Fig. 18:**
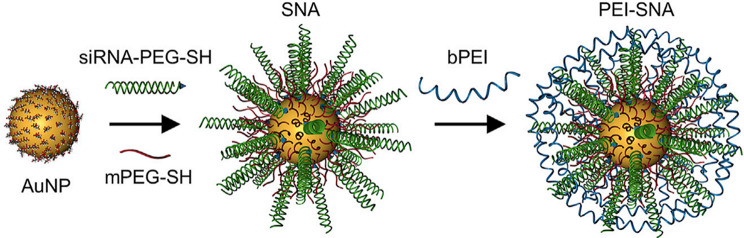
The model describing the synthesis of SNAs and PEI-SNAs. Permission was received from ref [138]

Melamed et al. [138] introduced a unique architecture of SNAs with surface PEI presentation as a hybrid delivery system, named polyethyleneimine-coated spherical nucleic acids (PEI-SNAs), in which PEI enclosesd an NP core with a radial orientation of GFP targeting siRNAs. In this class of NPs, citrate-capped 15 nm AuNPs were made by the Frens method and subsequently, siRNA and polyethylene glycol (PEG) were grafted on the NPs via thiolated ligands. Then PEI-SNAs were formed via electrostatic adsorption of PEI (Fig. 18). Assays revealed that complete PEI-SNAs construction contained 38 siRNA duplexes per particle and 923 PEI molecules per each PEI-SNA (Table 2) [138].

The characteristics of the PEI-SNA (siRNA) delivery systems, such as cellular internalization, intracellular trafficking, toxicity, and gene silencing efficacy, were compared with those of PEI-siRNA polyplexes in U87-MG glioma cells [138]. Data indicated higher cellular uptake, accumulation within lysosomes, and better gene silencing potency (GFP gene) relative to polyplexes [138]. Additionally, cytocompatibility investigations showed PEI had lower toxicity in the form of PEI-SNAs versus polyplexes. These results suggest that the architecture and surface chemistry of siRNA nanocarriers play a notable role in their cellular interactions and eventual gene regulation efficiency (Table 2) [138].

Tumor necrosis factor-alpha (TNF-a) belongs to a class of proinflammatory cytokines overexpressed during inflammation, leading to the initiation and persistence of inflammatory diseases such as autoimmune diseases (e.g., psoriasis) [148]. Hosseinzadeh et al. [148] designed a spherical nucleic acid nanoparticle conjugate (SNA-NCs) for regulation of the TNF-α gene. In this approach, gold NP synthesis with citrate reduction method (chemical method), was use to prepare TNF-a siRNA duplexes attached on gold NPs by mixing and sonicating; oligonucleotide coverage increased on the gold NP surface by salt aging; SNA-NC formation procedure was completed by adding polyethylene glycol into the siRNA–NPs solution. Finally, in vitro experiment illustrated that SNA-NC significantly decreased TNF-α gene expression in Murine macrophage RAW264.7 cells without cytotoxicity [148].

**Fig. 19:**
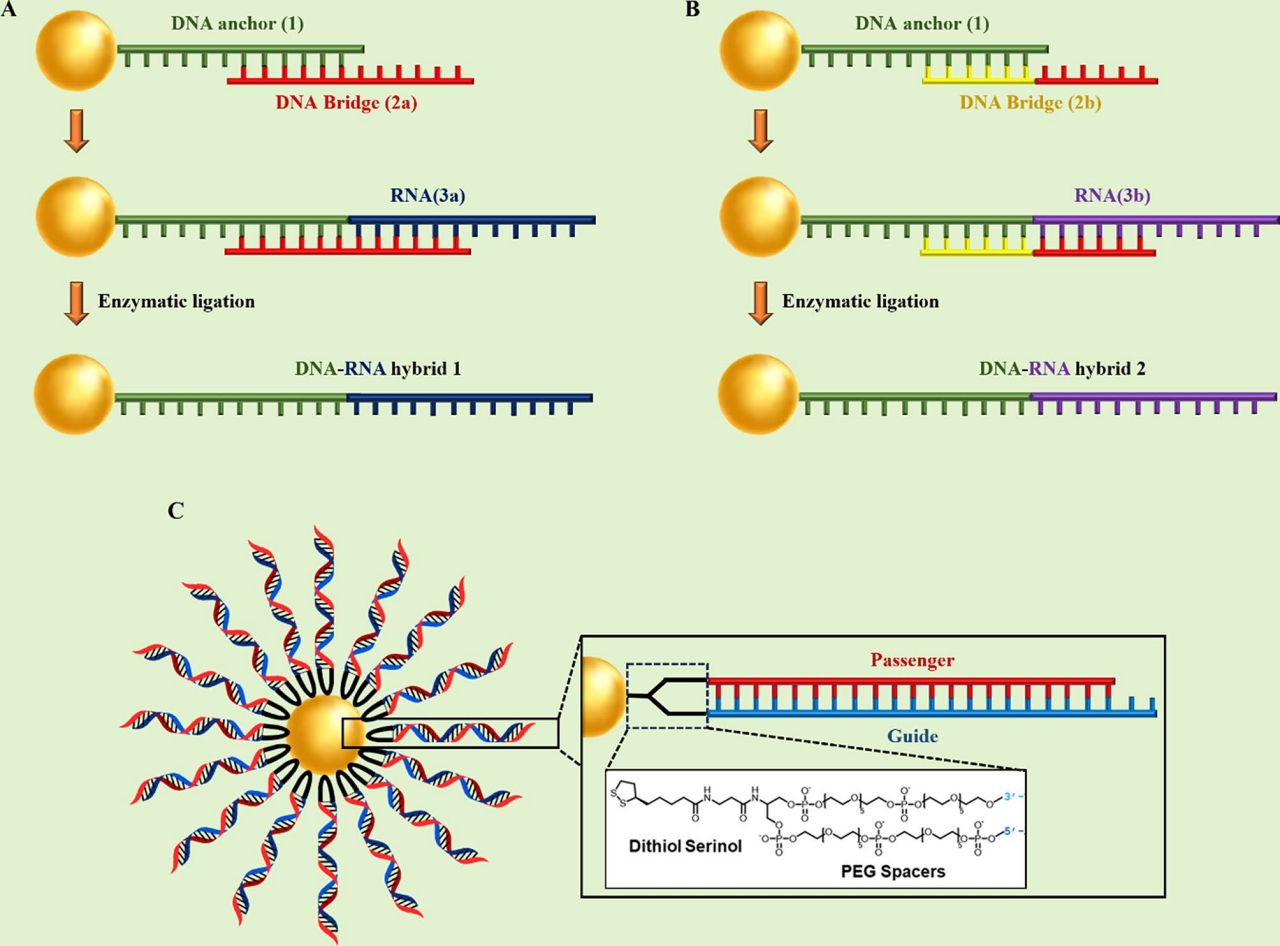
(**A, B**) RNA assembly onto a universal SNA scaffold via an enzymatic method [149]. (**A**): SNA is made up of an Au NP core functionalized with a DNA “anchor” oligonucleotide [1]. The DNA “bridge” (2a), another DNA oligonucleotide matching to the 3′ end of 1 (anchor). Following the hybridizing with 1, the sticky end formed by the DNA bridge (2a) is utilized to guide the hybridizing and assembly of the 5′ end of an RNA (3a) to the SNA surface. (Note: for siRNA sequences, this strand is the sense strand of a siRNA duplex). (**B**): By altering the sticky end sequence of the DNA bridge located at its 3′ end (2b), it is possible to match the sequence with the 5′ end of another RNA oligonucleotide (3b). This process enables the assembly of diverse RNA sequences on an SNA coated using the same DNA anchor [1]. (**C**) Hairpin-like design, a hairpin-like siRNA, a single RNA strand made of a duplex, and a hairpin-like region of PEG spacers, are bound to the core with high duplexing efficiency [150]. This figure was redrawn with permission from the mentioned references

Rouge et al. [149] presented an approach in which siRNAs were covalently attached to DNA-based SNA. In this SNA scaffold, a gold-NP core (13 nm) was functionalized with a 5´ hexyl dithiolated DNA oligonucleotide (named DNA anchor); the second DNA oligonucleotide (named DNA bridge) was attached to a 3´ complementary end of DNA anchor, and a 3´ sticky end was generated by a DNA bridge that could be programmed for attaching to various RNA sequences and mediating RNA assembly on the NP surface. RNA ligation on the DNA-based SNA was accomplished with a T4 DNA ligase-catalyzed reaction (Fig. 19A, B). In this study, the therapeutic function of in vitro transcribed siRNA(s) (GFP, GAPDH) in both single and dual-ligated SNA nanoplatforms were evaluated in Hela and C166 GFP-expressing cells. Data suggested that the enzymatic ligation strategy did not influence the biochemical targeting and function of the siRNA(s). Moreover, GFP/GAPDH siRNA SNA(s) efficiently knocked down their two different target genes in a single, dual-conjugated NP construction (Table 2) [149].

Lately, Vasher et al. [150] designed and synthesized a new SNA, where siRNA strands were directly grafted on the SNA core via a hairpin-like architecture (Fig. 19C). This architecture prevents guide strand detachment. As a result, hairpin-like siRNA-SNAs demonstrated a higher number of siRNA duplexes grafted onto SNA (4-fold), a longer half-life (6-fold) in serum, and decreased cytotoxicity versus the original hybridized siRNA-SNA construction. Gene silencing efficiency was evaluated in hybridized and hairpin-like siRNA-SNAs containing sequences targeting HER2, Luc, and VEGF. Data suggested that hairpin-like siRNA-SNA delivered more persistent gene knockdown compared to hybridized siRNA-SNAs. Furthermore, the chemistry and architecture of immobilized siRNAs were observed to noticeably influence the biological function of SNAs. so the hairpin-like architecture was suggested as an SNA construct that would be helpful for medical applications  (Table 2) [150].

siRNA instability is a major challenge in achieving effective regulation of gene expression in vitro and in vivo [151]. Research has shown that serum nucleases can identify and cleave the grafted siRNA at specific sites near the Gold NP surface (4 nm) [151]. Barnaby et al. [151], in a strategy for improving the stability of siRNA-spherical nucleic acids, developed an SNA nanoplatform comprising of AuNPs which functionalized with siRNA targeting androgen receptor in serum (SNA-siRNA-AR) containing nucleotides with 2′-O-methyl modification at recognition sites of nuclease SNA-siRNA-AR. In this study, the interaction of serum nucleases with the mentioned SNA-siRNA-AR was analyzed based on the capacity of knocking down human androgen receptor (AR) expression in lymph node carcinoma of the prostate (LNCaP) for prostate cancer (PC) therapy. As a result, siRNA lifetime increased (10-fold) when the 2′-O-methyl modification was introduced to the nuclease restriction sites of siRNAs in the SNA-siRNA-AR nano platform (Table 2) [151].

Epidermal growth factor (EGF) is involved in epidermal homeostasis, which promotes cell growth and differentiation by joining to its receptor (EGFR) and starting signaling pathways [152]. Previous studies illustrated the overexpression of EGFR signaling in many malignancies, skin inflammation, and dominant-negative genetic skin disorders [152]. So, the EGFR gene can be a primary target for gene silencing [152]. For gene silencing in the skin, topical delivery of a therapeutic agent may be an optimal approach that provides easy access to the desired site of the skin and decreases the risk of systemic side effects [152]. Zheng et al. [152] presented spherical nucleic acid nanoparticle conjugates (SNA-NCs), in which the gold NP core served as a scaffold for grafting a dense shell of highly oriented siRNA targeting epidermal growth factor (EGFR). Experiments were achieved to assess the EGFR-suppressing efficiency of SNA-NCs in human keratinocyte cells (hKCs), mouse model (SKH1-E) skin, and human skin models. Results showed that SNA-NCs easily enter into keratinocytes and successfully suppressed EGFR gene expression and downstream ERK phosphorylation in both in vitro and in vivo experiments. In vivo, no clinical or histological toxicity was observed in skin, and no cytokine activation was detected in blood or tissue samples obtained from mice. So, SNA-NCs were suggested as a promising tool for topical gene delivery in skin-related diseases (Table 2) [152].

**Fig. 20:**
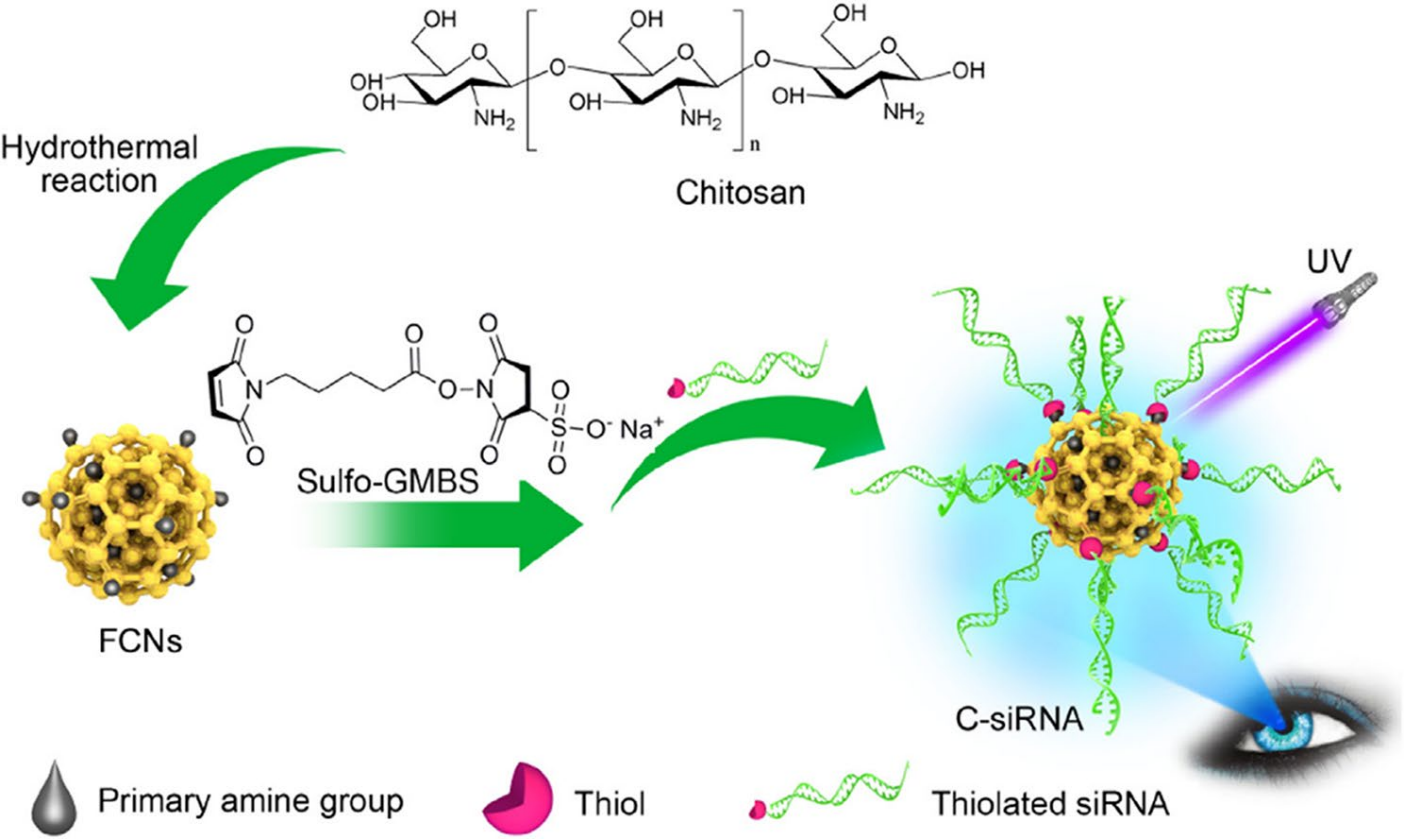
Scheme of the manufacturing procedure of C-siRNA. Permission was received from ref [153]

Zhang et al. [153] developed fluorescent carbon nanoparticle (FCN)-based siRNA conjugates (C-siRNA) for gene regulation in cancer therapy. FCNs are a kind of carbon-based materials that have high photostability, low cytotoxicity, biocompatibility, which are rapidly removed from the body in vivo. So, these characteristics make FCNs attractive for diagnostic and therapeutic applications. In this study, FCNs core NPs were synthesized via the carbonization of the chitosan, as nanoscale particles, and by hydrothermal reaction at a mild temperature (180 C). The surface of FCN received post-modifications with amine groups. Then, thiolated siRNAs targeting the polo-like kinase-1 (Plk1) gene (siPlk1) were covalently attached to the amine group of FCNs to form C-siPlk1. Finally, maximized coverage of the siRNA on the FCNs was obtained by the slow addition of NaCl (Fig. 20). Plk1 is a major regulatory factor in mitosis and is upregulated in cancer cells such as human breast (MCF-7) and melanoma (A375) cancer. It seems that the suppression of Plk1 expression can promote cancer cell apoptosis and prevent tumor development. So, the function of C-siPlk1 was assessed in MCF-7 and A375 cell lines, as well as in melanoma Balb/c nude mice. The results showed that the C-siPlk1 conjugant was successfully internalized by tumor cells without an axillary agent and suppressed the Plk1 gene both in vitro and in vivo with much higher efficiency than non-viral gene delivery methods such as Lipofectamine 2000. Furthermore, it was noted that an identical amount of siRNA could be delivered with a much lower dosage of FCNs compared to Gold NPs (∼ 1/ 30). Data suggested that the assembling of the fluorescent FCN nanoparticle core and the therapeutic siRNA could be useful not only for therapeutic goals but also for imaging and diagnostic purposes (Table 2) [153].

Intra-portal islet grafts are the preferable intrahepatic islet transplantation method because of portal vein accessibility and low morbidity [154]. However, about 50% to 70% of transplanted cells are rapidly lost due to the activation of the early innate immune response and the production of proinflammatory cytokines (e.g., interferon (IFN)-γ, interleukin (IL)-1β, and tumor necrosis factor (TNF)-α) [154]. Proinflammatory cytokines are primarily activated by the IκB kinase (IKK) subunit (IKKβ) of NF-κB [154]. A previous study indicated that repressing NF-κB activity impeded the abnormal function of the islet, and improved intra-portal survival and β cell function [154]. Common procedures for inhibiting NF-κB activity in human islets have some drawbacks that limit their benefits [154]. For instance, auxiliary transfection agents like lipid-based materials or viral vectors are unable to deliver genetic therapeutic agents to the cells located in the core of islets. Additionally, these methods can be toxic at high concentrations [154]. The small inhibitory molecule must be used systemically, which leads to harmful effects on non-islet tissues [154]. In a study,  Rink et al. [154] designed and prepared siRNAs for suppressing IKKβ and then conjugated them to a gold NP core to form IKKβ-SNA-nanoconjugates. Finally,  IKKβ SNA-NC knockdown efficiency was assessed in isolated islet cells (in vitro) and also in intra-portal transplantation mice. In this study, the effective function of IKKβ SNA-NCs in reducing IKKβ expression and preventing NF-κB activation was confirmed in islet cells. The results of syngeneic intraportal cell transplantation, treated with IKKβ SNA-NCs, in streptozotocin-induced diabetic rats, showed that the knockdown of IKKβ expression by IKKβ increased the engraftment and function of the post-transplantation islets. In addition, histological analyses confirmed reduced CD11b-expressing cell infiltration and decreased islet apoptosis. Therefore, pre-treatment of newly isolated islets with IKKβ SNA-NCs was proposed as a promising way to enhance islet engraft function and post-transplant survival (Table 2) [154].

#### MicroRNA

**Fig. 21:**
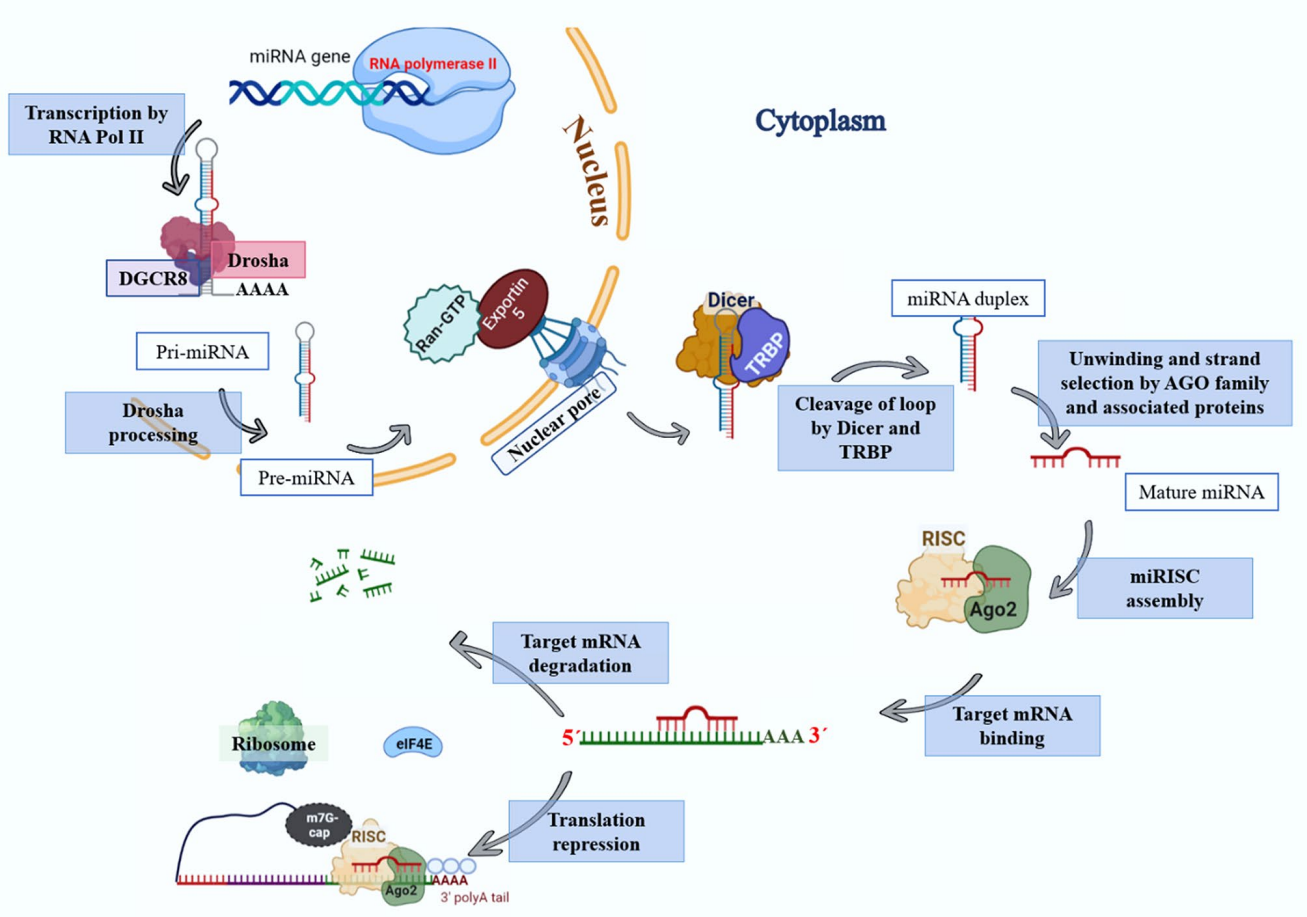
Schematic illustration of miRNA biogenesis and mechanism of function

MicroRNAs are mostly tissue- and temporal-specific and have highly conserved sequences. MicroRNAs guide gene regulation via attaching to the 3′-untranslated regions (3′-UTRs) of target mRNAs, thereby mediating degradation or translation inhibition of their targets [155]. However, some cases suggested that miRNA could bind to 5′ untranslated regions (5′UTRs) or exons and even DNA elements and enhance translation and transcription, respectively [156]. It is worth mentioning that microRNA could be involved in the regulation of the expression of multiple mRNAs, unlike siRNAs which could interfere with the expression of just one specific target mRNA [155]. In well-regulated pathways, microRNA genes are transcribed by RNA polymerase II (Pol II) into long miRNA transcripts (pri-miRNAs) that are long and have stem-loop structures [155, 157]. In the nucleus, the Pri-miRNAs are processed by Drosha ribonuclease and DGCR8 in to pre-miRNA (structure of the 70–100 nucleoid series) [156]. Later, the pre-miRNA is exported into the cytoplasm by Exportin-5 and Ran [155]. Each of the pre-miRNAs is cleaved by the DICER enzyme, and a mature miRNA duplex consisting of 22 nt is formed [43]. A mature miRNA duplex attaches to the miRNA-induced silencing complex (miRISC), the passenger strand is degraded but the guide strand, which is associated with Argonaut (AGO) protein in the miRISC remains and can bind to the 3′-UTR of the target mRNA [155, 156]. It is noticeable that miRNA molecules could bind to mRNAs, having only 2–7 complementary nucleotides in the “seed region” [156, 158]. Indeed, they do not need perfect complementary to bind to target mRNA [156]. After miRNA binding to target mRNAs, two probable gene silencing pathways can occur, depending on the range of miRNA and mRNA base pairing, in which bounded mRNAs may be degraded or stored in processing bodies (p-bodies) for later translation (Fig. 21) [156, 159].

#### Spherical nucleic acid nanoplatforms for miRNA delivery

**Fig. 22:**
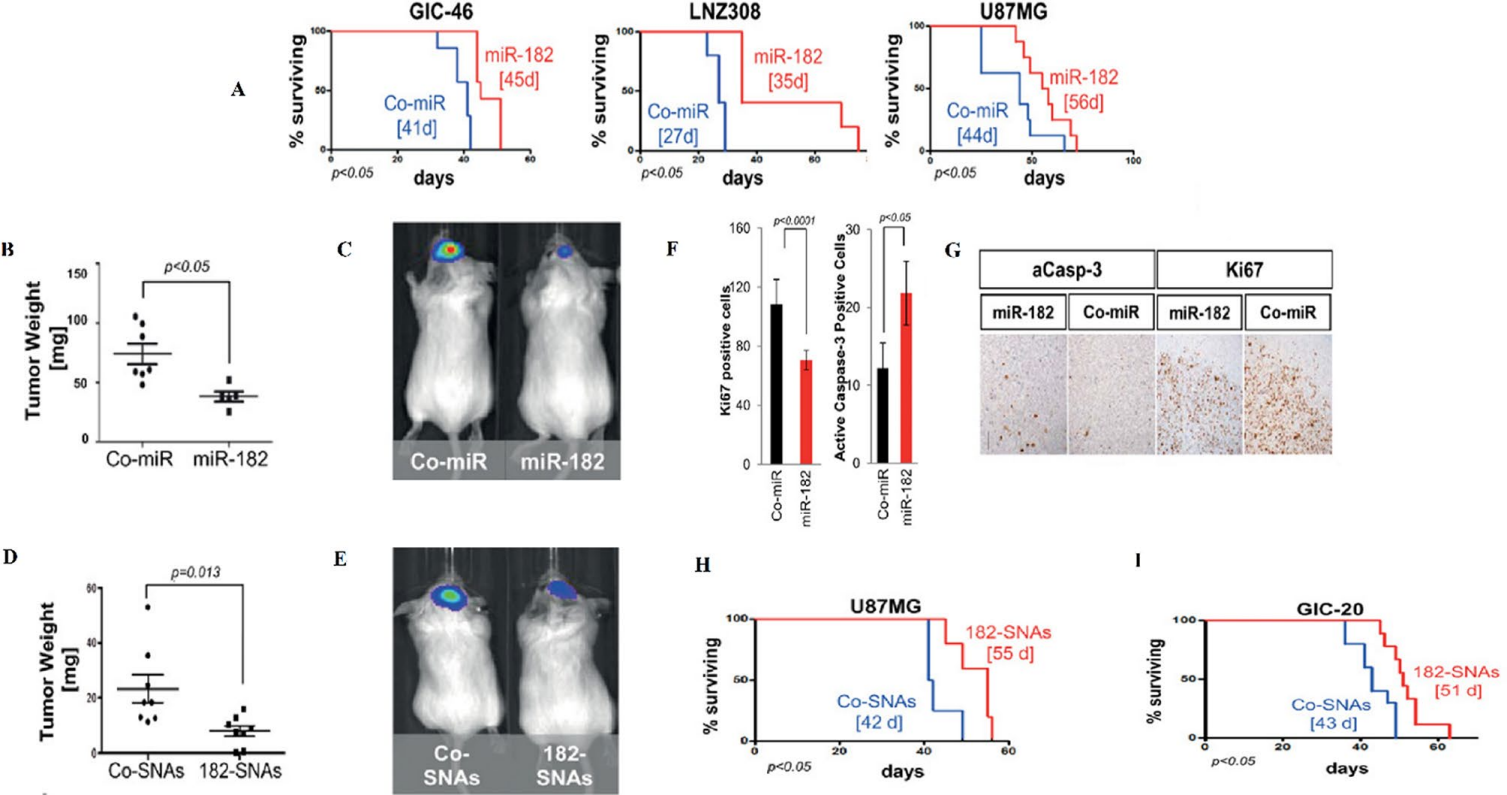
(**A**) Survival analysis indicated that miR-182 expression increased the survival of animals (rthotopic xenograft with glioma cells and engineered GICs that stably expressed miR-182). (**B and C**) Tumor burden analysis via weight and bioluminescence imaging. (**D**) Weight of tumors derived from U87MG xenografts extracted from SCID mice 21 days after intravenous treatment with Co-SNAs or 182-SNAs. (**E**) Bioluminescence imaging of xenograft tumors derived from GIC-20 (12 day) after intravenous treatment with Co-SNAs or 182-SNAs. (**F**) Estimation levels of Ki67 and caspase-3 in xenograft samples. (**G**) Ki67 and caspase-3 IHC in coronal brain sections of GIC-derived xenografts expressing Co-miR or miR-182. (**H and I**) Kaplan-Meyer survival estimator curves of SCID mice xenografted with glioma tumors (U87MG and GIC-20)  and intravenously treated with Co-SNAs or 182-SNAs. Permission was received from ref [160]

Many studies have suggested that miR-182 is a crucial tumor suppressor and prognostic factor in glioblastoma [160]. Also, miR-182 plays a role in differentiation and determining cellular fate during neurodevelopment, as well as the apoptotic response of glioma cells against different types of anti-cancer agents (e.g., temozolomide [TMZ] and RTK inhibitors) by targeting various networks of genes [160]. In such a way, glioma cells expressing miR-182 promote glioma-initiating cell (GIC) differentiation and diminish cancer cell proliferation by downregulating the caspase and p53 inhibitor, Bcl2L12, c-Met, and HIF2A [160]. Hypoxia-driven stem cell maintenance (HIF2A) and c-Met (a member of the RTK family) are important factors in promoting GIC self-renewal, growth, cancer development, and their expressions are associated with decreased glioma patient survival [160]. miR-182 targets a conserved site at the 3’UTR of HIF2A and c-Met, which leads to a decrease in stem cell markers expression and cell proliferation, directing cells toward a differentiated state [160]. So, Kouri et al. [160] developed SNAs consisting of a 13 nm gold NP core and a shell of miR-182 sequences (miR-182-SNAs). In vitro experiments demonstrated that miR-182-SNAs were efficiently internalized and significantly reduced the levels of Bcl2L12 and c-Met proteins in gliomas. In vivo, by intravenously administrating miR-182-SNAs into various orthotopic GBM xenograft models, miR-182-SNAs bypassed the blood-brain/blood-tumor barrier (BBB/BTB), decreased tumor burden, and increased survival of animal models. In another study, Kouri et al. [160] prepared miR-182-SNAs, in which AuNPs were covalently functionalized with mature miR-182 duplexes. By intravenous administration of miR-182-SNAs to GBM xenografts (glioma-bearing mice model), miR-182-SNAs successfully infiltrated from the blood–brain/blood–tumor barriers (BBB/BTB). Investigations on LN229, LNZ308, and U87MG cell lines derived from xenografted tumors referred that miR-182-SNAs downregulated the expression of the Bcl2L12 gene in GBM through binding to the 3′ UTR of Bcl2L12, exerting inhibitory effects on caspase-3, and caspase-7, and finally, resulting in (1) a reduction in tumor burden, by reducing the number of proliferating (Ki67) and enhancing the number of apoptotic (caspase-3) cancer cells, and (2) improving animal survival in vivo without noteworthy undesirable side effects [160]. So, systemic delivery of miR-182-SNA was suggested as a promising miRNA-based treatment for brain malignancy (Table 2; Fig. 22) [160].

**Fig. 23:**
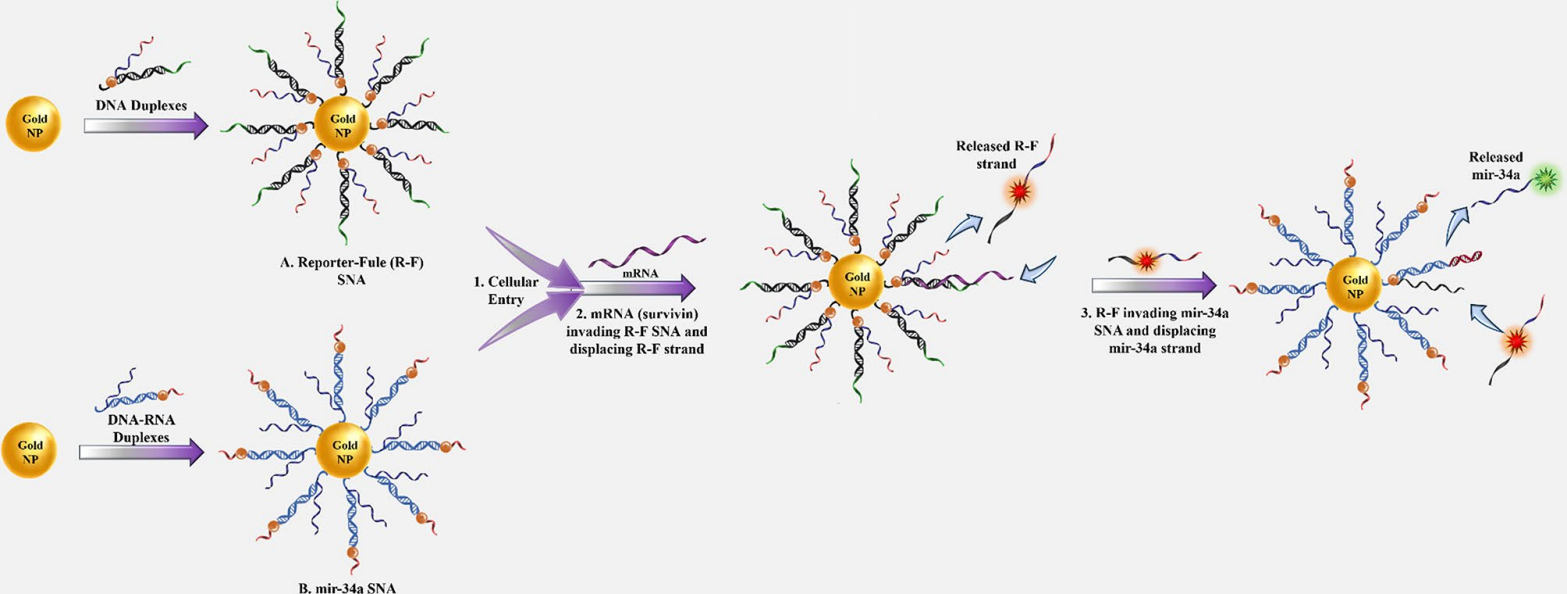
The graphical design rationale of delivery and releasing miR-34a, employing SNA nanocarriers in MCF-7 cells. This figure was redrawn with permission from ref [161]

Li et al. [161] synthesized two series of spherical nucleic acid (SNA) nano-carriers (named R-F SNA and miR-34a SNA) for delivering microRNA-34a to MCF-7 breast cancer cell to target survivin mRNA. miRNA-34a (miR-34a) is a kind of tumor suppressor. Several studies indicated that the overexpression of miR-34a could suppress the mRNAs associated with cell proliferation, migration, and invasion in different cancer cells. In this study, both Reporter-F SNA and miR-34a SNA contained gold NP core. The thiolated oligonucleotide shell in R-F SNA included DNA duplexes, which was antisense to the survivin mRNA transcript and contained the reporter (black section) and overhang Fuel domains (blue and red section) in the miR-34a SNA DNA-RNA duplexes. Upon cellular internalization of both types of SNAs, survivin mRNA invades R-F SNA, and the R-F strand of R-F SNA is displaced and released. Then, the free R-F strand invades miR-34a SNA and releases the miR-34a. The displaced miR-34a mimics can moderate the apoptosis of MCF-7 cells. Data confirmed that in MCF-7 cells treated with R-F SNA and miR-34a SNA, increased miR-34a level reduced survivin mRNA level (∼ 77%) compared to the control untreated group and led to apoptosis and reduced cell viability, implying the high efficiency and accessibility of the SNA-based miRNA delivery system (Table 2; Fig. 23) [161].

**Fig. 24:**
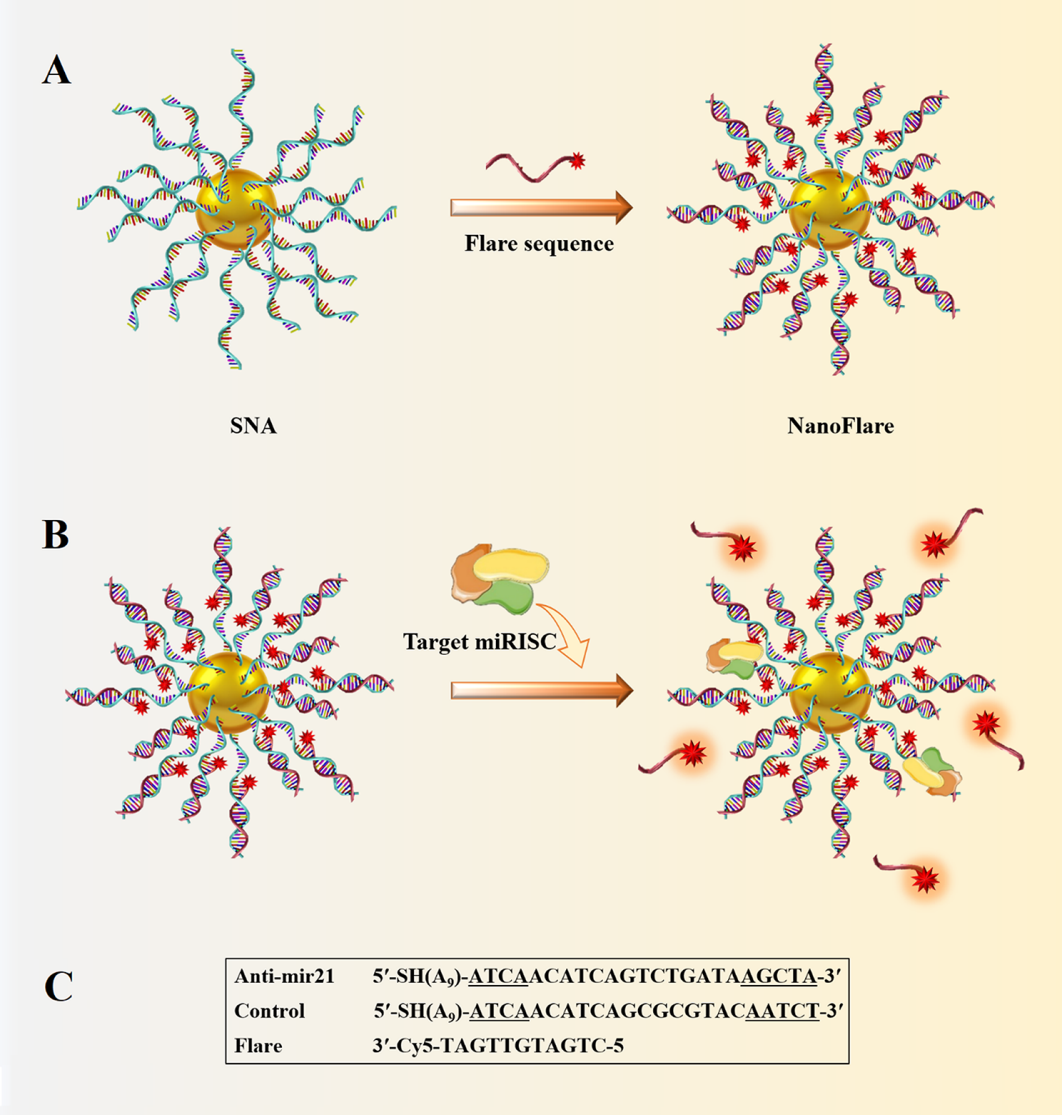
Preparation of spherical nucleic acids (SNAs) conjugated with flare sequences: (**A**) Functionalization of Au nanoparticles (13 ± 1 nm) surface with propylthiol-terminated antisense DNA, hybridizing with short complementary fluorophore-labeled DNA (Flare). (**B**) Upon incubation of the prepared nano-conjugates (**A**) with complementary miRNA targets, RISC (miRISC) was loaded, and the fluorescence signal increased. (**C**) The DNA/LNA gapmer sequences (target and control) served as antisense strands (Note: The *underlined* bases are LNA), and the flare sequence was applied to form nanoflare conjugates. This figure was redrawn with permission from ref [162]

miR-21 has been implicated in many forms of cancer such as prostate cancer (PC) [162]. Also, it has been shown that miR-21 at high expression levels stimulates cancer cell proliferation, invasion, and metastasis by downregulating many of its target genes, including Phosphatase and Tensin Homolog (PTEN), Tropomyosin 1 (TPM1), and Programmed Cell Death 4 (PDCD4), which are involved in tumor-suppressing [162]. According to this, Alhasan et al. [162] have developed nanoflare-SNAs structures for targeting miR21 (anti-miR21), which could be used for both detecting intracellular levels of miR-21 and reducing expression of the miR21. In this study, Nanoflare-SNAs were synthesized from (A) gold nanoparticles (13 ± 1 nm), and (B) hybrids of thiol-terminated antisense DNA (anti-miR21 sequences) with fluorophore-labeled DNA (Flare). Then these structures were enclosed within exosomes, a class of nano-sized bio-vesicles (diameter of ∼ 100 nanometers) that play central roles in the maintenance and transportation of endogenic macromolecules, such as microRNAs and mRNAs (Fig. 24). Observations referred that loading anti-miR21 SNA nanoconjugates within exosomes as transfection agents led to anti-miR21 Exo-SNA formation, which could selectively target PC-3 cancer cells in cell culture and knocked down miR-21 more efficiently (≈ 3000 times) relative to the same concentration of free miR-21-SNAs. So, Exo-SNA structures are suggested as potent miRNA regulatory agents in PC-3 prostate cancer cell lines (Table 2) [162].

**Fig. 25:**
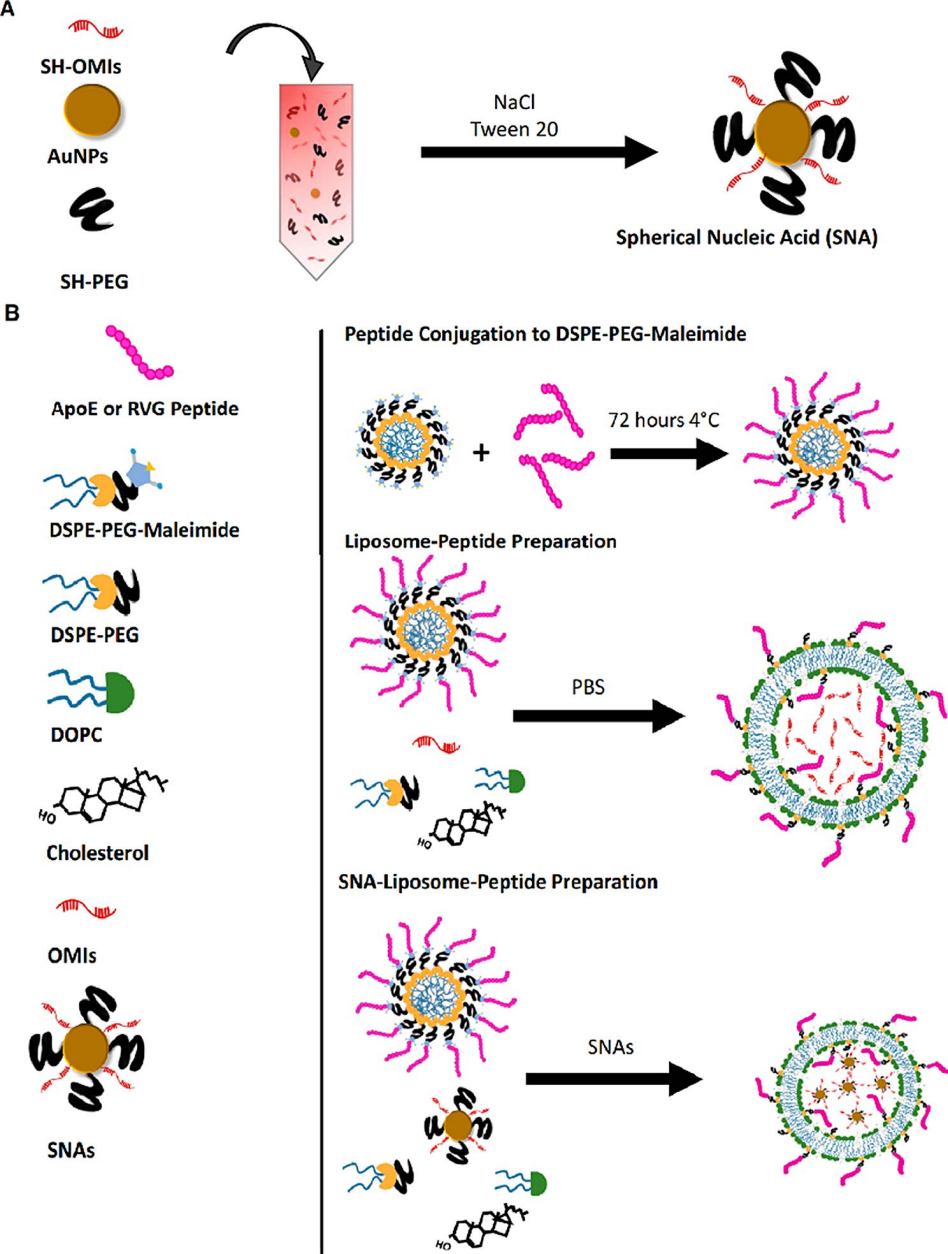
Schematic illustration of the synthetic process of peptide-hybridized gold-liposome. (**A**) Spherical nucleic acids (SNAs) formation. (**B**) Construction of Liposome-Peptide and SNA-Liposome-Peptide nanocarriers. Permission was received from ref [163]

Ruiz et al. [163] introduced gold-liposome NPs, which were coupled with peptides targeting apolipoprotein E (ApoE) and rabies virus glycoprotein (RVG) in the brain. AuNPs were functionalized with oligonucleotide miRNA inhibitors (OMIs)  to form SNAs. Then, SNAs were enclosed within ApoE, RVG-conjugated liposomes, and SNA-Liposome-ApoE and SNA-Liposome-RVG structures were obtained, respectively (Fig. 25). The properties and delivery efficiency of each NP were evaluated in U87 GBM cells in vitro and in GBM syngeneic mice. Data indicated that SNA-Liposomes with ∼30–50 nm in diameter were successfully internalized into U87 GBM cells and inhibited the expression of miRNA-92b, a carcinogenic miRNA overexpressed in GBM cell lines and GBM tumors. Also, the results revealed that coupling SNA-liposomes with ApoE or RVG peptides boosted the systemic delivery of the construction into the brain tumors of GBM syngeneic mice. However, SNA-Liposome-ApoE showed notable enhancement in intra-tumor accumulation by crossing the BBB/BTB, showing higher inhibition efficiency toward miR-92b relative to other nano-formulations created, including controls, SNA-Liposomes, and SNA-Liposome-RVG. Thus, SNA-Liposome-ApoE was suggested as an optimal vehicle for future RNAi-based therapies against GBM and other CNS disorders (Table 2) [163].

**Fig. 26:**
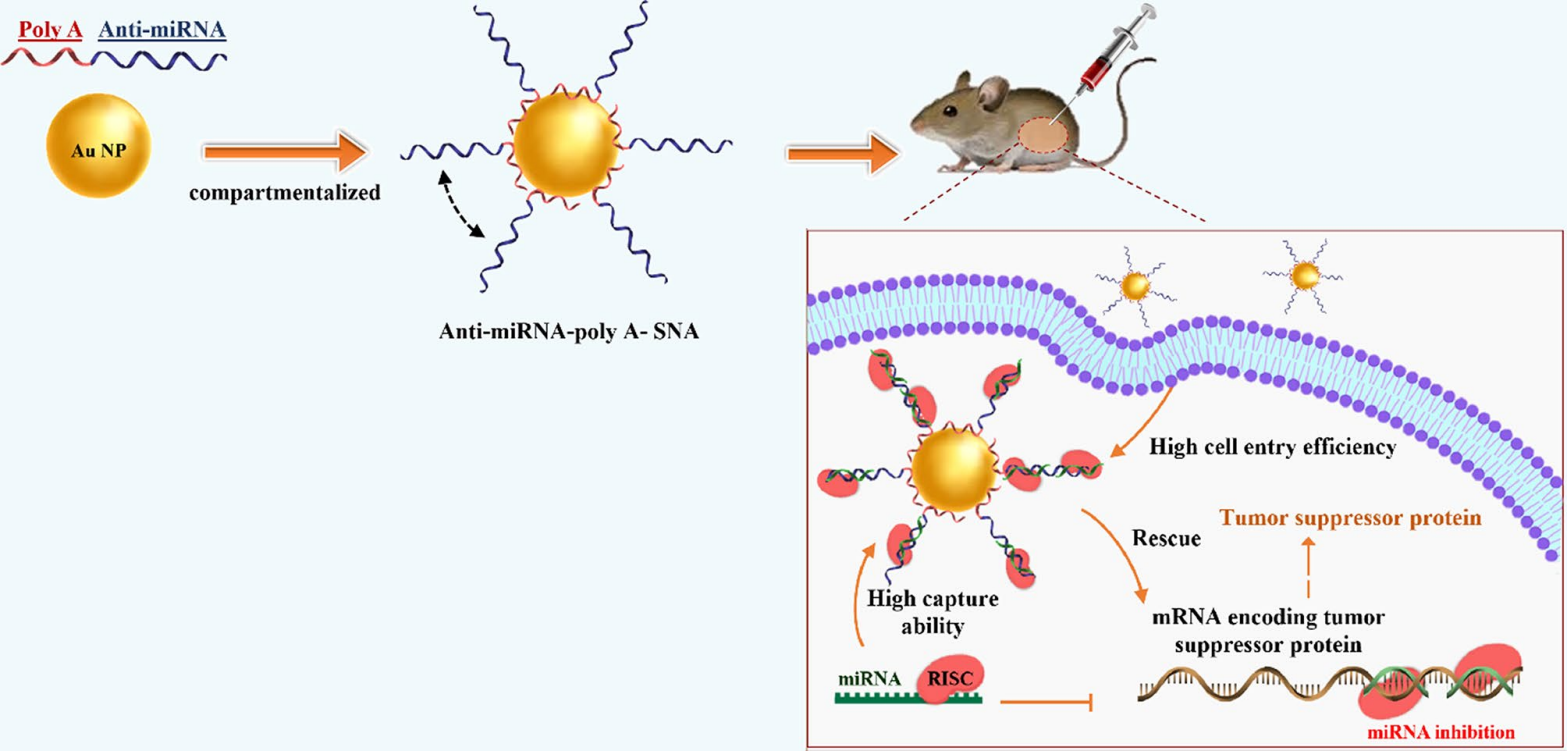
Schematic design of PolyA-SNAs construction containing programmed polyA-lateral spacing for recovery of the antioncogene expression. This figure was redrawn with permission from ref [165]

Jiao et al. [164] developed polyadenine-based spherical nucleic acids (polyA-SNAs) for the effective capture of oncogenic miR21 in living cells. Briefly, polyA-SNAs synthesized from DNA strands consisted of two domains: 1) the anti-miR21 domain, which was overexpressed in different types of tumors (blue lines), and 2) polyA (A5 to A40) domain (red lines), which were attached to AuNPs (∼ 15 nm diameter) with the salt-aging process (Fig. 26). The polyA domain can tightly attach on gold-NPs due to the high affinity between AuNPs and adenines. In this study, the function and the miRNA capture capability of polyA-SNAs were evaluated in several systems, including 1) HEK293T cells, which were transfected with labeled mir21 and then treated with anti-mir21-polyA-SNAs (derived from A5, A15, or A30), 2) 293T cells, where plasmid (containing the EGFP gene and six copies of anti-mir21 sequences at the 3’ UTR served as the reporter for mRNA rescue), and exogenous mir21 were co-transfected in 3) MCF-7 cancer cell line, in which Tropomyosin 1 (TPM1) as an antioncogene was suppressed by mir21 overexpression, and 4) MCF-7 tumor-bearing mice. Experiments suggested that proper spatial arrangement of anti-miRNA sequences in polyA-SNAs could be achieved by regulating the length of polyAs. Thereby, programmed polyA(A15 in this study)-SNAs enhanced targeted binding and capture ability compared to densely assembled SNAs. Also, data confirmed that polyA-SNAs, especially A15-SNA, could effectively rescue the expression of mRNAs suppressed by the target miRNAs (miR21). Furthermore, it was approved that treatment with anti-mir21 A15-SNA successfully inhibited oncogenic mir21 in mice, rescued the antioncogene TPM1, and suppressed tumor growth without noticeable toxicity while showing high stability in the tumor environment. So, polyA-SNA was suggested as a system with numerous benefits such as programable conformation, allowing for the optimization of their capture ability and cell entry efficiency (similar to other densely assembled SNAs), leading to efficient in vitro/in vivo delivery, and obviating the need for axillary transfection agents, which led to no or negligible toxicity. Finally, it was suggested that polyA-SNA could be used for the delivery of exogenously synthesized oligonucleotide in diagnostic and therapeutic applications (Table 2) [164].

**Fig. 27:**
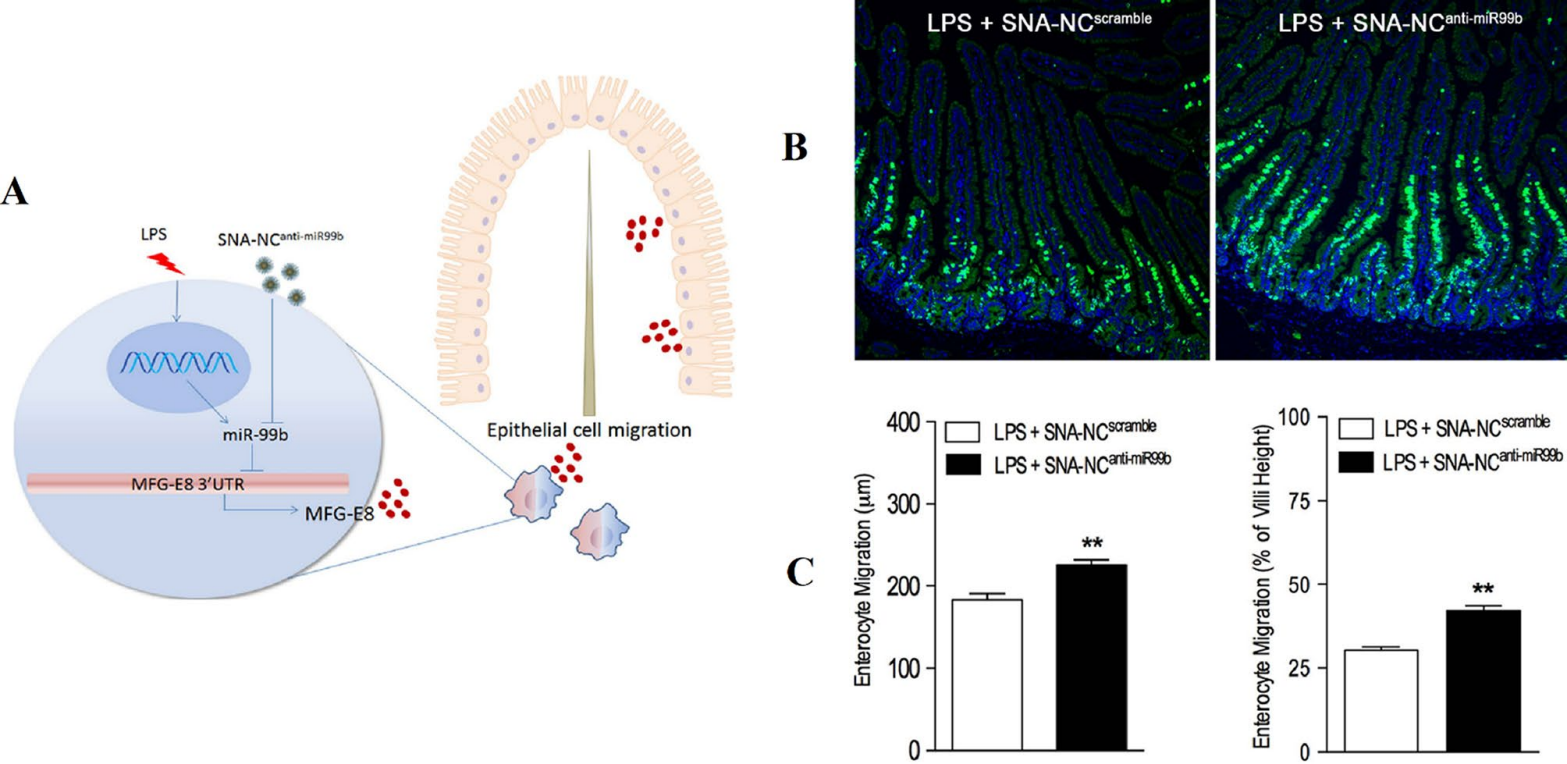
(**A**) Schematic model depicting the effects of targeting MFG-E8 gene expression in macrophages on intestinal epithelial homeostasis. Inhibition of miR-99b stimulated the migration of intestinal epithelial cells in LPS-induced septic mice. (**B**) Images of the small intestine processed for BrdU/DAPI staining. (Note: green and blue colors show BrdU-labeled cells and nuclei, respectively). (**C**) Quantitative analysis discovered that SNA-NC anti-miR99b treatment increased enterocytes’ migration along the crypt-villus axis in LPS-induced septic mice. *n* = 3. **P. permission was received from ref [165]

Migration of epithelial cells is vital in conserving intestinal epithelial homeostasis, which is impaired in several diseases like sepsis [166]. Milk fat globule-EGF factor 8 (MFG-E8) is involved in maintaining intestinal homeostasis by increasing enterocyte migration and decreasing inflammation. Previous studies showed that MFG-E8 was downregulated in lipopolysaccharide (LPS)-induced sepsis [166]. Wang et al. [166] illustrated that treating RAW264.7 cells (a murine macrophage-like cell line) with LPS elevated the expression level of miR-99b, which in turn suppressed the expression of molecules associated with cell proliferation and wound healing by attaching to their 3′UTR sites (e.g., IGF-1R, mTOR, AKT1, and MFG-E8). Thus, they prepared SNA nano-conjugate for targeting anti-miR99b (SNA-NCanti-miR99b) and introduced it into both RAW264.7 cells and later C57BL/6 J mice (male, specific pathogen-free 7 week-old). Both in vitro and in vivo observations revealed that by administrating SNA-NC anti-miR99b, intestinal MFG-E8 expression was successfully rescued, leading to the migration of intestinal epithelial cells along the crypt-villus axis (Fig. 27). Eventually, SNA-NC anti-miR99b was suggested as a novel system for rescuing the expression of MFG-E8 and keeping intestinal epithelial homeostasis in sepsis (Table 2) [166].

**Fig. 28:**
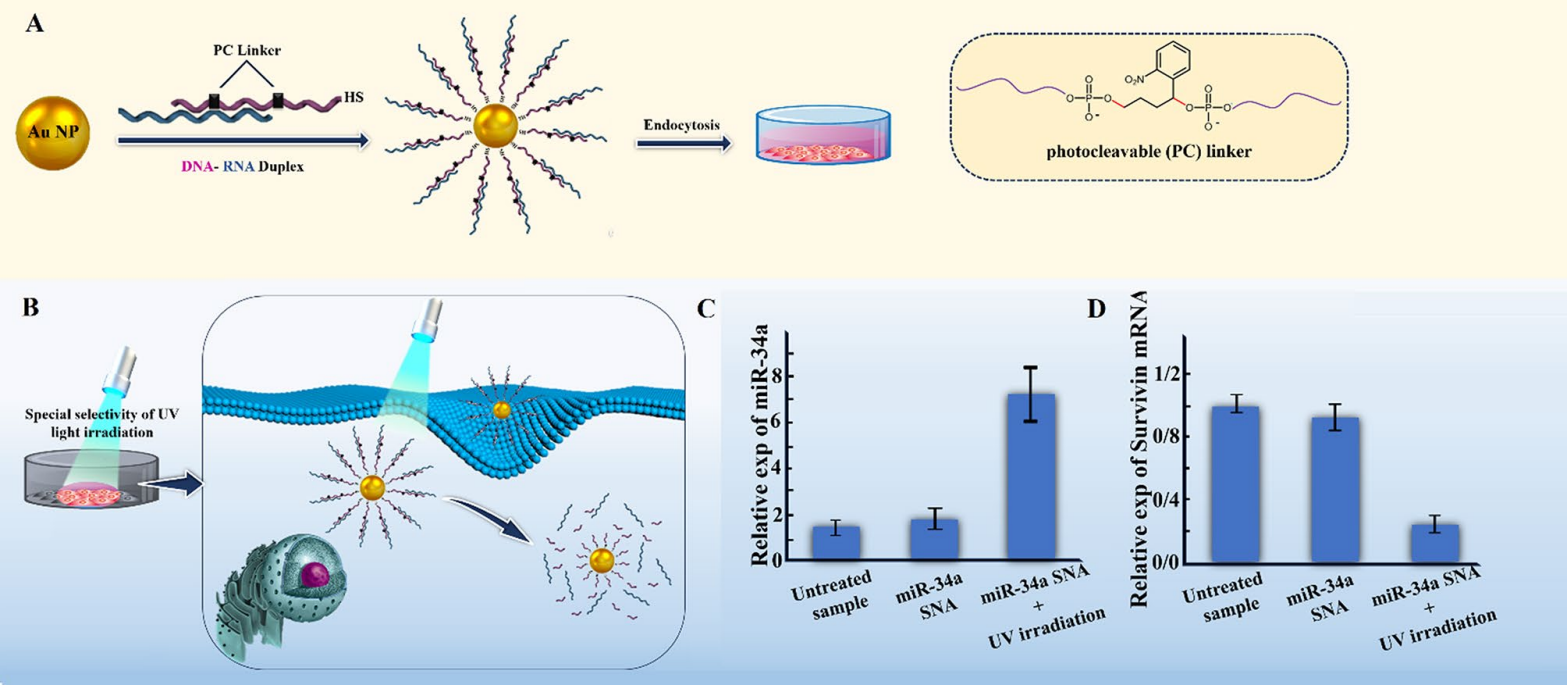
Construction of photo-responsive SNA. (**A**) Schematic illustration of the preparation of photo-responsive SNA nanocarriers. (**B**) schematic representation of the use of photo-responsive SNA and miR-34a release in MCF-7 cells exposed to UV light. **C, D**) Relative expression level of miR-34 and knockdown level of survivin mRNA. This figure was redrawn with permission from ref [167]

Zhou et al. [167] synthetized photoresponsive SNA conjugates to achieve the spatial controlling release of the therapeutic oligonucleotide via a photocleavable (PC) linker. In this system, thiolated DNA strands (purple) containing two PC linkers (black) were hybridized with mature miR-34a (blue); then DNA/RNA duplexes were grafted on the surface of AuNPs (13 ± 1 nm) (Fig. 28). Previous studies demonstrated that the up-regulation of miR-34a could suppress numerous key genes involved in the proliferation, migration, and invasion of MCF-7 cells (e.g.; Notch, LMTK3, and survivin). So, the function of photoresponsive SNA nanocarriers were evaluated in the MCF-7 cell line. By applying UV light (∼ 365 nm) radiation, PC linker was cleaved, and miR-34a strands were released; free miR-34a strands efficiently triggered the knockdown of the target gene (survivin), reduced cell viability, and encouraged apoptosis. Therefore, photo-responsive SNA was suggested as a highly efficient and novel strategy for intracellular target sensing, nano-construction designing, and gene regulation (Table 2) [167].

Ekin et al. [168] engineered a nanocarrier for miRNAs delivery, where 13-nm gold-NPs were functionalized with hybrid strands of thiolated oligonucleotides and homo sapiens precursor-miR-145 (Hsa-pre-miR-145). The effectiveness of the hybridization of miRNAs with the complementary oligonucleotides on gold-NPs was elevated by applying appropriate temperature. Hsa-miR-145 is tumor suppressor miRNA, which has been notably downregulated in numerous types of tumors, including breast and prostate cancers. Synthesized AuNP-oligo-miR-145 were transfected into both human prostate cancer cell line (PC3) and human breast cancer cell line (MCF-7). The results demonstrated the effective transfection of miR-145 into breast /prostate cancer cells with negligible cytotoxicity. Furthermore, the formation of AuNP-RNA-miRNA constructions at 72 °C led to more efficient delivery of miR-145 [168]. Also, it was demonstrated that heating at 94 °C and then 72 °C still caused robust expression of miR-145 in both MCF7 and PC3 cells (Table 2) [168].

**Fig. 29:**
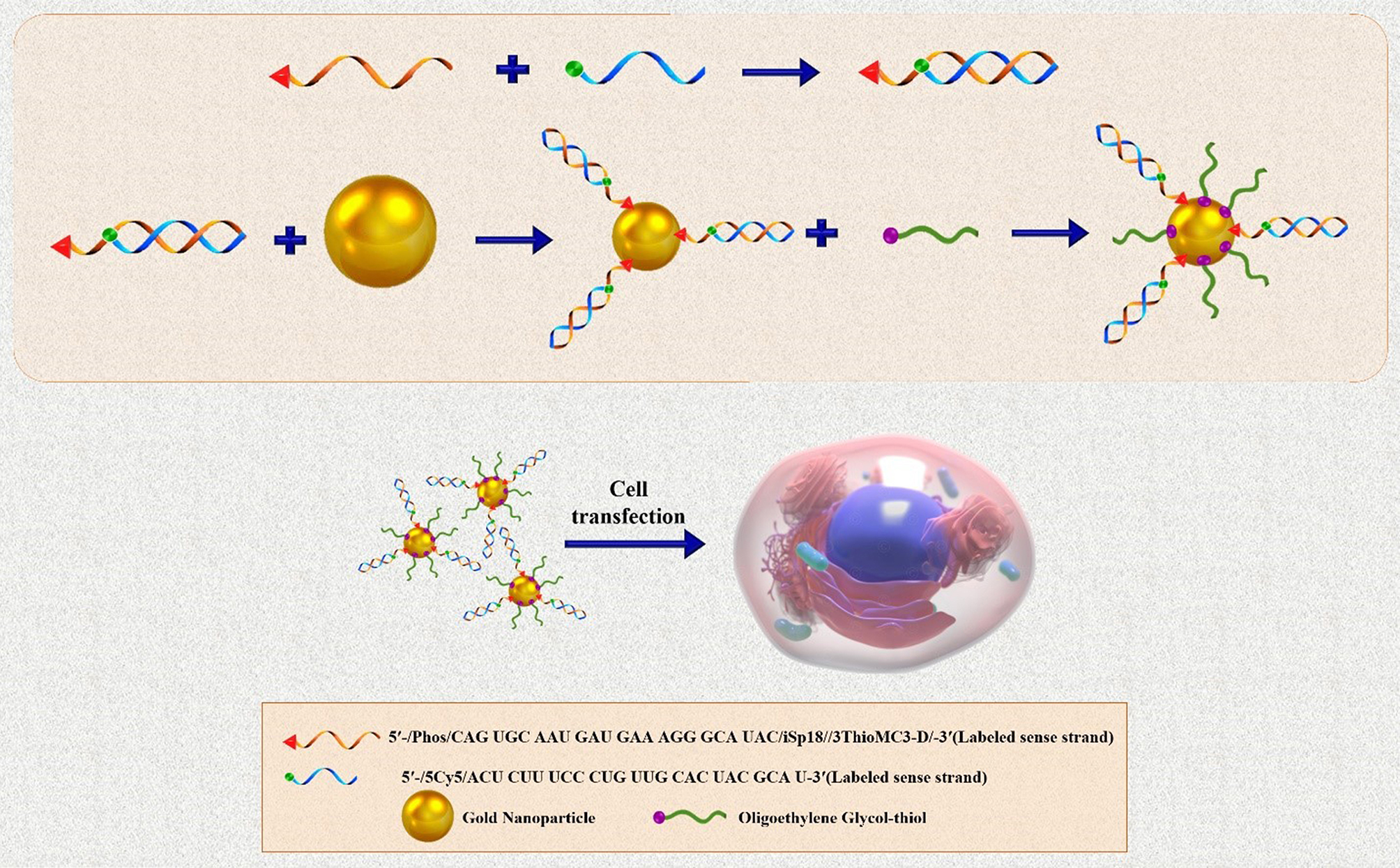
Schematic display of synthetizing of miRNA − AuNP nano-conjugates for carrying miRNAs into Cells. This figure was redrawn with permission from ref [169]

Previous studies have shown the overexpression of miR-130b in a multiple myeloma cell line (MM.1 S), promoting resistance to glucocorticoid treatment by decreasing glucocorticoid receptor protein (GR-α) expression and inhibiting glucocorticoid-induced apoptosis [169]. Given this fact, Crew et al. [169] introduced a miR-130b-Au nano-conjugate and investigated its cell transfection and knockdown efficiency. In this nanocarrier, the surface of citrate-capped AuNPs (13 nm diameter) was modified with thiolated miR-130b duplexes (∼15 microRNA per NP) (Fig. 29). miR-130b-Au nano-conjugate was functionally evaluated via luciferase assay, in which the delivered miR-130b-Au could suppress luciferase reporter gene generated by a GR promoter (3′UTR) linked reporter. As a result, the miRNA − AuNP platform demonstrated relatively high stability, efficient transfection in MM cells, and observable knockdown efficiency despite low concentration of miRNAs (∼15) per nanoparticle (a 4% coverage of miRNAs on the NPs) and decrease gene expression, which was correlated with the concentration of miRNAs on the surface of NP (Table 2) [169].

#### Antisense oligonucleotides (ASOs)

**Fig. 30:**
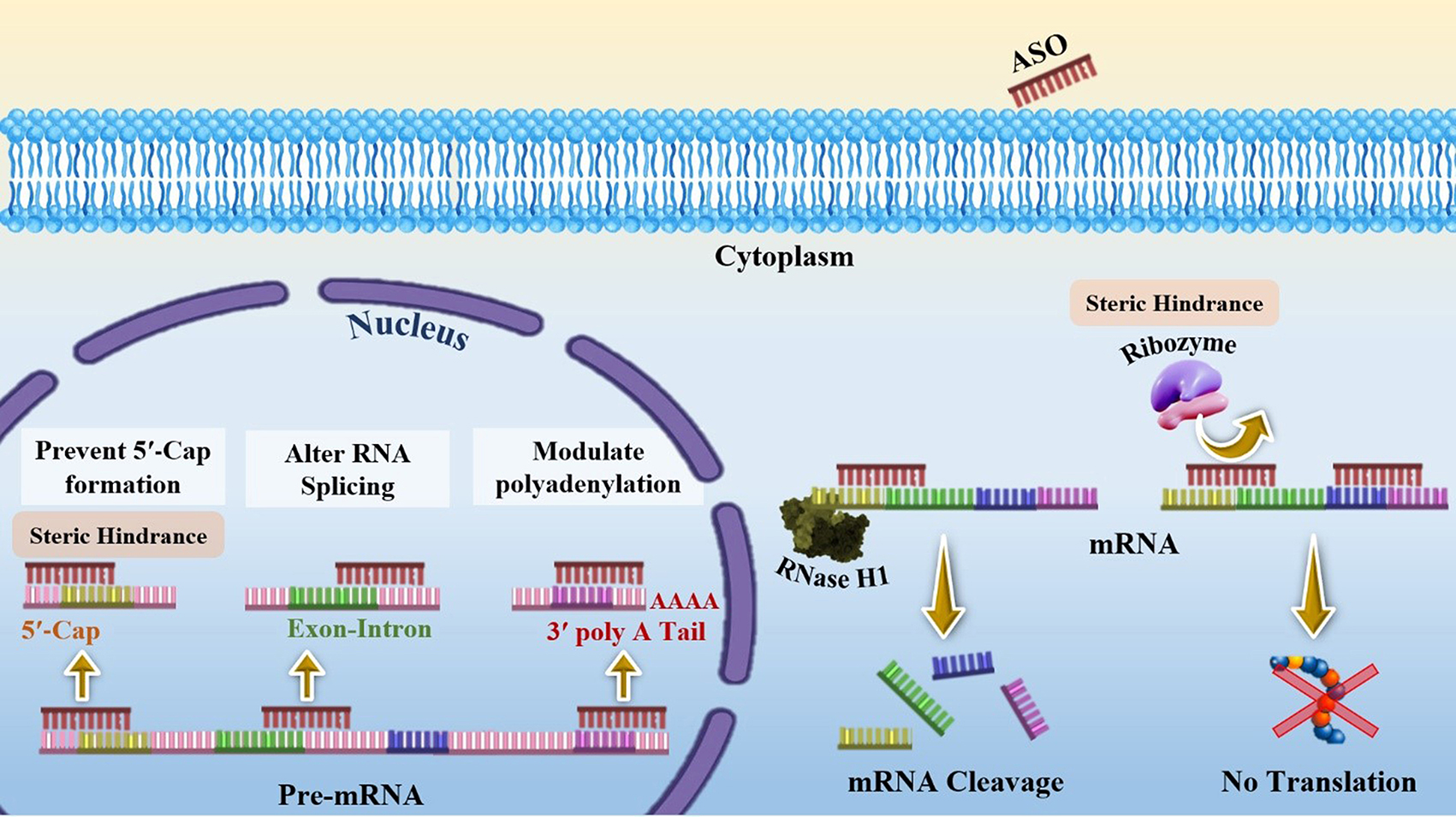
Mechanisms of action of antisense oligonucleotides (ASOs)

Antisense oligonucleotides (ASOs) are synthetic and short single-stranded deoxynucleotides (15–20 nt) [170] presenting antisense sequences (3´ to 5´) to their target mRNAs, thereby can bind the complementary mRNA via Watson–Crick base pairing and form an RNA–DNA heteroduplex [171]. Subsequently, inhibiting gene expression at the transcriptional and/or translational level is warranted through several distinct mechanisms. In the usual ASO-driven mechanism, RNase H endonuclease cleaves the RNA-DNA hybrid, leading to target mRNA degradation, decreasing translation (Fig. 30). Other mechanisms are achieved by: 1) inhibiting 5′ cap formation, binding of ASO to complementary sequences at the 5’ UTR prevents binding of the translation initiation factor eIF-4α and assembling the translation machinery, interfering with 5´cap-dependent translation, 2) altering the splicing process (splice-switching), in which wrong splicing is corrected through promoting selective expression of a variable spliceosome and blocking the splicing sites, and 3) creating steric block against ribosomal activity to inhibit the translation of the mRNA.

Today, the ASO technology is used for developing ASOs- based drugs for a wide variety of diseases, including inflammation, metabolic diseases, cardiovascular diseases, infectious diseases, cancers, and even rare diseases. The following section will discuss the applications of SNA nanoplatforms for ASO delivery.

#### Spherical nucleic acid nanoplatforms in antisense oligonucleotides (ASOs) delivery

**Fig. 31:**
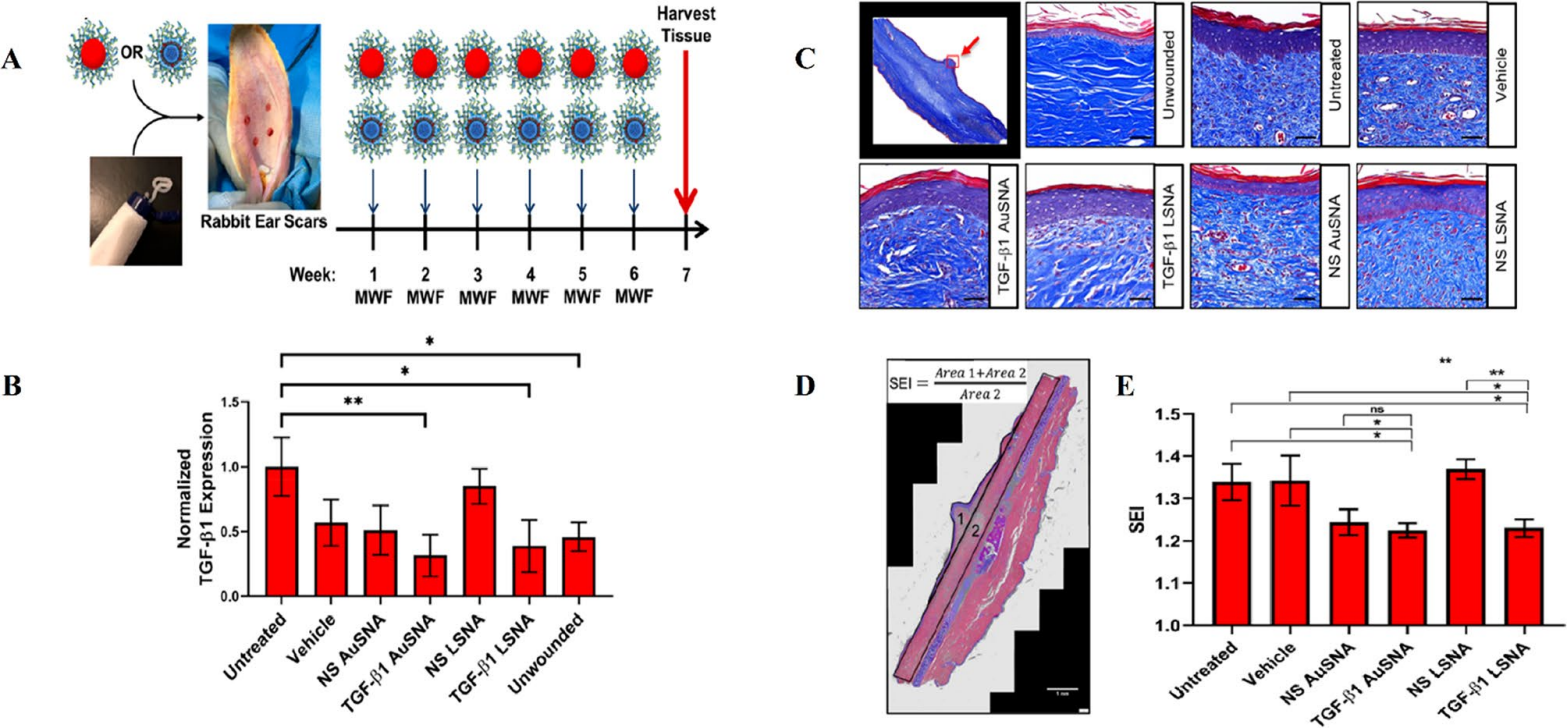
(**A**) Topical application of SNAs on rabbit ear scars. Note: “MWF” indicates 3 days a week “Monday, Wednesday, Friday”, when treatment was administered. (**B**) Average TGF-β1 expression level, as evaluated by densitometry and Western blot protein analysis,   which was normalized to the untreated group and expressed as the mean ± SEM (*N* = 6). Significant differences were observed between treatment vs. untreated groups (*p < 0.05, **p < 0.01).**C**) Masson’s trichrome staining of scar ear tissues under diverse treatment conditions. Note: The red arrow shows the site of the magnified image. Scale bars = 100 μm. **D**) Graphic depiction of scar elevation index (SEI). **E**) Mean ± SEM of composite SEI in different treatment groups (*N* = 6). Significance differences between treatment groups and controls are indicated by *p < 0.05, **p < 0.01, ns = not significant). Permission was received from ref [172]

Many people develop abnormal scars (e.g., keloid and hypertrophic scars) each year due to reasons such as burns, surgery, and other skin trauma [172]. Abnormal scars are distinguished by redundant collagen deposition at the wound healing position. Several studies have shown that transforming growth factor beta 1 (TGF-β1) promotes a signaling cascade during wound healing that leads to fibroblast recruitment to the traumatized position, extracellular matrix production, and constriction for wound closing [172, 173]. Dysregulation of TGF-β1 has an effect on the formation of abnormal lesions. Ponedal et al. [172] developed two TGF-β1- targeting SNA nanoplatform, in which antisense oligonucleotides of TGF-β1 containing thiol or tocopherol terminal groups were grafted on the surface of gold-core SNAs (Au-SNAs) or liposome-core SNAs (LSNAs), respectively. The results revealed that both SNA constructs effectively decreased the expression levels of TGF-β1 protein in rabbit primary hypertrophic and keloid scar fibroblasts (in vitro), scar-derived human abnormal scar cells (human hypertrophic scar-derived fibroblasts [HSF], and human keloid scar-derived fibroblasts [KF]. Also, topical application of the mentioned SNAs on a rabbit ear model induced TGF-β1 downregulation at the administration site. Therefore, SNAs were highlighted as localized and noninvasive therapeutic agents in skin-related and other diseases (e.g., pulmonary and hepatic fibrosis) characterized by TGF-β1 overexpression (Table 2; Fig. 31) [172].

Synergistic function of tumor necrosis factor-a (TNF-a) and IL-17 A plays an important role in increasing the production of chemokines, antimicrobial peptides, and other cytokines from keratinocytes as well as local immune cells [174]. Studies have shown the up-regulation of TNF-a in the initiation and persistence of psoriasis, an immune-mediated inflammatory and chronic skin disorder [174]. So, Lewandowski et al. [174] developed a TNF-targeting antisense spherical nucleic acid that contained a liposomal core (50-nm diameter) (TNF-LSNA). Its suppressor function was evaluated in a human 3D cytokine-induced raft model, produced by the addition of TNF-a, IL-17 A, and IL-22, and in a psoriasis-like mouse (6-week-old C57BL/ 6 male mice) model, generated via the topical presentation of imiquimod (IMQ), which is a TLR7/8 ligand and a potential activator of the immune system. TNF-LSNAs entered into TNF-induced human psoriatic skin and silenced TNF-α mRNA expression. Furthermore, TNF L-SNA caused reverse development of transcription, histological, and phenotypes in 3D human psoriatic skin and IMQ-induced psoriasis-like mouse. Therefore, TNF L-SNA was suggested as a potential therapeutic tool (topically applied SNA-mediated antisense therapy) for psoriasis (Table 2) [174].

Another psoriasis pathogenesis mechanism is Th17 signaling, which is initiated with IL-17 A binding to its receptor (IL17RA) [175]. Previous studies have shown the upregulation of IL17RA in keratinocytes and immune cells during the development of psoriasis [175]. Liu et al. introduced an SNA nanoplatform consisting of antisense oligonucleotides targeting IL17RA and liposomal core NPs (IL17RA L-SNAs) for topical administration. IL17RA L-SNAs efficiently suppressed the expression of Il17ra, normalized the level of numerous psoriasis-related immune and proliferation markers that were overexpressed in the IMQ-induced psoriasis-like mouse model (e.g., Tnfa, S100a7a, Defb4, Il17c, Il6, Pi3, and Krt16), and decreased keratinocyte differentiation markers (e.g., Krt10, Lor). Overall, Il17ra L-SNA improved the clinically, histological, and transcriptional features of IMQ-induced psoriasis in a mouse model. Also, in human keratinocytes and 3D rafts, IL17RA L-SNAs significantly inhibited the expression of IL-17RA in a dose-dependent manner. Moreover, it notably decreased the expression of the genes encoding psoriasis-related cytokine and antimicrobial peptides, which were directly stimulated by the IL-17 pathway (e.g., PI3, DEFB4, IL17C, and TNFA). Eventually, IL17RA L-SNAs were suggested as a promising topical delivery platform for IL17RA targeted therapy for psoriasis. Furthermore, it is suggested that combinational L-SNA that targets both TNFA and IL17RA simultaneously may mitigate the course of the disease and improve treatment efficiency in severe cases of psoriasis (Table 2) [175].

**Fig. 32:**
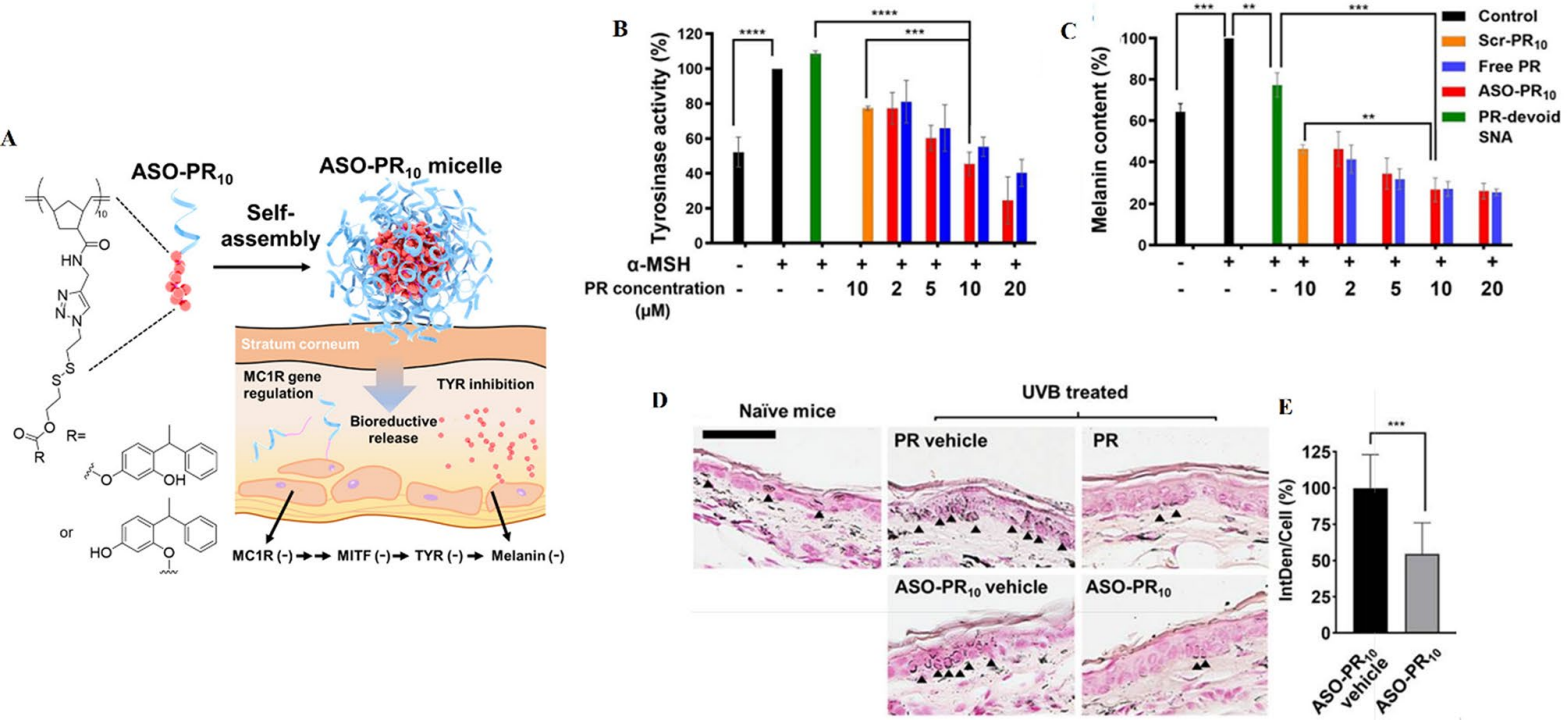
(**A**) Schematic illustration of ASO-PR10 prodrug conjugate formulation and their micellar SNA assemblage. (**B**) the activity of tyrosinase and (**C**) the content of melanin in various groups (treatment with ASO-PR10, DNA-PR10 with a scrambled sequence [Scr-PR10], PR-devoid micelles [in PBS], or free PR [in DMSO]. Note: a-MSH was applied for melanogenesis stimulation. The activity of tyrosinase and the content of melanin were documented as a percent of the difference in α-MSH-treated cells (2 to 20 µM PR, 48 h treatment). PR-devoid micelles: 1 µM ASO. (**D**) Histological study of isolated mouse ear following treatment with PR/ASO-PR10/carrier groups (paraffin-embedded). Note: Melanin (indicated by black arrows) is stained by Fontana − Masson staining. Scale bar: 50 μm. (**E**) Relative MC1R levels in ASO-PR10-treated group compared to ears treated with nanocarrier were determined via immunohistostaining. *** *p* < 0.001 (two-tailed test). Permission was received from ref [176]

Excessive melanin deposition in the skin causes hyperpigmentation (darkening of the skin compared to normal adjacent areas), a usually harmless condition developed by exposure to ultraviolet (UV) light/skin inflammation/skin injuries [176]. Melanocortin 1 receptor (MC1R) activation triggers signaling pathways within melanocytes that stimulate tyrosinase (TYR) and other tyrosinase-related proteins (TRPs), leading to melanogenesis (eumelanin) [176]. Fang et al. [176] developed an SNA-based micellar NPs containing a tyrosinase inhibitor prodrug (Phenylethyl resorcinol [PR]) core and antisense oligonucleotide (ASO) targeting MC1R shell for topical hyperpigmentation therapy. In this SNA nanoplatform, synergistic activity of ASO and PR increased cellular uptake, improved drug solubility, and enhanced skin penetration. Upon internalization, (1) MC1R antisense oligonucleotide inhibited new TYR synthesis via RNase H-mediated mRNA degradation with no releasing from SNA construction, and (2) PR prodrug bio-reductively was released and deactivated TYR. Additionally, ASO- PR10-SNA reduced the content of melanin in B16F10 melanoma cells and potentially induced an anti-melanogenic effect in a UVB irradiation-induced hyperpigmentation mouse model (Table 2; Fig. 32) [176].

Toll-like receptors (TLRs) comprise a crucial class of receptors that mediate the initiation of innate immune responses by recognizing conserved molecular patterns of diverse pathogens and activating NF-κB and IRFs transcription factors [177]. Therefore, TLRs, both those at the cell surface (TLRs 1, 2, and 4 − 6) and those within the endosomes (TLRs 3 and 7 − 9), are considered as attractive therapeutic targets [178]. On the other hand, activation of multiple TLRs has been shown in complex diseases (e.g., activation of TLR3 and TLR4 in rheumatoid arthritis, and TLR4 and TLR9 in liver fibrosis) [178]. In such cases, simultaneous inhibition of TLRs is required to prevent the activity of the immune system [178]. According to this, Ferrer et al. [178] designed dual TLR-targeting LSNAs consisting of unilamellar liposomal NP cores, the ssDNA shell of TLR9 inhibitory oligonucleotide (INH-18), and an inhibitory molecule (TAK-242) that was encapsulated within the liposomal core for suppressing TLR4. The DNA shell of the L-SNA allows receptor-mediated internalization of inhibitory oligonucleotides and small inhibitory molecules into cells. The inhibitory potential of LSNAs were evaluated in engineered HEK293 cell lines (HEK-Blue), steadily expressing either mouse TLR4 or mouse TLR9 and an NF-κB reporter gene. LSNA platform, while showing biocompatibility, made possible the co-delivery of two inhibitory factors and increased the inhibitory capacity of TLR relative to the linear sequence (up to a 10- fold) and compared to TAK-242 alone (1000-fold) (Table 2) [178].

Long non-coding RNAs (lncRNAs) are RNA molecules with more than 200 nucleotides in length that lack the potential to encode proteins [179]. Previous evidence shows that lncRNAs are extensively expressed and contribute to cellular processes, particularly in gene regulation by affecting their transcription, mRNA splicing, and mRNA turnover and translation [180]. The expression of a wide variety of lncRNAs is well-regulated [180]. Evidence indicates that alteration of lncRNAs expression is involved in development of numerous diseases and tumorigenic processes [109, 179, 180]. So, for the first time, Sprangers et al. [109] proposed a successful liposomal SNA nanoplatform for targeting and knocking down the nuclear-retained lncRNA Metastasis Associated Lung Adenocarcinoma Transcript 1 (Malat1), an oncogenic lncRNA involved in the metastasis of several types of cancer. In Malat1-PS-LSNAs nanoplatform, liposomal core NPs were made from biocompatible materials such as 1,2-dioleoyl-sn-glycero-3-phosphocholine (DOPC) and tocopherol-terminated oligonucleotides. Then they were functionalized with phosphorothioate (PS) antisense oligonucleotides (ASOs), a type of chemistry modified oligonucleotides that have a sulfur (S) atom in the non-bridging oxygen of the traditional phosphate backbone of oligonucleotides. PS-LSNAs construction was functionally assessed in human adenocarcinoma cell line (A549 cells). Data suggested that the lipid cores unlike gold nanoparticles (containing covalent chemistry) allowed for non-covalent assembly, which in turn conferred more dynamics to the structure and facilitated internalization. Indeed, the covalent assemblies were unable to enter the nucleus, possibly due to large size and static structure. Moreover, Phosphorothioate (PS) antisense oligonucleotides facilitated LSNAs entry via the Ras-related nuclear (RAN) protein-mediated pathway, which was different from its unmodified oligonucleotides. This allows LSNAs to be localized within the nucleus, enabling targeting and knocking down the complementary lncRNA within the nucleus. As shown, Malat1 was effectively silenced (up to 90%) in cultured cells treated with PS-LSNAs without toxicity. Furthermore, it was indicated that the inhibition of Malat1 led to upregulation of the downstream tumor suppressor (e.g., interferon-induced protein with tetratricopeptide repeats 2 [IFIT2]), verifying the capacity of PS-LSNAs as therapeutics and biological tools (Table 2) [109].

**Fig. 33:**
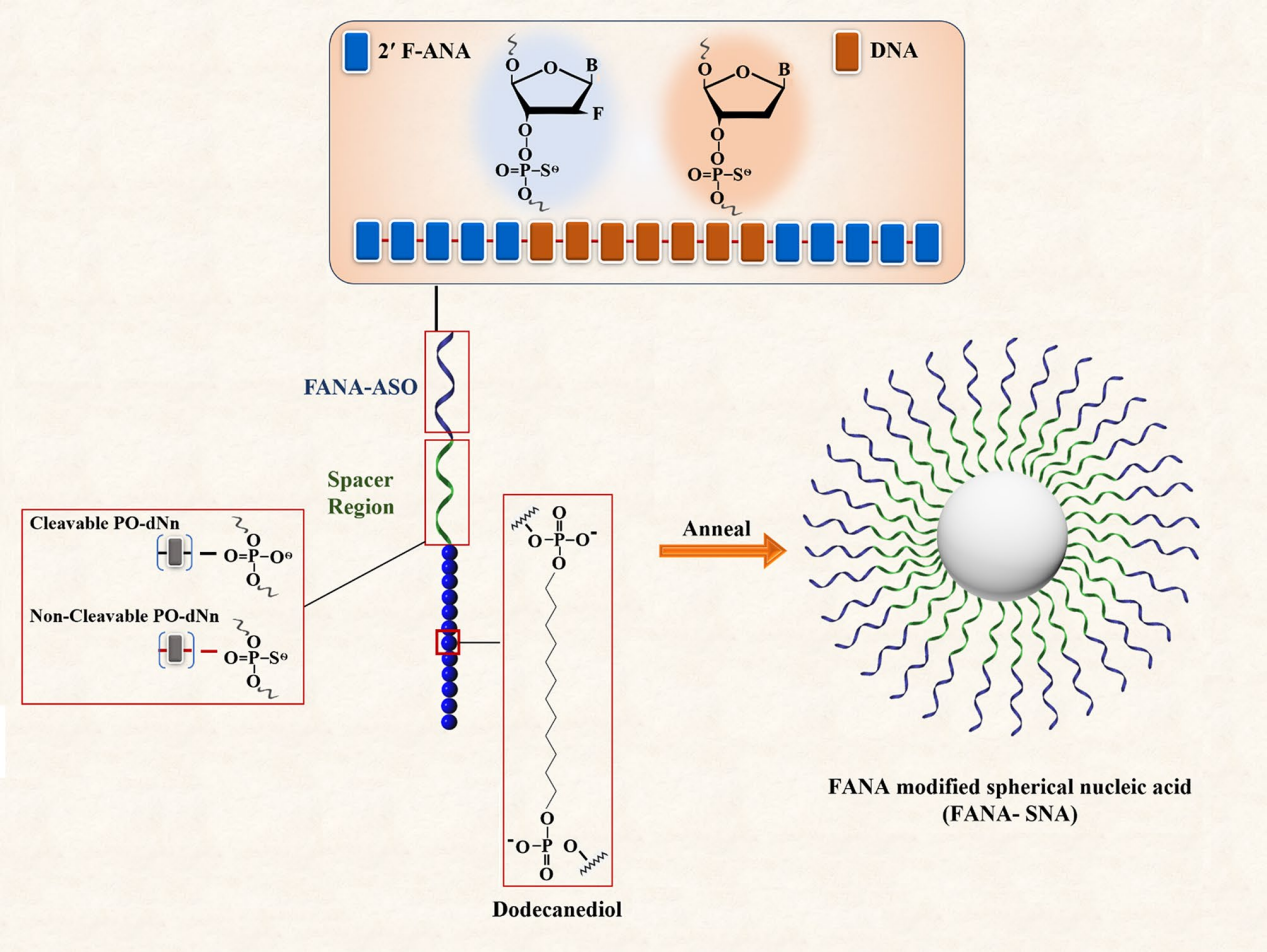
A model for FANA-SNA. Hybridizing dodecanediol (20 units) to FANA-ASO gapmer (18 nt) makes an SNA with a low polydispersity and well-defined size and shape. The spacer region is modified to optimize the activity of SNAs. (N, n, PO, and PS represent nucleotides, the number of nucleotides, phosphodiester backbone, and phosphorothioate backbone respectively). This figure was redrawn with permission from ref [181]

Fakih et al. [181] introduced FANA-SNAs, in which antisense oligonucleotides containing 2’-deoxy-2’-fluoro-D-arabinonucleic acid (2’F-ANA) sugar modification (FANA-ASO) were assembled on the micellar core by covalently attaching to twelve [dodecanediol phosphate] units. In the FANA-ASO construction, 8 nt DNA was surrounded by 5 nucleotides (nt) FANA on both sides. Previously, it was indicated that FANA offered advantages in enhancing gene silencing activity, nuclease enzyme resistance, and reduceding side-effects of immune-stimulatory responses. Additionally, this study confirmed that cleavable (phosphodiester [PO] vs. phosphorothioate [PS] backbone) and shorter (4- vs. 8-nucleotide) phosphate spacers were important in achieving better activity of SNAs inside the cell (Fig. 33). The performance of FANA-SNAs nanoplatforms was evaluated with FANA-SNA targeting survivin mRNA, FANA-SNA targeting APOB mRNA, and FANA-SNA targeting luciferase at the cellular level. Results exhibited that this nanostructure had more potency for the delivery of naked oligonucleotides (gymnosis) and gene silencing (70% silencing potency vs. 60%) compared to the free FANA-ASO. However, this amount can probably be increased by altering the chemistry of the hydrophobic polymer in the SNA core. Also, nuclear localization of the dye indicated the capability of PS LSNAs for ASOs delivery into the nucleus. It is worth mentioning that no appreciable toxicity was observed by any of FANA-SNAs nano-constructs (Table 2).

A type of self-escaping micellar-like SNA was developed by Shi et al. [49] This system consisted of Bcl-2 (anti-apoptosis protein) targeting antisense oligonucleotides and aggregation-induced emission (AIE) photosensitizer (PS) nanoparticles (TBD-PEG-N3). Upon light irradiation, the required amount of O_2_ for lysosome interruption was produced by AIE PSs core of BCL_2_-SNA, then Bcl-2 ASOs was released into the cytoplasm to direct gene regulation by targeting Bcl-2 mRNA. Indeed, AIE-based core-shell provides adequate endo/lysosome escape capacity without any cationic carrier and improves photodynamic therapy (PDT) effectiveness [49]. In this nanoplatform, ASOs coupled with TBD-PEG-N3 NPs showed better stability against DNase I degradation compared to the linear counterparts. In vitro assessments in HeLa and MCF-2 cells showed the successful function of Bcl-2 SNA (under light irradiation) in downregulating the Bcl-2 gene. So, the expression level of Bcl-2 mRNA in HeLa cells treated with Bcl-2 SNA under light irradiation decreased by 70% compared to untreated cells. While in cases of Bcl-2 SNA without light irradiation (39%) or Bcl-2 ASOs (500 nM) transfected using Lipo2000 (48%), the decrease in gene expression was less prominent. In vivo activity of SNA was evaluated in HeLa-cell xenografted mice models. Data illustrated that Bcl-2 SNA was efficiently taken up by tumor cells, escaped from endo/ lysosomes under light irradiation,  and inhibited tumor growth by 95%, ultimately promoting cancer cell apoptosis (Table 2) [49].

#### Plasmid DNA (pDNA)

pDNA is a closed circular, double-stranded DNA molecule with a size range of 1 to over 200 kb. They originate from bacteria but can be produced in large quantities through fermentation of bacterial cultures [182]. pDNAs are capable of carrying recombinant therapeutic genes into cells and organs [183]. In addition, plasmid DNA has other sequences, including promoter/enhancer elements, which are needed to control the transcription and expression level of the encoded protein after being introduced into the target cell [182]. Indeed, in clinical trials, pDNA containing therapeutic sequence(s) utilizes cellular machinery to produce the desired RNA for modifying protein production [184]. Several viral and nonviral gene delivery methods have been utilized for plasmid DNA-based therapeutic gene delivery to cells [182]. However, there are still some challenges with pDNA delivery, including poor cellular uptake, premature material separation, and ineffective responses [184].

#### Spherical nucleic acids delivery nanoplatforms for plasmid DNA (pDNA)

Dizaji et al. [185] synthesized a type of core/shell nanoplatforms for pDNA delivery, in which iron oxide magnetic silica (Fe3O4@SiO2) NPs with 50 nm mean diameter were created by the microemulsion method. Then, Fe3O4@SiO2 NPs were functionalized with negatively charged pDNA encoding EGFP. Poly (allylamine hydrochloride) (PAH), an amine-rich cationic polyelectrolyte material with high water solubility, was utilized for conferring positive charge to the surface of Fe3O4@SiO2 NPs, which allowed it to carry pDNA into target cells. The Fe3O4@SiO2/PAH/pDNA nanoplatform revealed improved cellular uptake and efficient gene expression and also protected pDNA against lysosomal degradation by a physical shield, caused by the electrostatic attaching of a cationic polymer (PAH) to pDNA. Furthermore, Fe3O4@- SiO2/PAH showed low cytotoxicity even at high concentrations. Regarding these, Fe3O4@SiO2/PAH nanostructure was suggested as an excellent candidate gene delivery system that could have a high potential in cancer therapy (Table 2) [185].

**Fig. 34:**
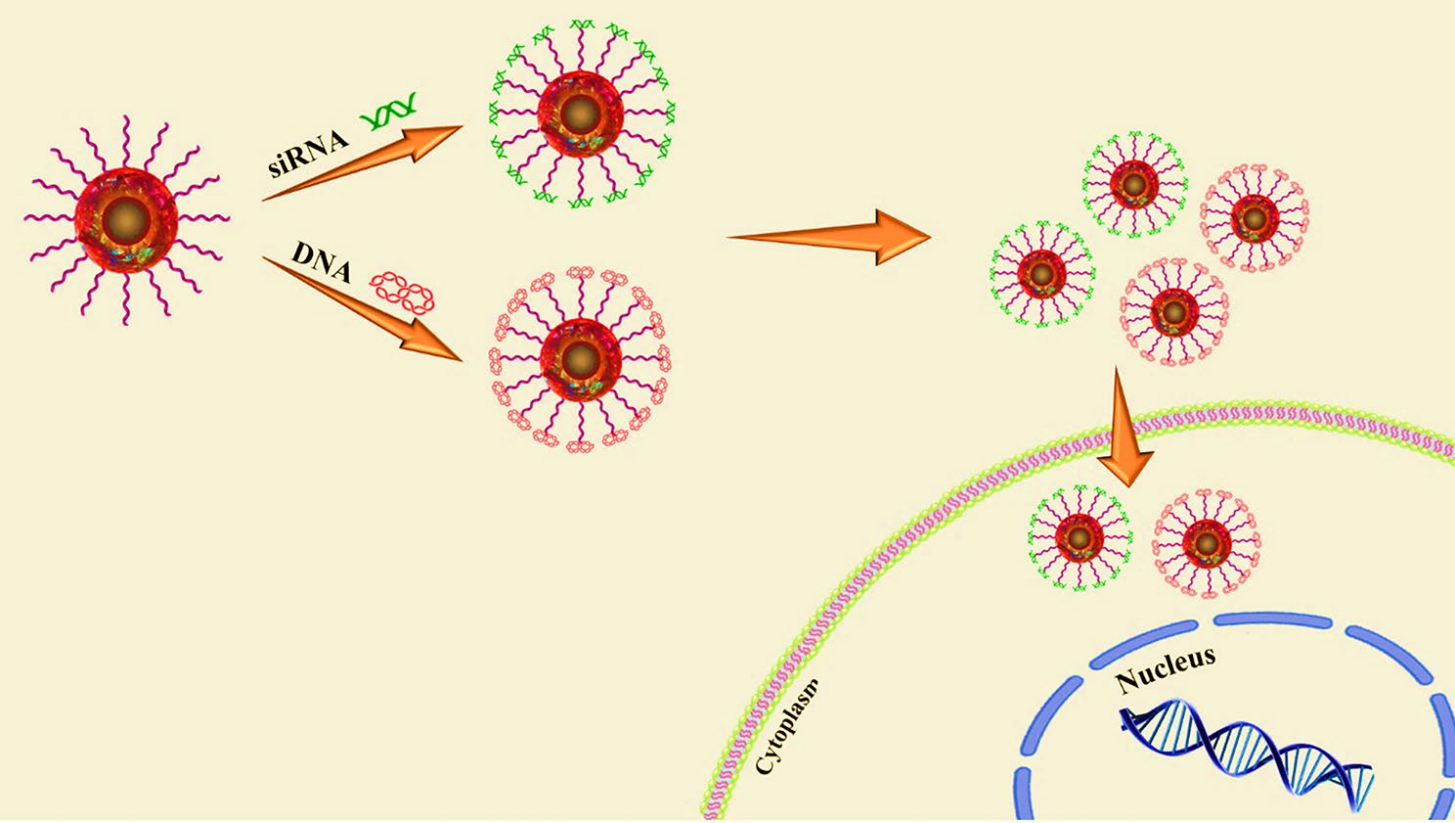
Schematic illustration of Alkyl-PEI2k-Cdots/siRNA complex formation and delivery into the cancer cell. This figure was redrawn with permission from ref [186]

In another study by Liqin Wang et al. [186], Alkyl- polyethylenimine 2k-Carbon dots (Alkyl-PEI2k-Cdots) were presented as an efficient nano-composite formulation with low cytotoxicity and photonic properties for in vivo gene delivery. In this work, Alkyl-PEI2k-Cdots/siRNA complexes were designed for targeting luciferase pDNA directly administrated into fluc-4T1/4T1 xenograft tumor tissues via Alkyl-PEI2kCdots/pDNA complexes. Effective delivery of Alkyl-PEI2kCdots/pDNA into these cells led to efficient luciferase gene expression. The suppression of luciferase expression was observed following the injection of Alkyl-PEI2k-Cdots/siRNA complexes (Table 2; Fig. 34) [186].

#### Immunotherapy

Immunotherapy is a kind of biological therapy that uses specific parts of the immune system to deal with diseases such as cancer and can be accomplished in several ways: (1) by stimulating and activating existing body’s immune components for finding and attacking tumor cells, and (2) using synthesized materials that act like immune system components to improve the function of the immune system in finding and attacking tumor cells.

The previous sections mentioned that SNA’s nanoplatforms use the antisense technology. So, the therapeutic effects are manifested by a logical Watson Creek complementary base pairing with the downstream target disease-causing genes, degradation target mRNA products, and/or preventing translation. However, other types of SNA nanoplatforms containing shells of CpG oligonucleotide sequences have also been reported for the treatment of various diseases, specifically cancer.

CpG ODNs (Cytosine-phosphorothioate-guanine oligodeoxynucleotides) are short synthetic single-stranded DNA molecules containing unmethylated CpG dinucleotides motifs [187]. They are known as a class of Th1-type immune stimulatory factors that interact with toll-like receptor 9 (TLR9) [188]. Therefore, they can promote intracellular signaling, leading to the activation of immune cells (e.g., dendritic cells [DC9], macrophages, and B cells) and the production of immunoglobulins, chemokines, and pro-inflammatory cytokines [188]. Subsequently, produced cytokines (e.g., IL-12) stimulate naive T cells to differentiate into cytotoxic T-cells (CTL) and T helper 1 (Th1) [188]. Generally, CpG motifs interact with TLR9 and trigger innate immune responses. Nucleic acid sequences on SNAs also affect immune responses toward SNAs [136]. Oligonucleotide sequences of SNAs can affect immune response rate. As shown, G-rich sequences activate macrophages much more than T-rich sequences [136]. Therefore, it has been suggested that SNAs can be used for immunotherapy by adding G-rich sequences to the oligonucleotide shell or by directly attaching them to the surface of SNA [136].

#### Spherical nucleic acids delivery nanoplatforms in immunotherapy

Cole et al. developed animmune-stimulatory SNAs nanostructures containing a dense shell of CpG-rich oligonucleotides and a liposomal NP core encapsulating Triple Negative Breast cancer (TNBC) antigen, which is involved in directing the anticancer immune responses against TNBC cells. The recently mentioned nanostructure showed a 50% increase in the co-delivery of the antigen and immune system stimulating factor compared to delivering of the antigen and oligonucleotide separately. In comparison with the linear oligonucleotides, SNAs remained longer in tumor tissues after a hypodermic injection (24 h relative to 1 h). A 30% decrease in final tumor volume was also observed in tissues treated with SNA. Immune-stimulatory SNA resulted in the elevation of T-cell lymphocyte count and a reduction in the myeloid-derived suppressive cell (MDSC) quantity in TNBC tumors’ microenvironment, reflecting the immune-stimulatory role of SNAs in changing the immunosuppressive microenvironment of TNBC tumors. Furthermore, encapsulated TNBC-specific antigens within the immune-stimulatory SNA core led to the generation of an antigen-specific response [189].

Callmann et al. [190] developed liposomal spherical nucleic acid (SNAs) nanocarriers for TNBC immunotherapy. They consisted of a liposomal core containing lysate derived from a TNBC cell line (as antigens) and a layer of CpG-1826 immunostimulatory oligonucleotides (as adjuvants). Lys-SNAs nanostructures improved the codelivery of immunostimulatory nucleic acids and antigen to immune cells relative to free linear nucleic acids in mixtures of lysates both in vitro and in vivo and diminished tumor growth compared to simple lysate mixtures and free CpG-1826 and in TNBC syngeneic mouse models. Moreover, SNAsʹ capacity for dendritic cell activation was significantly increased when TNBC cells were oxidized before lysis and encapsulation within SNAs (OxLys-SNAs) compared to their non-oxidized counterparts. Also, in-vivo administration of OxLys-SNAs significantly increased cytotoxic CD8 + T cells and decreased myeloid-derived suppressor cells (MDSCs) in the tumor microenvironment relative to other groups (simple mixtures of oxidized lysates with CpG-1826 and Lys-SNAs). As a result, OxLys-SNAs were suggested as a class of immunotherapeutic agents with a significant antitumor activity that could confer long-term survival to animals bearing resistant tumors (Table 2) [190].

Radovic-Moreno et al. [191] developed two types of immunomodulatory spherical nucleic acids (SNAs) that could either stimulate (immunostimulatory [IS]-SNA) or regulate (immunoregulatory [IR]-SNA) immune responses by agonizing or antagoniz endosomal toll-like receptors (TLR3, TLR7/8, and TLR9). IS-SNAs are made up of a nanoparticle core (Au or liposomal) and TLR9 agonist (phosphorothioate [PS] or phosphodiester [PO] CpG) oligonucleotides. IS-SNAs, compared to free oligonucleotides, showed higher antibody titers (700-fold), higher cellular responses (400-fold), increased inhibition potency of RAW 264.7 macrophages (80-fold), and improved survival of mice models of lymphomas. IR-SNAs comprised of (Au-SNA or L-SNA) and TLR9 antagonist oligonucleotides (4084 F) and displayed 8-fold increases in potency and 30% more diminution in fibrosis score in nonalcoholic steatohepatitis (NASH) bearing mice. As a result, SNAs were considered as attractive immunotherapy tools due to their ability to be used in clinical therapeutic applications, having a specific chemical structure, inducing sequence-specific TLR activation, and showing good stability (Table 2) [191].

**Table 2:** Applications of various nanoplatforms of SNA as carriers in delivery systems

Nano-platforms	Oligonucleotide shell	Disease model	Cell line tested	Assayed genes	Assayed conditions	Refs
Alkyl-PEI2k-Cdot	siRNA /plasmid DNA (pDNA)	Breast cancer	4T1	Luciferase gene	Invitro/in vivo	[186]
Gal-PEI-SPIO	siRNA	Hepatocellular carcinoma	Hepa1-6 (Hepatoma)	c-Met	Invitro/ in vivo	[1]
Lipidoid-Coated Iron Oxide	DNA and siRNA	–	HeLa cells	Luciferase	Invitro	[90]
LNP-Luc-SNA	siRNA	–	RAW 264.7-Lucia ISG, U87-MG-Luc2	Luciferase	Invitro/ in vivo	[58]
Adr-siRap2b-Au-SNA	siRNA	Colorectal cancer	HCT116, MCF-7	Rap2b	Invitro/ in vivo	[3]
siGM3S-Au-SNA	siRNA	Diabetic skin wound	Keratinocytes	Ganglioside-monosialic acid 3, IGF1R, EGFR	Invitro/ in vivo	[144]
siBcl2L12-Au-SNA	siRNA	Glioblastoma	U87MG, LNZ308, and LNZ235	Bcl2L12, caspase-3, caspase-7	Invitro/ in vivo	[145]
siGli-SNA	siRNA	Glioblastoma	U87MG, SF767, GIC-20	Gli1, Smo, CyclinD1, c-Myc, Bcl2, and ABCG2	Invitro	[146]
siHIF1α- Bcl-2(ASO)- PNA- photolabile SNA	siRNA/ASO	Cervical cancer	HeLa	HIF-1α, Bcl-2	Invitro/ in vivo	[147]
siGFP	siRNA	glioma	U87-MG	GFP	Invitro	[138]
siTNFa-Au-SNA	siRNA	Inflammatory diseases	Murine macrophage RAW264.7	TNF-α	Invitro	[148]
siGFP/siGAPDH-Au- SNA(s)	siRNA	–	Hela, C166 GFP-expressing	GFP, GAPDH	Invitro	[149]
hairpin-like siRNA-SNA	siRNA	Ovarian cancer	SK-OV-3	HER2, Luc, VEGF	Invitro	[150]
siAR- Au-SNA	siRNA	Prostate cancer	Lymph node carcinoma of the prostate (LNCaP)	Androgen receptor	Invitro/ in vivo	[151]
siEGFR- Au-SNA	siRNA	Skin-related diseases	Human keratinocyte	EGFR	Invitro/ in vivo	[192]
Carbon-siPlk1-SNA	siRNA	Breast cancer, melanoma cancer	MCF-7, A375	Polo-like kinase-1	Invitro/ in vivo	[153]
siIKKβ-Au-SNA	siRNA	Islet transplantation	Islets cells	IκB kinase (IKK) subunit (IKKβ) of NF-κB	Invitro/ in vivo	[154]
GL-3 siRNA- DNA nanoclew (DC) -SNA	siRNA	–	HeLa-Luc	Luciferase	Invitro	[1]
miR-182-Au-SNA	microRNA	Glioblastoma	Gliomas	Caspase, p53 inhibitor, Bcl2L12, c-Met, and HIF2A	Invitro/ in vivo	[160]
miR-182-Au-SNA	microRNA	Glioblastoma	LN229, LNZ308, U87MG	Bcl2L12, caspase-3, caspase-7, Ki67	Invitro/ in vivo	[160]
miR-34a-Reporter-Fuel-SNA	MicroRNA	Breast cancer	MCF-7	miR-34a, survivin	Invitro	[161]
Anti-miR21-nanoflare-Au-SNA	MicroRNA	Prostate cancer	PC-3	miR-21	Invitro	[162]
Anti-miRNA-92b-ApoE/ RVG- Liposomal SNA	MicroRNA	Glioblastoma	U87 GBM	miRNA-92b	Invitro/ in vivo	[163]
Anti-miR21-polyA-Au-SNA	MicroRNA	Breast cancer	HEK293T, 293T, MCF-7	miR-21, TPM, EGFP	Invitro/ in vivo	[164]
Anti-miR99b-Au-SNA	MicroRNA	Sepsis	RAW264.7	miR-99b, MFGE8	Invitro/ in vivo	[166]
Anti-miR-34a -Au-photoresponsive SNA	MicroRNA	Breast cancer	MCF-7	miR-34a, Notch, LMTK3, survivin	Invitro	[167]
miR-145-Au-SNA	MicroRNA	Prostate cancer, breast cancer	MCF-7, PC3	miR-145	Invitro	[168]
miR-130b-Au-SNA	MicroRNA	Multiple myeloma	MM.1 S	GR-α, miR-130b	In vitro	[169]
*TGF-β1*- Au /Liposomal-SNA	ASO	Abnormal scars	HSF, KF	TGF-β1	In vitro/ in vivo	[172]
*TNF-α* -Liposomal-SNA	ASO	Psoriasis	Keratinocytes	*TNF-α*	In vitro /ex vivo/ in vivo	[174]
*IL17RA*-Liposomal-SNA	ASO	Psoriasis	Keratinocytes	Il17RA, Tnf-*α*, S100a7a, Defb4, Il17c, Il6, Pi3, and Krt16, Krt10, Lor	In vitro /ex vivo/ in vivo	[175]
*MC1R*- PR10- micellar-SNA	ASO	Hyperpigmentation	B16F10	TYR, *MC1R*	In vitro/ in vivo	[176]
*TLR*-TAK 242-Liposomal-SNA	ASO	Immune disorders	HEK293	TLR4, TLR9, NF-κB, TLR	In vitro	[178]
Malat1- PS-LSNAs	ASO	Adenocarcinoma	A549	Malat1 (lncRNA), IFIT2	In vitro	[109]
APOB -FANA-SNA Survivin- FANA-SNA Luciferase- FANA-SNA	ASO	Breast cancer, cervical cancer, liver cancer	HeLa, HepG2, MCF-7, MDA-453	APOB, Survivin, Luciferase	In vitro	[181]
BCL_2_- AIE PS-SNA	ASO	Breast cancer, cervical cancer	HeLa, MCF-2	BCL_2_	In vitro/ in vivo	[49]
hollow silica-based SNA	ASO	–	Mouse endothelial cells	eGFP	In vitro	[81]
Fe3O4@SiO2/PAH/pDNA	Plasmid DNA	Cervical cancer	HeLa cells	–	In vitro	[185]
Fe3O4/plasmid DNA	Plasmid DNA	–	PK-15	Genes encoding a green (DNAGFP) or red (DNADsRed) fluorescen	In vitro	[193]
Dual-Responsive γ-Fe2O3@PDMAEMA	Plasmid DNA (Plasmid pH2B-EGFP)	–	E. coli DH5 alpha strain	EGFP	In vitro	[93]
MGMT- ribozyme- Au − SNA	Ribozyme	Glioblastoma	T98G	MGMT	In vitro	[36]
Immune-stimulatory- CpG-rich- LSNAs	CpG-rich	Triple Negative Breast cancer (TNBC)	MDSC, T-cell lymphocyte	–	Ex vivo-in vivo	[189]
Immune-stimulatory-liposomal- lys-SNAs	CpG-rich	Triple Negative Breast cancer (TNBC)	CD8 + T, MDSC, dendritic cell	–	In vivo	[190]
Au/liposomal- TLR9 (IS/IR)- SNAs	CpG-rich-TLR9 agonist/ antagonist	Fibrosis score in nonalcoholic steatohepatitis (NASH)	RAW 264.7	–	In vivo	[191]

## Concluding remarks

Gene delivery systems, including viral and non-viral (physical, chemical), play a crucial role in developing gene therapy strategies. Designing gene delivery carriers should have high transfection efficiency, negligible toxicity, and minimal immunogenicity for in-vivo systems, presenting major challenges for introducing therapeutic oligonucleotides to a specific tissue or cell. Spherical nucleic acids offer a nanoparticle-based delivery system that consists of a nanoparticle core (e.g., gold, silica, liposome, etc.), which is functionalized with hydrophilic oligonucleotides (e.g., ASOs, microRNA, siRNAs, and immunomodulatory oligonucleotides). The distinctive three-dimensional structure of SNAs gives them unique properties and significant stability compared to linear nucleic acids. SNAs are stable against enzymatic degradation due to the presence of the negatively charged oligonucleotide shell surrounding the nanocarrier. This layer achieves stability through steric hindrance and interaction with positive-charged local salts [194]. In such a way, these single-positive-charged ions like Na + hinder the activity of DNase I and similar enzymes by displacing essential ions (e.g., Ca2 + and Mg2+) necessary for enzyme activity [104]. The cellular uptake of SNAs and their intracellular delivery are efficient and rapid, depending on the spherical arrangement of oligonucleotides [44]. One of the main challenges in the systemic delivery of nanocarriers to tumor microenvironments and the central nervous system (CNS) to achieve oligonucleotide-based therapies is the presence of blood-brain barriers (BBB) ​​and blood-tumor barriers (BTB). The internalization of SNAs happens through scavenger A receptors without needing any ligands or auxiliary agents. Scavenger receptors are highly expressed on epithelial cells. As a result, they can cross the epidermis, BTB, and BBB in contrast to other oligonucleotide-based therapeutics.

As a brief summary, SNAs are an emerging class of nanocarriers with a unique construction that can be functionalized with therapeutic nucleic acid strands to achieve therapeutic objectives. They can enter almost every known cell type in large quantities without using transfection agents. Additionally, they can cross the epidermis, as well as the \BTB\ and \BBB\. Moreover, SNAs are excellent candidates for systemic delivery because they are nuclease-resistant and do not stimulate immune responses. They can also be tuned by receiving additional modifications to exert their effects on specific tissues or for the co-delivery of therapeutic and drug oligonucleotides.

To date, various types of SNA-based treatments with diverse platforms have been investigated in pre-clinical phases (cellular, animal), and acceptable results have been obtained. So far, clinical studies conducted in this field are infrequent. Recently, for the first time, a nano platform of SNA (siBcl2L12-SNAs) was evaluated in a human phase 0 clinical study after systemic administration, SNA was introduced as a potentially safe and BBB/BTB-penetrating approach for the treatment of glioblastoma [195]. As such, these remarks strongly inspire the development of SNA-based therapies.

## Future prospects

SNAs, an innovative delivery system, have enormous potential in transporting oligonucleotides, drugs, and proteins. So far, most of the studies in the field of SNA have been carried out in the pre-clinical stage, and only a limited number of clinical studies have been conducted so far. It is hoped that more clinical studies will be conducted in this field in the future. Despite many advantages of SNAs over other gene delivery carriers, such as low toxicity, resistance to enzymatic degradation, and auxiliary reagent-free uptake, this strategy still faces challenges to achieve high-efficiency therapeutic outcomes. Nearly limitless combination of nano-core materials and oligonucleotide sequences can be used for SNA preparation. Hence, achieving a precise and adequate formulation for SNA is a major challenge. In addition, important points regarding the construction of SNAs that should be taken into consideration in the future include designing SNA structures with the ability to form programmable corona proteins, resistance to endosomal degradation, safer and more accessible nanoparticle cores with easier processing, and most importantly, structures with the capacity for versatile self-assembling and disassembling. Hence, it is crucial to concentrate on this fascinating subject because it seems that these nanocarriers offer vast potential in nanomedicine, including diagnostic imaging, drug/ gene therapy, and immunotherapy.

## Data Availability

No datasets were generated or analysed during the current study.
